# The intriguing evolution of effect sizes in biomedical research over time: smaller but more often statistically significant

**DOI:** 10.1093/gigascience/gix121

**Published:** 2017-12-06

**Authors:** Paul Monsarrat, Jean-Noel Vergnes

**Affiliations:** 1 Paul Sabatier University, Dental Faculty, Department of Anatomical Sciences and Radiology, Toulouse University Hospital (CHU de Toulouse), 31062 Toulouse Cedex 9 and STROMALab, Université de Toulouse, CNRS ERL 5311, EFS, ENVT, Inserm, UPS, 31432 Toulouse Cedex 4, France; 2 Paul Sabatier University, Dental Faculty, Department of Epidemiology and Public Health, Toulouse University Hospital (CHU de Toulouse), 31062 Toulouse Cedex 9, France and Division of Oral Health and Society, Faculty of dentistry, McGill University, Montreal, Quebec, QC H3A 0G4, Canada

**Keywords:** meta-research, effect size, biomedical research, “publish or perish”, data mining

## Abstract

**Background:**

In medicine, effect sizes (ESs) allow the effects of independent variables (including risk/protective factors or treatment interventions) on dependent variables (e.g., health outcomes) to be quantified. Given that many public health decisions and health care policies are based on ES estimates, it is important to assess how ESs are used in the biomedical literature and to investigate potential trends in their reporting over time.

**Results:**

Through a big data approach, the text mining process automatically extracted 814 120 ESs from 13 322 754 PubMed abstracts. Eligible ESs were risk ratio, odds ratio, and hazard ratio, along with their confidence intervals. Here we show a remarkable decrease of ES values in PubMed abstracts between 1990 and 2015 while, concomitantly, results become more often statistically significant. Medians of ES values have decreased over time for both “risk” and “protective” values. This trend was found in nearly all fields of biomedical research, with the most marked downward tendency in genetics. Over the same period, the proportion of statistically significant ESs increased regularly: among the abstracts with at least 1 ES, 74% were statistically significant in 1990–1995, vs 85% in 2010–2015.

**Conclusions:**

whereas decreasing ESs could be an intrinsic evolution in biomedical research, the concomitant increase of statistically significant results is more intriguing. Although it is likely that growing sample sizes in biomedical research could explain these results, another explanation may lie in the “publish or perish” context of scientific research, with the probability of a growing orientation toward sensationalism in research reports. Important provisions must be made to improve the credibility of biomedical research and limit waste of resources.

## Background

Effect sizes (ESs) are useful to describe associations in studies that focus broadly on associations between variables [1]. In medicine, ESs allow the effects of independent variables (including risk/protective factors or treatment interventions) on dependent variables (e.g., health outcomes) to be quantified. There are many different types of ESs [2], but in human biomedical research, ESs are predominantly derived from risk (or rate) ratios (RRs), odds ratios (ORs), or hazard ratios (HRs) [3]. No longer confined to the early domains of epidemiological research (such as epidemiological oncology) [4], use of these estimates is now benefiting all biomedical research (e.g., environmental epidemiology [5], genetics [6], or interventional research [7]). As there is no straightforward relationship between *P*-values and strengths of association [2], adequate reporting of ESs is strongly recommended by recent statistical guidelines [8]. Given that many public health decisions and health care policies are based on ES estimates [9], it is important to assess how ESs are used in the biomedical literature and to investigate potential trends in their reporting over time. Consequently, in this study we aim (1) to describe the global use of ESs in the biomedical literature during the last 25 years, (2) to analyze their temporal evolution in terms of strength and statistical significance, and (3) to identify and discuss factors associated with potential evolutions.

## Data Description

PubMed is the most commonly used database of biomedical information [10] and was considered the primary source. A “Knowledge Discovery in Databases” (KDD) process led us to add the PubMed Central (PMC) database as an additional source of data, according to the aims and modalities described in the Knowledge checking subsection of the Methods section.

All PubMed citations were bulk-downloaded in XML format (2017 release dated 13 December 2016) from the FTP servers of the US National Library of Medicine (NLM). Among the 26 759 399 citations, 16 820 871 (63%) provided an abstract, and were thus considered preprocessed data (Additional Fig. S1A–C). A data mining process was then run to automatically detect ESs (OR, RR, HR) within PubMed abstracts, along with several characteristics of the abstracts (see details in the Methods).

## Analyses

Unless specified, the results presented are related to nonreview abstracts with 95% confidence intervals (95% CIs). Details may be found in the flow diagram of the selection process for abstracts (Additional Fig. S1C and Additional Table S4) and in the Supplementary Methods for identification of type of CI.

### Reporting of ESs increased greatly over time

Two point one percent of PubMed abstracts contained at least 1 ES. The relative proportions of ES reports increased markedly over time (Additional Fig. S2A). More than half of the ESs were ORs, with a trend for RRs to be substituted by HRs (Additional Fig. S2B). ESs >1 were still largely predominant, despite an increase of abstracts with all ESs <1, or with a mix of ESs >1 and ESs <1 (Additional Figs S2C and S3).

### Geographic and thematic disparities in reporting of ESs

**Figure 1:**
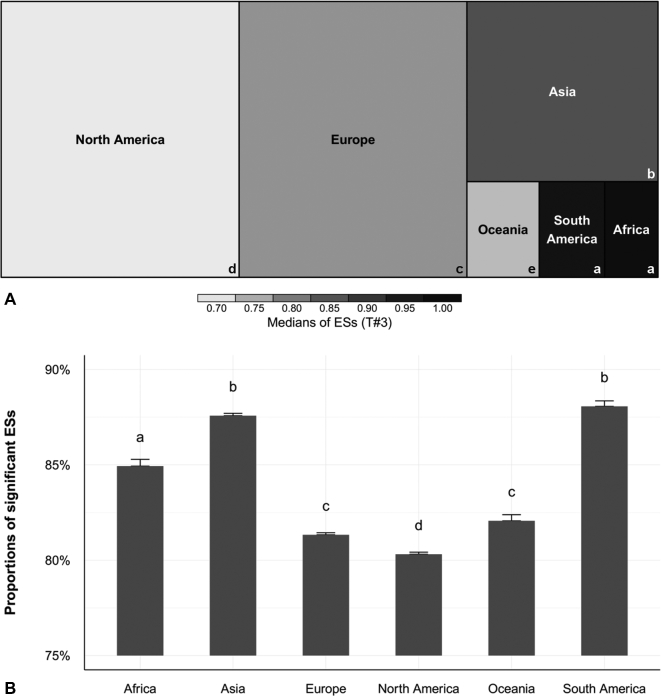
ESs are subject to geographic disparities. (**A**) Treemap of medians of ESs (T#3) by continent. All detected ESs were considered for the comparisons between continents. For each continent, the size of the rectangle is proportional to the absolute number of abstracts with at least 1 detected ES. ESs from abstracts with cross-continental affiliations (5.2% of abstracts) were counted in each continent concerned. The grayscale indicates median values of ESs (on a linear scale, T#3) by continent: lighter gray corresponds to lower ES values, and darker gray to higher ES values. In rectangles, different letters correspond to statistically different ESs (Kruskal-Wallis pairwise comparisons test). Europe and North America were by far the biggest providers of abstracts with ESs. ES values were higher in abstracts from South America, Africa, and Asia, and lower in abstracts from Europe, Oceania, and North America. Number of abstracts: 238 954. (**B**) Histogram of mean and standard error of proportions of statistically significant ESs per abstract, according to continent. Beneath the bars, different letters correspond to statistically different values (Kruskal-Wallis pairwise comparisons test). ESs were more likely to be statistically significant in abstracts from South America, Africa, and Asia.

**Figure 2:**
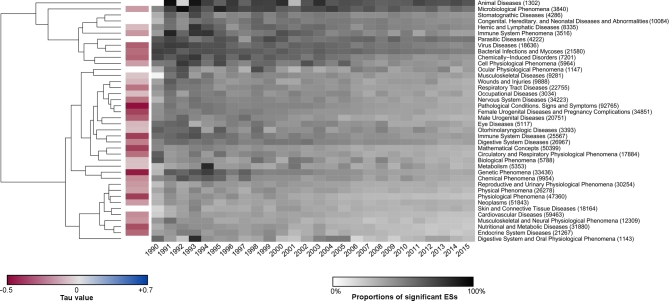
Heatmap of the temporal evolution of median ESs (T#3) by research field. ESs were considered at the abstract level, using the mean of ES(s) per abstract (on a linear scale, T#3). Abstracts were linked to specific research field(s) according to their (MeSH) keywords, so a single abstract could be linked to multiple fields of research (overall ratio: 801 839/229 581 = 3.49). Research fields (at the right of the figure) were defined from 2 main branches of the MeSH Tree (US NLM): [C] “diseases” and [G] “phenomena and processes.” Numbers in brackets are the total number of abstracts with at least 1 detected ES during the 25-year period in a specific research field. Three branches (out of 43) with fewer than 1000 abstracts with at least 1 ES were eliminated. Trends were calculated at the monthly level but are represented in the graph at the yearly level for readability. The grayscale indicates yearly median values of ESs (T#3): lighter gray corresponds to lower ES values, and darker gray to higher ES values. On the left, research fields are grouped using a hierarchical cluster analysis and represented as a dendrogram: higher ESs are found in fields dealing with infectious diseases (e.g., microbiological phenomena, virus diseases, bacterial infections and mycoses; see top of the figure). The color scale indicates the τ value of the evolution of monthly medians of ESs for each research field. Blank rectangles mean nonsignificant trends. Colored rectangles are red (not blue), with variable intensity indicating a significant monotonic downward trend of ESs in nearly all research fields. The most marked decrease is observed for genetic phenomena (τ = –0.52, *P* < 0.001). Number of abstracts: 229 581.

Europe and North America were by far the biggest providers of abstracts with ESs (Fig. 1A), although the number was growing considerably in Asia (Additional Fig. S2D). There were notable disparities in ES values among different geographical areas: they were higher in South America, Africa, and Asia, and lower in Europe, Oceania, and North America (Fig. 1A). ESs were more likely to be significant in regions where they were the highest (Fig. 1B, Additional Table S5). Higher ES values and proportions of significant ESs were found in fields dealing with infectious diseases (Fig. 2, Additional Fig. S4).

### ESs values are decreasing over time

**Figure 3:**
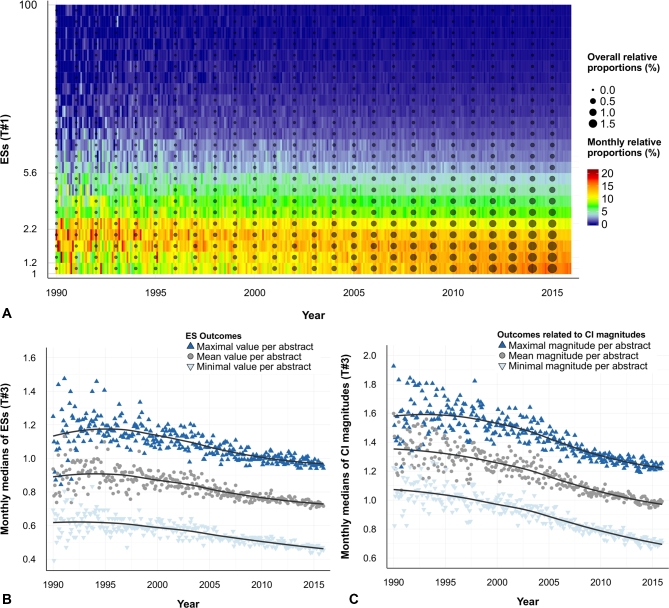
ESs are decreasing over time. (**A**) Heatmap of the temporal evolution of ESs (i.e., odds ratio, relative risk, or hazard ratio) on their original log scale (T#1) (see details in the Supplementary Methods). All detected ESs (n = 690 196) are considered. ESs <1 were transformed according to the T#1 transformation (inverse transformation) (Additional Table S3A). ES >1 were not transformed. The vertical axis corresponds to a logarithmic scale ranging from 1 to 100, with 25 regular cutoff values (ESs that were >100, corresponding to 0.16% of all detected ESs, are not reported on the graph). The color scale indicates the monthly relative proportion of ESs in each interval: cold colors correspond to lower proportions and hot colors to higher. We can see a trend toward a massive concentration of ES values near to 1 at present. The black dots represent the overall relative proportion of ESs, by year and by interval. We can see that the lowest ESs of the more recent abstracts are the most numerous ESs overall. (**B**) Scatter plot of the temporal evolution of monthly medians of ESs on a linear scale (T#3). ESs were considered at the abstract level (n = 247 339). Three different outcomes were considered: minimal, maximal, and mean of ES(s) of each abstract. The 3 temporal evolutions are decreasing, with τ values of –0.64 (*P* < 0.001), –0.59 (*P* < 0.001), and –0.63 (*P* < 0.001), respectively. (**C**) Scatter plot of the temporal evolution of monthly medians of confidence interval (CI) magnitudes on a linear scale (T#3). CI magnitudes were considered at the abstract level (n = 247 339). Three different outcomes are considered: minimal, maximal, and mean of CI magnitude(s) of each abstract. The 3 temporal evolutions are decreasing, with τ values of –0.76 (*P* < 0.001), –0.67 (*P* < 0.001), and –0.72 (*P* < 0.001), respectively.

A major finding was that ES values were decreasing over time. In Fig. 3A, there is a clear, progressive evolution between the 1990s and the 2010s, with a massive concentration of ES values nearer to the value 1 at the present time. This result was very robust, as the decrease was observed with all tested outcomes per abstract (i.e., minimal, maximal, mean transformed ES values) (Fig. 3B, C). It also concerned both “risk” and “protective” values (Additional Fig. S5A, B): overall medians of ES values for “risk” decreased from ES ∼ 2.50 in 1990–1995 to ES ∼ 2.11 in 2010–2015, and those for “protective” values from ES ∼ 0.59 to ES ∼ 0.63. The decrease was observed for all types of ESs, when analyzed separately (Additional Fig. S5C). It was also consistent with a diminishing volume of “large” ESs, and a proliferation of “tiny” ESs in recent years (Additional Fig. S5D). The trend was found in nearly all fields of biomedical research, with the most marked downward trend concerning genetic phenomena (Fig. 2). It was also found on nearly all continents (Additional Fig. S5E). ESs from abstracts of reviews showed a modest decrease of ESs (Additional Fig. S6A), but the decrease was not found in subgroups of ESs with 90% or 99% CIs (Additional Fig. S6B). Analysis of full-text PMC articles confirmed the decreasing trend for abstracts and tables (τ value of –0.44 and –0.21, *P* < 0.001) but not for Results sections (τ value = –0.04, *P* = 0.41) (Additional Fig. S6C).

### ESs are becoming more often statistically significant

**Figure 4:**
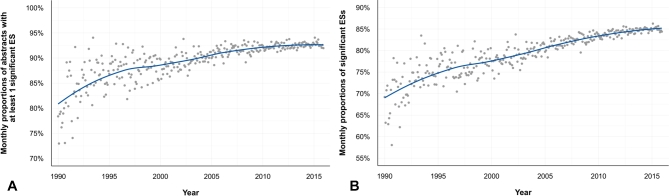
Proportions of statistically significant ESs have increased with time in 247 339 abstracts. (**A**) Scatter plot of the temporal evolution of monthly proportions of abstracts with at least 1 statistically significant ES. There is a monotonic upward trend: τ value = 0.65 (*P* < 0.001). (**B**) Scatter plot of the temporal evolution of the monthly mean of proportions of statistically significant ES per abstract. There is a monotonic upward trend: τ value = 0.77 (*P* < 0.001).

At the same time as ES values have fallen, the proportion of statistically significant ESs has increased. Again, this finding was constant for each outcome considered (i.e., presence of at least 1 statistically significant ES per abstract, or proportion of statistically significant ESs per abstract) (Fig. 4A, B), for both “risk” and “protective” ESs, and whatever their type (OR, RR, HR) or the continent in question (Additional Fig. S7A–D). CIs are now narrower than in the past (Fig. 3C), while limits near 1 are quite stable, even slightly farther from 1 for the upper limits of “protective” ESs: between 1990–1995 and 2010–2015, overall medians of 95% CI limits evolved from 1.23–4.96 to 1.21–3.54 for “risk” values, and from 0.32–0.95 to 0.42–0.91 for “protective” values. There was no evidence of an increasing trend in abstracts of reviews (Additional Fig. S6D), nor in subgroups of ESs with 90% or 99% CIs (Additional Fig. S6E), but the proportion of statistically significant ESs in PMC full-text articles also increased (τ = +0.50, *P* < 0.001 for abstracts and Results sections) (Additional Fig. S6F).

### Factors associated with observed trends

**Figure 5:**
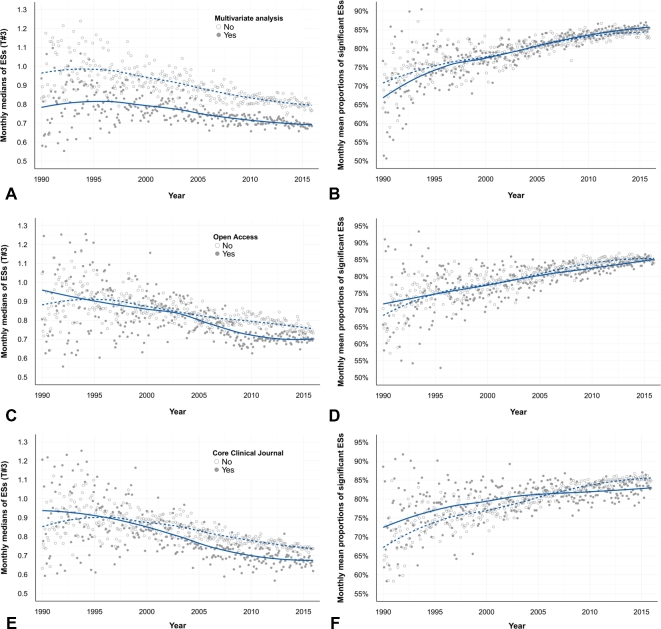
Factors associated with observed trends. Scatter plots of the temporal evolution of monthly medians of ESs (**A, C, E**) or mean proportions of statistically significant ESs per abstract (**B, D, F**), according to presence (yes/no) of the following factors: a multivariate analysis (**A, B**), the Open Access status of the article (**C, D**) or the “Core Clinical Journal” status of the article (**E, F**). The full line represents the temporal trend for abstracts with evidence of the factor, and the dotted line without evidence of the factor. ESs were considered at the abstract level. The outcome was the mean of ES(s) of each abstract (on a linear scale, T#3). (**A**) ESs from abstracts with multivariate analysis were generally lower than values from abstracts without multivariate analysis during the 25 year period (*P* < 0.001, Mann-Whitney test). (**B**) There was no statistical difference between the 2 categories regarding statistical significance during the 25-year period (*P* = 0.59, Mann-Whitney test). Number of abstracts: 136 724 and 110 615 abstracts with and without multivariate analysis, respectively. (**C**) ESs from Open Access abstracts were generally lower than values from non–Open Access abstracts during the 25-year period (*P* < 0.001, Mann-Whitney test). (**D**) There was no statistical difference between the 2 categories regarding statistical significance during the 25-year period (*P* = 0.57, Mann-Whitney test). Number of abstracts: 92 040 Open Access and 155 299 non–Open Access abstracts. (**E**) ESs from CCJ abstracts were generally lower than values from non-CCJ abstracts during the 25-year period (*P* < 0.001, Mann-Whitney test), especially from around the year 2000 onwards. (**F**) There was no difference between the 2 categories regarding statistical significance during the 25-year period (*P* = 0.08, Mann-Whitney test). However, we can see that the curves cross around 2005. When the period between 2005 and 2015 was considered, ESs from CCJ abstracts were less often statistically significant (*P* < 0.001, Mann-Whitney test).

Both decreasing ESs and increasing significance were found in abstracts with evidence of a multivariate analysis, from Open Access (OA) journals and from Core Clinical Journals (CCJ) (Fig. 5). However, we found some evolutions in the general environment of publishing: (1) a growing use of multivariate analyses (Additional Fig. S2E), (2) an increasing appeal for Open Access publication (Additional Fig. S2F), and (3) a smaller proportion of abstracts from Core Clinical Journals (Additional Fig. S2G). These changes could accentuate the observed trends because (1) ESs from abstracts with multivariate analysis were lower than unadjusted ES values (with no difference concerning statistical significance) (Fig. 5A, B), (2) ES values reported in abstracts from OA journals were lower than those from non-OA journals (but with a similar proportion of statistical significance) (Fig. 5C, D), and (3) ESs from CCJ also decreased but, above all, became less often statistically significant than in non-CCJ over time (Fig. 5E, F).

## Discussion

Epidemiology has now reached the paradoxical situation where ESs are decreasing remarkably over time, while these same ESs are becoming more and more often statistically significant. We call this surprising phenomenon the *in silico* effect, by analogy with the evolution of processors (the size of which has decreased as their performance has grown) and because the rise of computer science is, at least indirectly, linked with this general trend (advances in statistical methods and software, availability of huge electronic databases and larger studies, etc.).

The global decrease of ESs could be explained by several inter-related considerations. First, as already pointed out by Taubes in 1995, there could be a true rarefaction over time of undiscovered conspicuous determinants of diseases, such as smoking or alcohol [11]. We showed that this trend could be observed worldwide and in most fields of biomedical research. Second, methodological improvements in biomedical research [12] could also have led to smaller ESs. Most importantly, it is highly probable that larger sample sizes could lead to smaller effect sizes (e.g., through better management of confounders), which are likely to be statistically significant (through an increase in statistical power). Indeed, multivariate analyses are more frequently used as time goes on, which could lead to weaker effects than those obtained with univariate analyses [13]. Third, cultural effects should also be considered. We found that ESs have become smaller in contemporary CCJ. “Modest” ESs (i.e., <RR ∼ 3) are no longer “discredited,” as may have been the case in the past (e.g., by some former editors of Core Clinical Journals) [11], and slight associations have now become the rule [14]. It is now accepted, at least in some fields of research, that most true associations have small effects [15]. Another kind of cultural explanation appears when different geographical areas are examined: the “five eyes” countries (Australia, Canada, New Zealand, the United Kingdom, and the United States—the greatest producers and influencers of biomedical research) [16] and the Scandinavian monarchies (Denmark, Sweden, and Norway) are among the countries reporting the lowest ESs. Interestingly, it has been shown that scientists from these countries may be more cautious when reporting results, as evidenced by their prominent use of words implying uncertainty in their abstracts [17]. This is also consistent with stronger ESs being found in Asian studies than in the European and American literature, e.g., for gene-disease associations [18]. The desire to “compete” with Europe and the United States may be an explanation [14]. Finally, another explanation would be the file drawer effect (i.e., publication bias) [19, 20], which could mask a more pronounced decrease of ESs than the 1 we identified by underestimating the amount of null or negative effects. The increased rejection rates and the increased emphasis on risk factors have encouraged editors and authors to select and present manuscripts with bigger effect sizes and/or significant differences [19].

One should not directly interpret this structural trend at the whole literature level in the same way as has already been described at the level of particular topics in biology [21] or in medical research [22]. Gehr evoked the “fading of reported effectiveness” in randomized controlled trials [23]. Among several explanations [21], the “Proteus phenomenon” [24] has been described to evoke “rapidly alternating extreme research claims and extremely opposite refutations” [25]. Decreasing ESs in a particular topic are likely to lead to a loss of statistical significance [21], as observed in several cumulative meta-analyses [26]. In contrast, while we also measured decreasing ESs, our findings indicated a clear trend toward a growing proportion of statistically significant results over time. This result is consistent with several other trans-disciplinary meta-research results: a trend toward lower *P*-values reported in PubMed abstracts between 1990 and 2015 [27], increasing reporting of significant tiny effects in the literature [28], and an increasing proportion of positive results [29].

Although the decrease in ESs over time does not seem problematic in itself, the growing proportion of statistically significant results is more intriguing and may reflect the “publish or perish” context of scientific research. With a growing population of researchers worldwide [30], all competing to obtain funds, and a probable tendency toward placing greater emphasis on novelty and sensationalism [29], maintaining statistically significant results may have become the way to “compensate” for the decrease of ESs. We also found that the growing proportion of statistically significant results was unaffected by the development of Open Access publishing [31] but could be accentuated by the increasing relative importance of Asian papers.

Among the limitations of this study is the incomplete representation of different possible metrics of ESs [2]: RRs, ORs, and HRs are not the only way to report measures of associations. Although it is mathematically conceivable to standardize other ES metrics (e.g., to convert Cohen's d, Hedges’ g, and correlation coefficient to odds ratio following standard transformations [32], as already done in other meta-research [33]), we could not perform data mining on all existing metrics with sufficient accuracy to guarantee the best measurement quality. However, it is rather unlikely that the *in silico* effect would be specific to particular metrics. We also did not filter out analyses in regard to RR/OR/HR that were expressed per unit of continuous variable, but this limitation should not have any effect on temporal trends. One could argue that the heterogeneity of the data that forms the basis of the analysis makes it impossible to infer the meaning of these trends. ESs reflect the effects of continuous, categorical, or binary measures and include risk factors for diseases, treatment effects of new drugs vs placebo, genetic effects, effects of risk scores, etc. However, considering the biomedical literature as a whole is the only way to assess macro-trends in the way ESs are reported. Given that practical interpretation of ESs has not really changed over time, it is important to identify such trends. Other limitations are related to the data available in XML files of PubMed abstracts, and to the automatic nature of the data mining process: both these considerations prevented us from carrying out in-depth analysis of results in relation to sample sizes, e.g., quality of studies or conflicts of interest.

### Potential implications

In this era of alternative truths and bullying of the press, the public and politicians need a science of epidemiology that is credible and trustworthy. Echoing Taubes [11], it is still important for epidemiology to avoid becoming an “unending source of fear,” with too many studies having too little real impact on public health. The medical and research community should acknowledge forces and constraints that influence the design of studies and the way their results are interpreted, because they have significant impact on health decisions and policies. We suggest that biomedical researchers should be skilled in meta-research in order to take a bird's eye view of science [34]. More than ever, efforts to improve the credibility of biomedical research and limit waste of resources must be continued [35]. This implies important provisions, described by Ioannidis [36], among others, such as the adoption of replication culture, changes in the way statistical methods are designed and used in the reporting and interpretation of results [37], and modifications in the reward system of science [38], to name but a few. From our results, we can add the consideration to be accorded to Core Clinical Journals when making health decisions and policies: the importance of their role both in maintaining quality of research and in filtering articles of clinical or scientific importance seems to be growing. Finally, intensifying transdisciplinarity with the humanities would help epidemiologists to provide research that would be regarded in terms of its “potential uses and misuses in serving and affecting the human condition” [39].

## Methods

We followed a KDD approach. The KDD process is iterative and involves several steps, combining automated methods with human decisions [40]. The following subsections describe all final iterations. The overall process is described in Additional Fig. S1A–C. Algorithms and statistical scripts are explained in the Supplementary Information and are downloadable [41].

### Data mining

Using an iterative process, we developed an algorithm aimed to automatically detect the 3 main types of ESs (OR, RR, HR) in PubMed abstracts. As terminology was poorly standardized, we iteratively refreshed a list of ES terms frequently used in biomedical research, e.g., “RR,” “OR,” “HR,” “relative risk,” “odds ratio,” “hazard ratio,” “aRR,” “aOR,” “aHR,” etc. (Additional Table S1). We also filtered numeric values not likely to be ES values and checked for polysemy of acronyms. The algorithm [41] was tailored to detect the full wording of all medical abbreviations having reported values that could be confused with those of ES terms using the same abbreviation (e.g., “respiratory rate” for RR, “ovulation rate” for OR, “heart rate” for HR) (Additional Table S1).

Each attempt to improve the detection of ESs was tested for diagnostic performance on random samples of 200 abstracts, and iterations were validated if both sensitivity and specificity were improved. At the final iteration, a sensitivity greater than 95% and a specificity of 99.9% (interobserver κ > 0.97) were reached (Supplementary Methods, Additional Table S2, and Supplementary File 1 for performance testing).

The algorithm automatically recognized the type of ES, its value, and the values of upper and lower limits of its CI (Supplementary Methods). Other characteristics of the citation that the ES was drawn from were retrieved: PubMed identifier (PMID), ±PMC identifier (PMCID), month/year of publication, authors’ affiliation country(ies), Medical SubHeadings (MeSH) keywords, detection of a multivariate analysis (yes/no), OA publication (yes/no), publication in a CCJ (yes/no), CI level (i.e., 90%, 95%, or 99%), and type of publication (“review”: yes/no).

Given the small number of abstracts indexed per year [27] before 1990 and the as-yet incomplete indexing of abstracts from 2016, only the 1990–2015 period was considered. This process led to the generation of a comprehensive database of 814 120 ES values (fully available in *Giga*DB [41]).

### Data transformation

By nature, OR/RR/HR values are expressed on a logarithmic scale (between 0 and 1 for “protective” values, and between 1 and +∞ for “risk” values). The logarithmic transformation of these ESs has the useful property of being normally distributed [42], and the absolute value of the ln-transformed ESs provides a standardization of “protective” and “risk” values. Depending on whether ES values were normalized and/or standardized, 4 different transformations were defined (rationale and mathematical explanations in Additional Table S3a).

### Data analysis

#### Outcomes

We defined 3 types of ESs: ORs, RRs, and HRs.

Original ESs values were categorized as:

- “protective” if <1, “risk” if >1, “neutral” if =1;
- “large” [43] when ≤0.2 or ≥5, and “tiny” [28] if between 0.95 and 1.05;
- statistically significant if the CI did not encompass 1.

As multiple ESs are often found within a single abstract, for analyses at the abstract level, ES values were condensed in different ways (Additional Table S3B):

- minimal and maximal ES values per abstract (i.e., the nearest value to 1 and the farthest value from 1, respectively);
- mean of ES values per abstract (after logarithmic transformation);
- magnitude of CIs (minimal, maximal, and mean per abstract after logarithmic transformation);
- presence of at least 1 statistically significant ES value in the abstract (yes/no) and proportion of statistically significant ESs per abstract.

Primary analyses were confined to non-reviews to avoid overrepresentation of some ES values, and to ESs with 95% CI to allow magnitude comparisons of CIs.

#### Analysis plan

An iterative analysis plan was designed for the 3 aims of the study. Specific objectives were listed (Additional Table S4).

#### Statistical analyses

Descriptive analyses involved calculations of frequency distribution, percentages, means, and tabular statistics for the reporting of ESs (both by type of ES and all taken together for readability purposes). The monotonic upward or downward trend of monthly medians of ES values over time was assessed using the Mann-Kendall (MK) test [44]. ES comparisons between classes of binary variables were tested using Mann-Whitney statistics. A Kruskal-Wallis pairwise comparison (using Dunn's test for multiple comparisons) was achieved to compare values across continents. The significance level of statistical tests was set at *P* <0.001. Statistics and graphics for data visualization were produced using R 3.2.3 (Vienna, Austria, 2015; R Project for Statistical Computing, RRID:SCR_001905). A “loess” fitted curve [45] was added to scatterplots in order to visualize temporal trends.

### Knowledge checking [40]

#### Systematic reviews and other types of CI

Complementary analyses on temporal evolution of ESs were conducted on 2 subgroups not included in the primary analyses: ESs detected in citations identified as “review” and ESs with CI at 90% or 99% (Additional Fig. S2H, I).

#### PMC database

As an abstract may not be fully representative of the full-text article, we extended the data mining process to full-text articles; 64 829 citations with a PMCID number were thus selected from the comprehensive database. XML data from corresponding PMC articles (25 868 available articles) were then downloaded, and a similar data-mining strategy was applied to the Results sections: 135 542 values were detected; 589 743 ESs were also detected within tables and analyzed separately [41].

## Availability of source code and requirements

Project name: PubMed ES Detector Source code available at: https://github.com/gigascience/paper-monsarrat2017 & http://dx.doi.org/10.5524/100385.

Operating systems: platform independent

Programming language: Perl

License: GNU GPL v3

## Availability of supporting data and materials

Further supporting data are available in the *GigaScience* repository, GigaDB [41]. The dataset contains the comprehensive database of detected ESs in Pubmed, the database of detected ESs in PubMed Central, and snapshots of the source code of the program that helped to generate these databases. Three specific modules were developed: ES_detector.pm, Load_module.pm and Mesh_detector.pm. The flow diagram of the program can be found in Additional Fig. S1.

## Additional files

Additional information may be found in the Supplementary Information pdf file.

The Supplementary Methods contains additional information about the data mining method, programming algorithm, performance tests of the algorithm, and definition of citation characteristics.

Additional Table 1: check for polysemy of terms related to types of ESs. The algorithm checked for the polysemy of acronyms. Through the MediLexicon online database of pharmaceutical and medical abbreviations (http://www.medilexicon.com/), all potential synonyms were identified by text mining on the entire abstract. All the terms considered are presented below. From regular expressions, some variations were considered to increase the detection of ES acronyms: presence or absence of plural, hyphen, or spaces. The presence of any of these terms in an abstract oriented the data mining process toward a more restrictive procedure in order to minimize the “false positive” rate.

Additional Table 2: Examples of “undetectable” ESs, false-negative ESs, and false-positive ESs.

Additional Table 3: Mathematical transformations and main outcomes.

Additional Table 4: Summary table of the analysis plan.

Additional Table 5: Geographical analysis.

Additional Figure 1: Overview of the “Knowledge Discovery in Databases” (KDD) approach used in this study: the different steps that compose the KDD process, the flowchart of the algorithm for PubMed data mining, and the flow diagram of the selection process for abstracts.

Additional Figure 2: Descriptive analysis of the comprehensive database and descriptive analysis of ESs in abstracts.

Additional Figure 3: Histogram distribution of the effect sizes.

Additional Figure 4: Heatmap of the temporal evolution of proportion of statistically significant ESs per abstract: disparities among fields of research.

Additional Figure 5: Descriptive analysis of ES values in abstracts for protective and risk values, type of ESs, tiny and large effects, and geographical areas.

Additional Figure 6: Descriptive analysis of ES values and significance from reviews, according to confidence intervals, from PMC full texts.

Additional Figure 7: Descriptive analysis of ES significance in abstracts for protective and risk values, type of ESs, tiny and large effects, and geographical areas.

Supplementary File 1 contains additional information about performance testing: kappa, sensitivity, and specificity.

Supplementary References

### Abbreviations

CCJ: Core Clinical Journal; CI: confidence interval; ES: effect size; HR: hazard ratio; KDD: Knowledge Discovery in Databases; MK: Mann-Kendall; NLM: National Library of Medicine; OA: Open Access; OR: odds ratio; PMC: PubMed Central; PMID: PubMed ID; RR: relative risk; XML: eXtensible Markup Language.

### Competing financial interests

The authors declare that they have no competing interests.

### Funding

This work was supported by Toulouse University Hospital (CHU de Toulouse), Toulouse University (Université Paul Sabatier), the Midi-Pyrenees region, the research platform of the Toulouse Dental Faculty (PLTRO), and the French National Research Agency (Agence Nationale de la Recherche—ANR—http://dx.doi.org/10.13039/501100001665) under grant ANR-16-CE18–0019-01.

### Author contributions

P.M. and J.N.V. designed the research, analyzed and interpreted the data, performed the statistical analysis, and drafted the manuscript. P.M. acquired the data and coded the algorithm. J.N.V. supervised the study.

## Supplementary Material

GIGA-D-17-00176_Revision_1.pdf

GIGA-D-17-00176_Revision_2.pdf

GIGA-D-17-00176__Original_Submission.pdf

Response_to_Reviewer_Comments_Original_Submission.pdf

Response_to_Reviewer_Comments_Revision_1.pdf

Reviewer_1_Report_(Original_Submission) -- Shinichi Nakagawa 20 Aug 2017 Reviewed

Reviewer_1_Report_(Revision_1) -- Shinichi Nakagawa 03 Nov 2017 Reviewed

Reviewer_2_Report_(Original_Submission) -- BjÖrn Brembs 13 Sep 2017 Reviewed

Supplemental materials
