# Supplementary material for: The intriguing evolution of effect sizes in biomedical research over time: smaller but more often statistically significant
Source: Gigascience. 2017 Dec 6;7(1):gix121. doi: 10.1093/gigascience/gix121 (PMC5765564; doi:10.1093/gigascience/gix121)
Supplement: GIGA-D-17-00176__Original_Submission.pdf [file gix121_giga-d-17-00176__original_submission.pdf]

GigaScience

The surprising evolution of effect sizes in biomedical research between 1990 and 2015: smaller but more often statistically significant

--Manuscript Draft--

**Manuscript Number:**	GIGA-D-17-00176
**Full Title:**	The surprising evolution of effect sizes in biomedical research between 1990 and 2015: smaller but more often statistically significant
**Article Type:**	Research
**Funding Information:**	Agence Nationale de la Recherche (ANR-16-CE18-0019-01) — Dr Paul Monsarrat
**Abstract:**	Background: In medicine, effect sizes (ESs) allow the effects of independent variables (including risk/protective factors, or treatment interventions) on dependent variables (e.g. health outcomes) to be quantified. Given that many public health decisions and health care policies are based on ESs estimates, it is important to assess how ESs are used in the biomedical literature and to investigate potential trends in their reporting over time.<br><br>Results: Through a big data approach, the text mining process automatically extracted 814120 ESs from 13322754 PubMed abstracts. Eligible ESs were risk ratio, odds ratio and hazard ratio, along with their confidence intervals. Here we show a remarkable decrease of ESs values in PubMed abstracts between 1990 and 2015, concomitantly becoming more often statistically significant. Medians of ESs values have decreased over time for both "risk" and "protective" values. This trend was found in nearly all fields of biomedical research, with the most marked downward tendency in genetics. Over the same period, the proportion of statistically significant ESs has increased regularly: among the abstracts with at least one ES, 74% were statistically significant in 1990-1995, vs. 85% in 2010-2015.<br><br>Conclusions: Whereas decreasing ESs could be an intrinsic evolution in biomedical research, the increase of statistically significant results is more worrying. The main explanation may lie in the "publish or perish" context of scientific research, with the probability of a growing orientation towards sensationalism in research reports. Important provisions must be made to improve the credibility of biomedical research and limit waste of resources.
**Corresponding Author:**	Jean-Noel Vergnes<br><br>FRANCE
**Corresponding Author Secondary Information:**	
**Corresponding Author's Institution:**	
**Corresponding Author's Secondary Institution:**	
**First Author:**	Paul Monsarrat
**First Author Secondary Information:**	
**Order of Authors:**	Paul Monsarrat<br>Jean-Noel Vergnes
**Order of Authors Secondary Information:**	
**Opposed Reviewers:**	
**Additional Information:**	
**Question**	**Response**
Are you submitting this manuscript to a	No

Powered by Editorial Manager® and ProduXion Manager® from Aries Systems Corporation

special series or article collection?	
**Experimental design and statistics**<br><br>Full details of the experimental design and statistical methods used should be given in the Methods section, as detailed in our Minimum Standards Reporting Checklist. Information essential to interpreting the data presented should be made available in the figure legends.<br><br>Have you included all the information requested in your manuscript?	Yes
**Resources**<br><br>A description of all resources used, including antibodies, cell lines, animals and software tools, with enough information to allow them to be uniquely identified, should be included in the Methods section. Authors are strongly encouraged to cite Research Resource Identifiers (RRIDs) for antibodies, model organisms and tools, where possible.<br><br>Have you included the information requested as detailed in our Minimum Standards Reporting Checklist?	Yes
**Availability of data and materials**<br><br>All datasets and code on which the conclusions of the paper rely must be either included in your submission or deposited in publicly available repositories (where available and ethically appropriate), referencing such data using a unique identifier in the references and in the "Availability of Data and Materials" section of your manuscript.<br><br>Have you have met the above requirement as detailed in our Minimum Standards Reporting Checklist?	Yes

Manuscript
Click here to download Manuscript 2017_07_17_Manuscript.docx

The surprising evolution of effect sizes in biomedical research between 1990 and 2015: smaller but more often statistically significant

Running title: Global trends of effect sizes in medical research

Authors

Paul Monsarrat [1], Jean-Noel Vergnes [2*]

Affiliations

1: Paul Monsarrat, DDS, PhD

Paul Sabatier University, Dental Faculty, Department of Anatomical Sciences and Radiology, Toulouse University Hospital, Toulouse, France.
& STROMALab, Université de Toulouse, CNRS ERL 5311, EFS, INP-ENVT, Inserm, UPS, Toulouse, France

Mail address : UFR Odontologie de Toulouse
3, chemin des maraîchers 31062 Toulouse Cedex 9.
E-mail address: paul.monsarrat@univ-tlse3.fr
Telephone number: +33 6 84 47 48 55
Fax number: +33 5 61 25 47 19

2 : Jean-Noel Vergnes, DDS, PhD (Corresponding author*)

Paul Sabatier University, Dental Faculty, Department of Epidemiology and Public Health, Toulouse University Hospital, Toulouse, France.
& Division of Oral Health and Society, Faculty of dentistry, McGill University, Montreal, Quebec, Canada.

Mail address : UFR Odontologie de Toulouse
3, chemin des maraîchers 31062 Toulouse Cedex 9.
E-mail address: jn.vergnes@mcgill.ca
Telephone number: +33 6 98 00 03 14
Fax number: +33 5 61 25 47 19

Number of words: 2860
Number of figures: 5
Number of tables: 0
Number of references: 42

Abstract

Background: In medicine, effect sizes (ESs) allow the effects of independent variables (including risk/protective factors, or treatment interventions) on dependent variables (e.g. health outcomes) to be quantified. Given that many public health decisions and health care policies are based on ESs estimates, it is important to assess how ESs are used in the biomedical literature and to investigate potential trends in their reporting over time.

Results: Through a big data approach, the text mining process automatically extracted 814 120 ESs from 13 322 754 PubMed abstracts. Eligible ESs were risk ratio, odds ratio and hazard ratio, along with their confidence intervals. Here we show a remarkable decrease of ESs values in PubMed abstracts between 1990 and 2015, concomitantly becoming more often statistically significant. Medians of ESs values have decreased over time for both "risk" and "protective" values. This trend was found in nearly all fields of biomedical research, with the most marked downward tendency in genetics. Over the same period, the proportion of statistically significant ESs has increased regularly: among the abstracts with at least one ES, 74% were statistically significant in 1990-1995, vs. 85% in 2010-2015.

Conclusions: Whereas decreasing ESs could be an intrinsic evolution in biomedical research, the increase of statistically significant results is more worrying. The main explanation may lie in the "publish or perish" context of scientific research, with the probability of a growing orientation towards sensationalism in research reports. Important provisions must be made to improve the credibility of biomedical research and limit waste of resources.

Keywords

Meta-research, Effect size, Biomedical research, "Publish or perish", Data mining

Background

Effect sizes (ESs) are useful to describe associations in studies that focus broadly on associations between variables [1]. In medicine, ESs allow the effects of independent variables (including risk/protective factors, or treatment interventions) on dependent variables (e.g. health outcomes) to be quantified. There are many different types of ES [2] but, in human biomedical research, ESs are predominantly derived from risk (or rate) ratios (RR), odds ratios (OR) or hazard ratios (HR) [3]. No longer confined to the early domains of epidemiological research (such as epidemiological oncology [4]), use of these estimates is now benefiting all biomedical research (e.g. environmental epidemiology [5], genetics [6], or interventional research [7]). As there is no straightforward relationship between p-values and strengths of association [2], adequate reporting of ESs is strongly recommended by recent statistical guidelines [8]. Given that many public health decisions and health care policies are based on ESs estimates [9], it is important to assess how ESs are used in the biomedical literature and to investigate potential trends in their reporting over time. Consequently, in this s we aim 1) to describe the global use of ESs in the biomedical literature during the last 25 years, 2) to analyze their temporal evolution in terms of strength and statistical significance, and 3) to identify and discuss factors associated with potential evolutions.

Data Description

PubMed is the most commonly used database of biomedical information [10], and was considered as the primary source. The KDD process led us to add the PubMed Central (PMC) database as an additional source of data, according to the aims and modalities described in the “Knowledge checking” subsection of Methods. All PubMed citations were bulk-downloaded in XML format (2017 release dated 12/13/2016) from the FTP servers of the US National Library of Medicine (NLM). Among the 26 759 399 citations, 16 820 871 (63%) provided an abstract, and were thus considered as preprocessed data (Additional Fig. 1a-c). A data mining process was then run to automatically detect ESs (OR, RR, HR) within PubMed abstracts along with several abstracts characteristics (see details in Methods).

Analyses

Reporting of ESs increased greatly over time

2.1% of PubMed abstracts contained at least one ES. The relative proportions of ES reports increased markedly over time (Additional Fig. 2a). More than half of the ESs were OR, with a trend for RR to be substituted by HR (Additional Fig. 2b). ESs>1 were still largely predominant, despite an increase of abstracts with all ESs<1, or with a mix of ESs>1 and ESs<1 (Additional Fig. 2c and Additional Fig 3).

Geographic and thematic disparities in reporting of ESs

Europe and North America were by far the biggest providers of abstracts with ESs (Fig. 1a), although the number was growing considerably in Asia (Additional Fig. 2d). There were notable disparities in ES values among different geographical areas: ES values were higher in South America, Africa and Asia, and lower in Europe, Oceania and North America (Fig. 1a). ESs were more likely to be significant in regions where they were the highest (Fig. 1b, Additional Table 5). Higher ES values and proportions of significant ESs were found in fields dealing with infectious diseases (Fig. 2, Additional Fig. 4).

ESs values are decreasing over time

A major finding was that ESs were decreasing over time. In Fig. 3a, there is a clear, progressive, evolution between the 1990s and the 2010s, with a massive concentration of ESs nearer to the value 1 at the present time. This result was very robust, since the decrease was observed with all tested outcomes per abstract (i.e. minimal, maximal, mean transformed ES values) (Fig. 3b-c). It also concerned both "risk" and "protective" values (Additional Fig. 5a-b): overall medians of ES values for "risk" decreased from RR~2.50 in 1990-1995 to RR~2.11 in 2010-2015 and those for "protective" values from RR~0.59 to RR~0.63. The decrease was observed for all types of ESs, when analyzed separately (Additional Fig. 5c). It was also consistent with a diminishing volume of "large" ESs, and a proliferation of "tiny" ESs in recent years (Additional Fig. 5d). The trend was found in nearly all fields of biomedical research, with the most marked downward trend concerning genetic phenomena (Fig. 2). It was also found on nearly all continents (Additional Fig. 5e). ESs from abstracts of reviews showed a modest decrease of ESs (Additional Fig. 6a), but the decrease was not found in subgroups of ESs with 90% or 99% CIs (Additional Fig. 6b). Analysis of full-text PMC articles confirmed the decreasing

trend for abstracts and tables (τ value of -0.44 and -0.21, p<.001) but not for "Results" sections (τ value=-0.04, p=0.41) (Additional Fig. 6c).

ESs are becoming more often statistically significant

At the same time as ES values have fallen, the proportion of statistically significant ESs has increased. Again, this finding was constant for each outcome considered (i.e. presence of at least one statistically significant ES per abstract, or proportion of statistically significant ESs per abstract) (Fig. 4a-b), for both "risk" and "protective" ESs, and whatever their type (OR, RR, HR) or continent (Additional Fig. 7a-d). CIs are now narrower than in the past (Fig. 3c), while limits near 1 are quite stable, even slightly farther from 1 for the upper limits of "protective" ESs: between 1990-1995 and 2010-2015, overall medians of 95%CI limits evolved from [1.23-4.96] to [1.21-3.54] for "risk" values, and from [0.32-0.95] to [0.42-0.91] for "protective" values. There was no evidence of an increasing trend in abstracts of reviews (Additional Fig. 6d) nor in subgroups of ESs with 90% or 99% CIs (Additional Fig. 6e), but proportion of statistically significant ESs in PMC full-text articles also increased (τ=+0.50, p<.001 for abstracts and "Results" sections) (Additional Fig. 6f).

Factors associated with observed trends

Both decreasing ESs and increasing significance were found in multivariate abstracts, Open Access (OA) abstracts and Core Clinical Journal (CCJ) abstracts (Fig. 5). However, we found some evolutions in the general environment of publishing: 1) a growing use of multivariate analyses (Additional Fig. 2e), 2) an increasing appeal for open access publication (Additional Fig. 2f), and 3) a less quantitative influence of Core Clinical Journals (Additional Fig. 2g). These changes could accentuate the observed trends since, 1) ESs from abstracts with multivariate analysis were lower than unadjusted ES values (with no difference concerning statistical significance) (Fig. 5a-b), 2) ES values reported in abstracts from OA journals were lower than those from non-OA journals (but with a similar proportion of statistical significance (Fig. 5c-d)), and 3) ESs from CCJ also decreased but, above all, became less often statistically significant than in non-CCJ over time (Fig. 5e-f).

Discussion

Epidemiology has now reached the paradoxical situation where ESs are decreasing remarkably over time, while these same ESs are becoming more and more often statistically significant. We call this surprising

phenomenon the *in-silico effect,* by analogy with the evolution of processors (the size of which has decreased as their performance has grown), and because the rise of computer science is, at least indirectly, linked with this general trend (advances in statistical methods and software, availability of big electronic databases and larger studies, etc.).

The global decrease of ESs could be explained by several inter-related considerations. First, as already pointed out by Taubes in 1995, there could be a true rarefaction over time of undiscovered conspicuous determinants of diseases, such as smoking or alcohol [11]. We showed that this trend could be observed worldwide and in most fields of biomedical research. Second, methodological improvements in biomedical research [12] could also have led to smaller ESs. In particular, multivariate analyses are more frequently used as time goes on, which could indeed lead to weaker effects than those obtained with univariate analyses [13]. Third, cultural effects should also be considered. We found ESs have become smaller in contemporary CCJ. “Modest” ESs (i.e. $<RR\sim3$) are no longer “discredited” as may have been the case in the past (for example, by some former editors of Core Clinical Journals [11]), and slight associations have now become the rule [14]. It is now accepted, at least in some fields of research, that most true associations have small effects [15]. Another kind of cultural explanation appears when different geographical areas are examined: the “Five eyes” countries (Australia, Canada, New Zealand, the United Kingdom and the United States – the greatest producers and influencers of biomedical research [16]) and the Scandinavian monarchies (Denmark, Sweden and Norway) are among the countries reporting the lowest ESs. Interestingly, it has been shown that scientists from these countries may be more cautious when reporting results, as evidenced by their prominent use of words implying uncertainty in their abstracts [17]. This is also consistent with stronger ESs being found in Asian studies than in the European and American literature, e.g. for gene-disease associations [18]. The desire to “compete” with Europe and the USA may be an explanation [14].

One should not directly interpret this structural trend at the whole literature level as has already been described at the level of particular topics in biology [19] or in medical research [20]. Gehr evoked the “fading of reported effectiveness” in randomized controlled trials [21]. Among several explanations [19], the “Proteus phenomenon” [22] has been described to evoke the “rapidly alternating extreme research claims and extremely opposite refutations” [23]. Decreasing ESs in a particular topic are likely to lead to a loss of statistical significance [19], as observed in several cumulative meta-analyses [24]. In contrast, while we also

measured decreasing ESs, our findings indicated a clear trend toward a growing proportion of statistically significant results over time. This result is consistent with several other trans-disciplinary meta-research results: a trend toward lower p-values reported in PubMed abstracts between 1990 and 2015[25], increasing reporting of significant tiny effects in the literature [26], and an increasing proportion of positive results [27].

Although the decrease in ESs over time does not seem problematic in itself, the growing proportion of statistically significant results could be more worrying. One possible explanation lies in the "publish or perish" context of scientific research. With a growing population of researchers worldwide [28], all competing to obtain funds, and a probable tendency toward placing greater emphasis on novelty and sensationalism [27], maintaining statistically significant results may have become the way to "compensate" for the decrease of ESs. We also found that the growing proportion of statistically significant results was unaffected by the development of open access publishing [29] but could be accentuated by the increasing relative importance of Asian papers.

Among the limitations of this study is the incomplete representation of different types of ESs [2]. Although it is mathematically conceivable to standardize different ESs (e.g. to convert Cohen's d, Hedges' g, and correlation coefficient to odds ratio following standard transformations [30], as already done in other meta-research [31]), we could not perform data mining on all types of ESs with sufficient accuracy to guarantee the best measurement quality. However, it is rather unlikely that the *in-silico* effect would be specific to particular types of ESs. We also did not filter out analyses in regard to RR/OR/HR that were expressed per unit of continuous variable, but this limitation should not have any effect on temporal trends. One could argue that the heterogeneity of the data that forms the basis of the analysis makes it impossible to infer the meaning of these trends. Indeed, ESs reflect the effects of continuous, categorical or binary measures and include risk factors for diseases, treatment effects of new drugs to placebo, genetic effects, effects of risk scores, etc. However, considering the biomedical literature as a whole is the only way to assess macro-trends in the way ESs are reported. Given that practical interpretation of ESs has not changed over time, it is thus important to identify such trends. Other limitations are related to the available data in XML files of PubMed abstracts, and to the automatic nature of the data mining process: both these considerations prevented us from carrying out in-depth analysis of results in relation to sample sizes, quality of studies or conflicts of interest, for example.

Potential implications

In this era of alternative truths and bullying of the press, the public and politicians need a science of epidemiology that is credible and trustworthy. Echoing Taubes, it is still important for epidemiology to avoid becoming an "unending source of fear", with too many studies having too little real impact on public health. The medical and research community should acknowledge forces and constraints that influence results and their interpretation, since they have significant impact on health decisions and policies. We suggest that biomedical researchers should be skilled in meta-research in order to take "a bird's eye view of science" [32]. More than ever, efforts to improve the credibility of biomedical research and limit waste of resources must be continued [33]. This implies important provisions, described by Ioannidis [34] among others, such as the adoption of replication culture, changes in the way statistical methods are designed and used in the reporting and interpretation of results [35], and modifications in the reward system of science [36], to name but a few. From our results, we can add the consideration to be accorded to Core Clinical Journals when making health decisions and policies: the importance of their role both in maintaining quality of research and in filtering articles of clinical or scientific importance seems to be growing. Finally, intensifying transdisciplinarity with the humanities would help epidemiologists to provide research that would be regarded in terms of its "potential uses and misuses in serving and affecting the human condition" [37].

Methods

We followed a "Knowledge Discovery in Databases" (KDD) approach. The KDD process is iterative and involves several steps, combining automated methods with human decisions [38]. The following subsections describe all final iterations. The overall process is described in Additional Fig. 1a-c. Algorithms and statistical scripts are explained and downloadable in Supplementary Information.

Data mining

Using an iterative process, we developed an algorithm aimed to automatically detect ESs (OR, RR, HR) in PubMed abstracts. As terminology was poorly standardized, we iteratively refreshed a list of ES terms frequently used in biomedical research, e.g. "RR", "OR", "HR", "relative risk", "odds ratio", "hazard ratio", "aRR", "aOR", "aHR", etc. (Additional Table 1). We also filtered numeric values not likely to be ES values and checked for polysemy of acronyms. The algorithm (Supplementary Datasets) was tailored to detect the

full wording of all medical abbreviations having reported values that could be confused with those of ES terms using the same abbreviation (e.g. "Respiratory Rate" for RR, "Ovulation Rate" for OR, "Heart Rate" for HR) (Additional Table 1).

Each attempt to improve the detection of ESs was tested for diagnostic performance on random samples of 200 abstracts, and iterations were validated if both sensitivity and specificity were improved. At the final iteration, a sensitivity superior to 95% and a specificity of 99.9% (inter-observer κ>0.97) were reached (Supplementary Methods, Additional Table 2 and Datasets for performance testing).

The algorithm automatically recognized the type of ES, its value, and the values of upper and lower limits of its confidence interval (CI) (Supplementary Methods). Other characteristics of the citation that the ES was drawn from were retrieved: PubMed identifier (PMID), ±PMC identifier (PMCID), month/year of publication, authors' affiliation country(ies), Medical SubHeadings (MeSH) keywords, detection of a multivariate analysis (yes/no), OA publication (yes/no), publication in a CCJ (yes/no), CI level (i.e. 90%, 95% or 99%), and type of publication ("Review": yes/no).

Given the small number of abstracts indexed per year[25] before 1990, and the as yet incomplete indexing of abstracts from 2016, only the 1990-2015 period was considered. This process led to the generation of a comprehensive database of 814 120 ES values (fully available in Supplementary Datasets).

Data transformation

By nature, OR/RR/HR values are expressed on a logarithmic scale (between 0 and 1 for "protective" values, and between 1 and +∞ for "risk" values). The logarithmic transformation of these ESs has the useful property of being normally distributed [39], and the absolute value of the ln-transformed ESs provides a standardization of "protective" and "risk" values. Depending on whether ES values were normalized and/or standardized, four different transformations were defined (rationale and mathematical explanations in Additional Table 3a).

Data analysis

Outcomes

Original ESs values were categorized as:

- "Protective" if <1, "risk" if >1, "neutral" if =1,

- "Large" [40] when ≤ 0.2 or ≥ 5, and "tiny" [26] if between 0.95 and 1.05,

- Statistically significant if the CI did not encompass 1.

As multiple ESs are often found within a single abstract, for analyses at the abstract level, ES values were condensed in different ways (Additional Table 3b):

- Minimal and maximal ES values per abstract (i.e. the nearest value to 1 and the farthest value from 1, respectively),
- Mean of ES values per abstract (after logarithmic transformation),
- Magnitude of CIs (minimal, maximal and mean per abstract after logarithmic transformation),
- Presence of at least one statistically significant ES value in the abstract (yes/no), and proportion of statistically significant ESs per abstract.

Primary analyses were confined to non-reviews to avoid overrepresentation of some ES values, and to ESs with 95%CI to allow magnitude comparisons of CIs.

Analysis plan

An iterative analysis plan was designed for the three aims of the study. Specific objectives were listed (Additional Table 4).

Statistical analyses

Descriptive analyses involved calculations of frequency distribution, percentages, means and tabular statistics for the reporting of ESs. The monotonic upward or downward trend of ES values over time was assessed using the Mann-Kendall (MK) test [41]. ES comparisons between classes of binary variables were tested using Mann-Whitney statistics. A Kruskal-Wallis pairwise comparison (using Dunn's test for multiple comparisons) was achieved to compare values across continents. The significance level of statistical tests was set at $p<.001$. Statistics and graphics for data visualization were produced using R 3.2.3 (Vienna, Austria, 2015). A “loess” fitted curve [42] was added to scatterplots in order to visualize temporal trends.

Knowledge checking[38]

Systematic reviews and other types of CI

Complementary analyses on temporal evolution of ESs were conducted on two subgroups not included in the primary analyses: ESs detected in citations identified as “Review” and ESs with CI at 90% or 99% (Additional Fig. 2h and i).

PMC database

As an abstract may not be fully representative of the full-text article, we extended the data-mining process to full-text articles. 64 829 citations with a PMCID number were thus selected from the comprehensive database. XML data from corresponding PMC articles (25 868 available articles) were then downloaded and a similar data-mining strategy was applied to the Result sections: 135 542 values were detected. 589 743 ESs were also detected within tables, and analyzed separately (Supplementary Databases).

Availability of source code and requirements

Project name: PubMed ES Detector

Source code available at:

Operating system(s): e.g. Platform independent

Programming language: e.g. Perl

License : GNU GPL v3

Availability of supporting data and materials

The data sets supporting the results of this article are available in the figshare repository, https://figshare.com/s/aa8922ac1a26669cbd09.

Additional information may be found into the Supplementary Information pdf file:

Supplementary Methods: It contains additional information about data mining method, programming algorithm, performance tests of the algorithm and definition of citation characteristics.

Supplementary Tables:

Additional Table 1: Check for polysemy of terms related to types of ESs. The algorithm checked for the polysemy of acronyms. Through the MediLexicon online database of pharmaceutical and medical abbreviations (http://www.medilexicon.com/), all potential synonyms were identified by text mining on the entire abstract. All the terms considered are presented below. From regular expressions, some variations were considered to increase the detection of ES acronyms: presence or absence of plural, hyphen or spaces. The presence of any of these terms in an abstract oriented the data mining process towards a more restrictive procedure, in order to minimize the “False positive” rate (see Supplementary Datasets).

Additional Table 2: Examples of “undetectable” ESs, false negative ESs and false positive ESs.

Additional Table 3: Mathematical transformations and main outcomes.

Additional Table 4: Summary table of the analysis plan.

Additional Table 5: Geographical analysis.

<u>Supplementary Figures:</u>

Additional Fig. 1: Overview of the "Knowledge Discovery in Databases" (KDD) approach used in this study: the different steps that composes the KDD process, the flowchart of the algorithm for PubMed data mining changer and the flow diagram of the selection process for abstracts.

Additional Fig. 2: Descriptive analysis of the comprehensive database and descriptive analysis of ESs in abstracts.

Additional Fig. 3: Histogram distribution of the effect sizes.

Additional Fig. 4: Heatmap of the temporal evolution of proportion of statistically significant ESs per abstract: disparities among fields of research.

Additional Fig. 5: Descriptive analysis of ESs values in abstracts for protective and risk values, type of ESs, tiny and large effects and geographical areas.

Additional Fig. 6: Descriptive analysis of ESs values and significance from Reviews, according to confidence intervals, from PMC full-texts.

Additional Fig. 7: Descriptive analysis of ESs significance in abstracts for protective and risk values, type of ESs, tiny and large effects and geographical areas.

<u>Supplementary Data:</u> It contains additional information about source code of the program and R Script for data analysis and visualization, performance testing: kappa, sensitivity and specificity and comprehensive database.

<u>Supplementary References</u>

Declarations

List of abbreviations

CCJ: Core Clinical Journal

CI: Confidence Interval

ES: Effect Size

HR: Hazard Ratio

KDD: Knowledge Discovery in Databases

MK: Mann-Kendall

NLM: National Library of Medicine

OA: Open Access

OR: Odds Ratio

PMC: PubMed Central

PMCID: PMC ID

PMID: PubMed ID

RR: Relative Risk

XML: eXtensible Markup Language

Consent for publication

Not applicable

Competing financial interests

The authors declare that they have no competing interests.

Funding

This work was supported by Toulouse University Hospital (CHU de Toulouse), by Toulouse University (Université Paul Sabatier), the Midi-Pyrenees region, the research platform of the Toulouse Dental Faculty (PLTRO) and by the French National Research Agency (Agence Nationale de la Recherche - ANR - http://dx.doi.org/10.13039/501100001665) under grant ANR-16-CE18-0019-01.

Authors' contributions

P.M. and J.N.V designed the research, analyzed and interpreted the data, performed the statistical analysis and drafted the manuscript. P.M. acquired the data and coded the algorithm. J.N.V. supervised the study.

Acknowledgments

The authors thank Ms Susan Becker for her assistance with English language editing.

References

1. Rosenthal JA. Qualitative descriptors of strength of association and effect size. J. Soc. Serv. Res. 1996;21:37–59.

2. Durlak JA. How to select, calculate, and interpret effect sizes. J. Pediatr. Psychol. 2009;34:917–28.

3. Anglemyer A, Horvath HT, Bero L. Healthcare outcomes assessed with observational study designs compared with those assessed in randomized trials. Cochrane Database Syst. Rev. 2014;MR000034.

4. Schachter J, Hill EC, King EB, Heilbron DC, Ray RM, Margolis AJ, et al. Chlamydia trachomatis and cervical neoplasia. JAMA. 1982;248:2134–8.

5. National Research Council (US) Committee on Environmental Epidemiology, National Research Council (US) Commission on Life Sciences. Environmental-epidemiology studies: their design and conduct [Internet]. National Academies Press (US); 1997 [cited 2016 Sep 29]. Available from: https://www.ncbi.nlm.nih.gov/books/NBK233644/

6. Khoury MJ, Beaty TH, Cohen BH. Fundamentals of Genetic Epidemiology. Oxford University Press; 1993.

7. Crowther MA, Ginsberg J, Schünemann H, Meyer RM, Lottenberg R. Evidence-Based Hematology. John Wiley & Sons; 2009.

8. Lang T, Altman D. Basic Statistical Reporting for Articles Published in Biomedical Journals: The "Statistical Analyses and Methods in the Published Literature" or The SAMPL Guidelines". Science Editors' Handbook, European Association of Science Editors; 2013.

9. Committee on Decision Making Under Uncertainty, Board on Population Health and Public Health Practice, Institute of Medicine. Environmental Decisions in the Face of Uncertainty [Internet]. Washington (DC): National Academies Press (US); 2013 [cited 2016 Oct 3]. Available from: http://www.ncbi.nlm.nih.gov/books/NBK200848/

10. Falagas ME, Giannopoulou KP, Issaris EA, Spanos A. World databases of summaries of articles in the biomedical fields. Arch. Intern. Med. 2007;167:1204–6.

11. Taubes G. Epidemiology faces its limits. Science. 1995;269:164–9.

12. Reveiz L, Chapman E, Asial S, Munoz S, Bonfill X, Alonso-Coello P. Risk of bias of randomized trials over time. J. Clin. Epidemiol. 2015;68:1036–45.

13. Serghiou S, Patel CJ, Tan YY, Koay P, Ioannidis JPA. Field-wide meta-analyses of observational associations can map selective availability of risk factors and the impact of model specifications. J. Clin. Epidemiol. 2016;71:58–67.

14. Ioannidis JPA. Exposure-wide epidemiology: revisiting Bradford Hill. Stat. Med. 2016;35:1749–62.

15. Khoury MJ, Little J, Gwinn M, Ioannidis JPA. On the synthesis and interpretation of consistent but weak gene-disease associations in the era of genome-wide association studies. Int. J. Epidemiol. 2007;36:439–45.

16. Xu Q, Boggio A, Ballabeni A. Countries' biomedical publications and attraction scores. F1000Research [Internet]. 2014 [cited 2016 Oct 21]; Available from: http://f1000research.com/articles/3-292/v1

17. Netzel R, Perez-Iratxeta C, Bork P, Andrade MA. The way we write. EMBO Rep. 2003;4:446–51.

18. Pan Z, Trikalinos TA, Kavvoura FK, Lau J, Ioannidis JPA. Local literature bias in genetic epidemiology: an empirical evaluation of the Chinese literature. PLoS Med. 2005;2:e334.

19. Koricheva J, Jennions M, Lau J. Temporal Trends in Effect Sizes: Causes, Detection, and Implications [Internet]. Princeton University Press; 2013 [cited 2016 Dec 23]. Available from: https://openresearch-repository.anu.edu.au/handle/1885/65531

20. Ioannidis JPA, Lau J. Evolution of treatment effects over time: Empirical insight from recursive cumulative metaanalyses. Proc. Natl. Acad. Sci. 2001;98:831–6.

21. Gehr BT, Weiss C, Porzsolt F. The fading of reported effectiveness. A meta-analysis of randomised controlled trials. BMC Med. Res. Methodol. 2006;6:25.

22. Ioannidis JPA, Trikalinos TA. Early extreme contradictory estimates may appear in published research: the Proteus phenomenon in molecular genetics research and randomized trials. J. Clin. Epidemiol. 2005;58:543–9.

23. Ioannidis JPA. Why most published research findings are false. PLoS Med. [Internet]. 2005 [cited 2016 May 5];2. Available from: http://www.ncbi.nlm.nih.gov/pmc/articles/PMC1182327/

24. Trikalinos TA, Churchill R, Ferri M, Leucht S, Tuunainen A, Wahlbeck K, et al. Effect sizes in cumulative meta-analyses of mental health randomized trials evolved over time. J. Clin. Epidemiol. 2004;57:1124–30.

25. Chavalarias D, Wallach J, Li A, Ioannidis JA. Evolution of reporting p values in the biomedical literature, 1990-2015. JAMA. 2016;315:1141–8.

26. Siontis GCM, Ioannidis JPA. Risk factors and interventions with statistically significant tiny effects. Int. J. Epidemiol. 2011;40:1292–307.

27. Fanelli D. Negative results are disappearing from most disciplines and countries. Scientometrics. 2012;90:891–904.

28. Pautasso M. Publication growth in biological sub-fields: patterns, predictability and sustainability. Sustainability. 2012;4:3234–47.

29. Kurata K, Morioka T, Yokoi K, Matsubayashi M. Remarkable growth of open access in the biomedical field: analysis of PubMed articles from 2006 to 2010. PLoS ONE [Internet]. 2013 [cited 2016 Feb 17];8. Available from: http://www.ncbi.nlm.nih.gov/pmc/articles/PMC3641021/

30. Lipsey MW, Wilson D. Practical Meta-Analysis. 1 edition. Thousand Oaks, Calif: SAGE Publications, Inc; 2000.

31. Fanelli D, Ioannidis JPA. US studies may overestimate effect sizes in softer research. Proc. Natl. Acad. Sci. U. S. A. 2013;110:15031–6.

32. Ioannidis JPA, Fanelli D, Dunne DD, Goodman SN. Meta-research: evaluation and improvement of research methods and practices. PLoS Biol. [Internet]. 2015 [cited 2017 Jan 5];13. Available from: http://www.ncbi.nlm.nih.gov/pmc/articles/PMC4592065/

33. Macleod MR, Michie S, Roberts I, Dirnagl U, Chalmers I, Ioannidis JPA, et al. Biomedical research: increasing value, reducing waste. Lancet Lond. Engl. 2014;383:101–4.

34. Ioannidis JPA. How to make more published research true. PLoS Med. 2014;11:e1001747.

35. Sterne JAC, Smith GD. Sifting the evidence—what's wrong with significance tests? BMJ. 2001;322:226–31.

36. Ioannidis JPA, Khoury MJ. Assessing value in biomedical research. JAMA. 2014;312:483–4.

37. Giordano J. Quo vadis? Philosophy, Ethics, and Humanities in Medicine - preserving the humanistic character of medicine in a biotechnological future. Philos. Ethics Humanit. Med. 2009;4:12.

38. Fayyad U, Piatetsky-Shapiro G, Smyth P. From data mining to knowledge discovery in databases. AI Mag. 1996;17:37–54.

39. Bland JM, Altman DG. The odds ratio. BMJ. 2000;320:1468.

40. Pereira TV, Horwitz RI, Ioannidis JA. Empirical evaluation of very large treatment effects of medical interventions. JAMA. 2012;308:1676–84.

41. Esterby SR. Review of methods for the detection and estimation of trends with emphasis on water quality applications. Hydrol. Process. 1996;10:127–49.

42. Jacoby WG. Loess: a nonparametric, graphical tool for depicting relationships between variables. Elect. Stud. 2000;19:577–613.

Figures

Fig. 1: ESs are subject to geographic disparities

(a) Treemap of medians of ESs (T#3) by continent. All detected ESs were considered for the comparisons between continents. For each continent, the size of the rectangle is proportional to the absolute number of abstracts with at least one detected ES. ESs from abstracts with cross-continental affiliations (5.2% of abstracts) were counted in each continent concerned. The grayscale indicates median values of ESs (on a linear scale, T#3) by continent: lighter gray corresponds to lower ESs values, and darker gray to higher ESs values. In rectangles, different letters correspond to statistically different ESs (Kruskal-Wallis pairwise comparisons test). Europe and North America were by far the biggest providers of abstracts with ESs. ESs values were higher in abstracts from South America, Africa and Asia, and lower in abstracts from Europe, Oceania and North America. Number of abstracts: 238954.

(b) Histogram of mean and standard error of proportions of statistically significant ESs per abstract, according to continent. Beneath the bars, different letters correspond to statistically different values (Kruskal-Wallis pairwise comparisons test). ESs were more likely to be statistically significant in abstracts from South America, Africa and Asia.

Fig. 2: Heatmap of the temporal evolution of median ESs (T#3) by research field.

ESs were considered at the abstract level, using the mean of ES(s) per abstract (on a linear scale, T#3). Abstracts were linked to specific research field(s) according to their (MeSH) keywords, so a single abstract could be linked to multiple fields of research (overall ratio: 801839/229581=3.49). Research fields (at the right of the figure) were defined from two main branches of the MeSH Tree (US NLM): [C] “Diseases” and [G] “Phenomena and Processes”. Numbers in brackets are the total number of abstracts with at least one detected ES during the 25-year period in a specific research field. Three branches (out of 43) with fewer than 1000 abstracts with at least one ES were eliminated. Trends were calculated at the monthly level but are represented in the graph at the yearly level for readability. The grayscale indicates yearly median values of ESs (T#3): lighter gray corresponds to lower ES values, and darker gray to higher ES values. On the left, research fields are grouped using a hierarchical cluster analysis and represented as a dendrogram: higher ESs are found in

fields dealing with infectious diseases (e.g. Microbiological Phenomena, Virus Diseases, Bacterial Infections and Mycoses, see top of the figure). The color scale indicates the τ value of the evolution of monthly medians of ESs for each research field. Blank rectangles mean non-significant trends. Colored rectangles are red (not blue), with variable intensity, indicating a significant monotonic downward trend of ESs in nearly all research fields. The most marked decrease is observed for Genetic Phenomena (τ=-0.52, p<.001). Number of abstracts: 229581.

Fig. 3 : ESs are decreasing over time

(a) Heatmap of the temporal evolution of ESs (i.e. Odds Ratio, Relative Risk or Hazard Ratio), on their original log scale (T#1, see detailed in Supplementary Methods). All detected ESs (N=690196) are considered. ES <1 were transformed according to the T#1 transformation (inverse transformation, Table S3a). ES>1 were not transformed. The vertical axis corresponds to a logarithmic scale ranging from 1 to 100, with 25 regular cut-off values (ESs that were >100, corresponding to 0.16% of all detected ESs, are not reported on the graph). The color scale indicates the monthly relative proportion of ESs in each interval: cold colors correspond to lower proportions and hot colors to higher. We can see a trend toward a massive concentration of ES values near to 1 at present. The black dots represent the overall relative proportion of ESs, by year and by interval. We can see that the lowest ESs of the more recent abstracts are the most numerous ESs overall.

(b) Scatter plot of the temporal evolution of monthly medians of ESs, on a linear scale (T#3).
ESs were considered at the abstract level (N=247339). Three different outcomes were considered: minimal, maximal, and mean of ES(s) of each abstract. The three temporal evolutions are decreasing, with τ values of -0.64 (p<.001), -0.59 (p<.001) and -0.63 (p<.001), respectively.

(c) Scatter plot of the temporal evolution of monthly medians of confidence interval (CI) magnitudes, on a linear scale (T#3). CI magnitudes were considered at the abstract level (N=247339). Three different outcomes are considered: minimal, maximal and mean of CI magnitude(s) of each abstract. The three temporal evolutions are decreasing, with τ values of -0.76 (p<.001), -0.67 (p<.001) and -0.72 (p<.001), respectively.

Fig. 4: Proportions of statistically significant ESs has increased with time

247339 abstracts.

(a) Scatter plot of the temporal evolution of monthly proportions of abstracts with at least one statistically significant ES. There is a monotonic upward trend: τ value = 0.65 (p<.001).

(b) Scatter plot of the temporal evolution of monthly mean of proportions of statistically significant ES per abstract. There is a monotonic upward trend: τ value = 0.77 (p<.001).

Fig. 5: Factors associated with observed trends

Scatter plots of the temporal evolution of monthly medians of ESs (A, C, E) or mean proportions of statistically significant ESs per abstract (B, D, F), according to presence (yes/no) of the following factors: a multivariate analysis (A, B), the open access status of the article (C, D) or the "Core Clinical Journal" status of the article (E, F). The full line represents the temporal trend for abstracts with evidence of the factor, and the dotted line without evidence of the factor. ESs were considered at the abstract level. The outcome was the mean of ES(s) of each abstract (on a linear scale, T#3).

(a) ESs from abstracts with multivariate analysis were generally lower than values from abstracts without multivariate analysis during the 25 year period (p<.001, Mann-Whitney test). **(b)** There was no statistical difference between the two categories regarding statistical significance during the 25 year period (p=.59, Mann-Whitney test). Number of abstracts: 136724 and 110615 abstracts with and without multivariate analysis, respectively.

(c) ESs from open access abstracts were generally lower than values from non-open access abstracts during the 25 year period (p<.001, Mann-Whitney test). **(d)** There was no statistical difference between the two categories regarding statistical significance during the 25 year period (p = .57, Mann-Whitney test). Number of abstracts: 92040 open access and 155299 non-open access abstracts.

(e) ESs from CCJ abstracts were generally lower than values from non-CCJ abstracts during the 25 year period (p<.001, Mann-Whitney test), especially from around the year 2000 onwards. **(f)** There was no difference between the two categories regarding statistical significance during the 25 year period (p = .08, Mann-Whitney test). However, we can see that the curves cross around 2005. When the period between 2005 and 2015 was considered, ESs from CCJ abstracts were less often statistically significant (p<.001, Mann-Whitney test).

Figure 1

Click here to download Figure Figure 1.tif

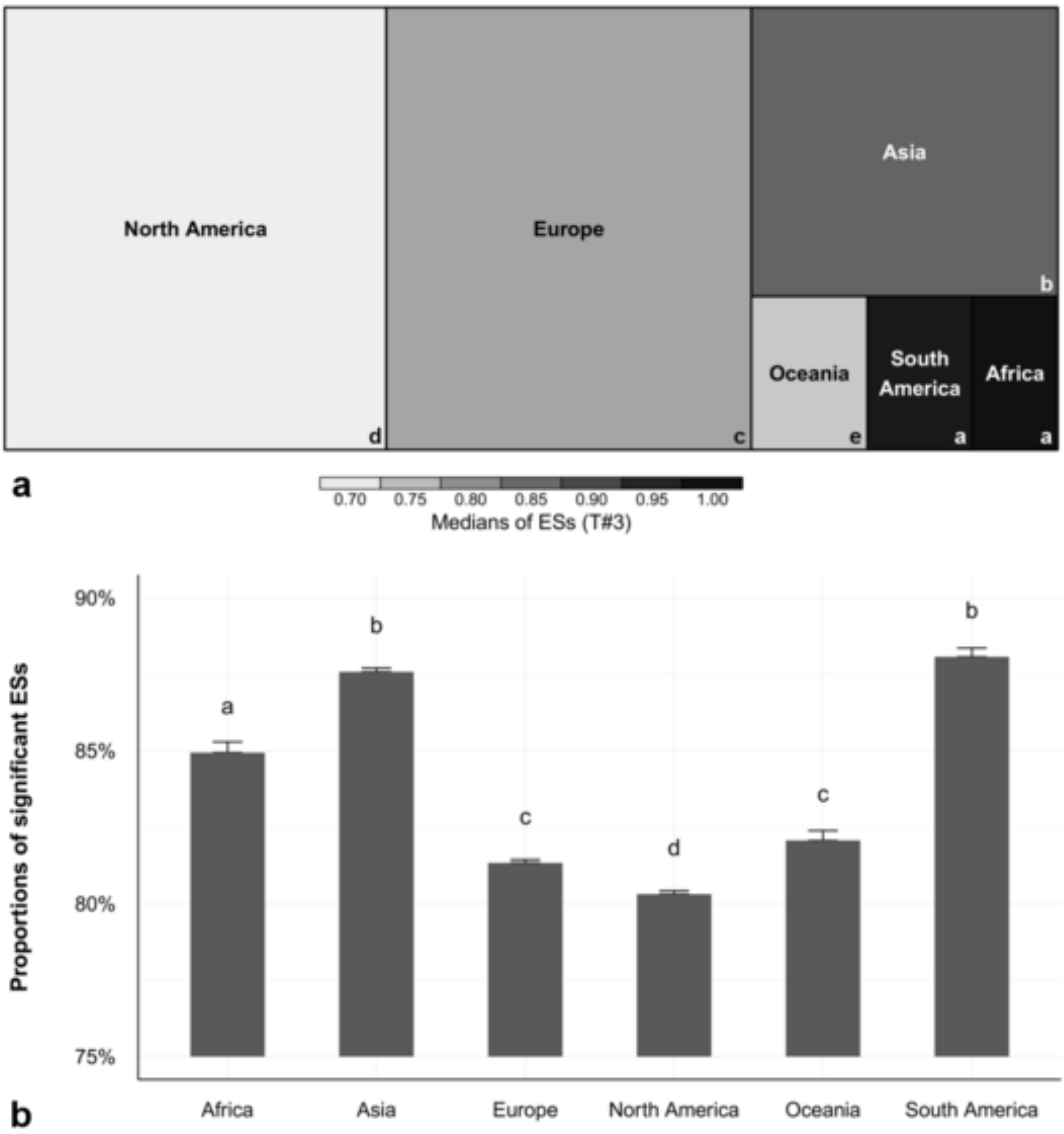

Figure 2

Click here to download Figure Figure 2.tif

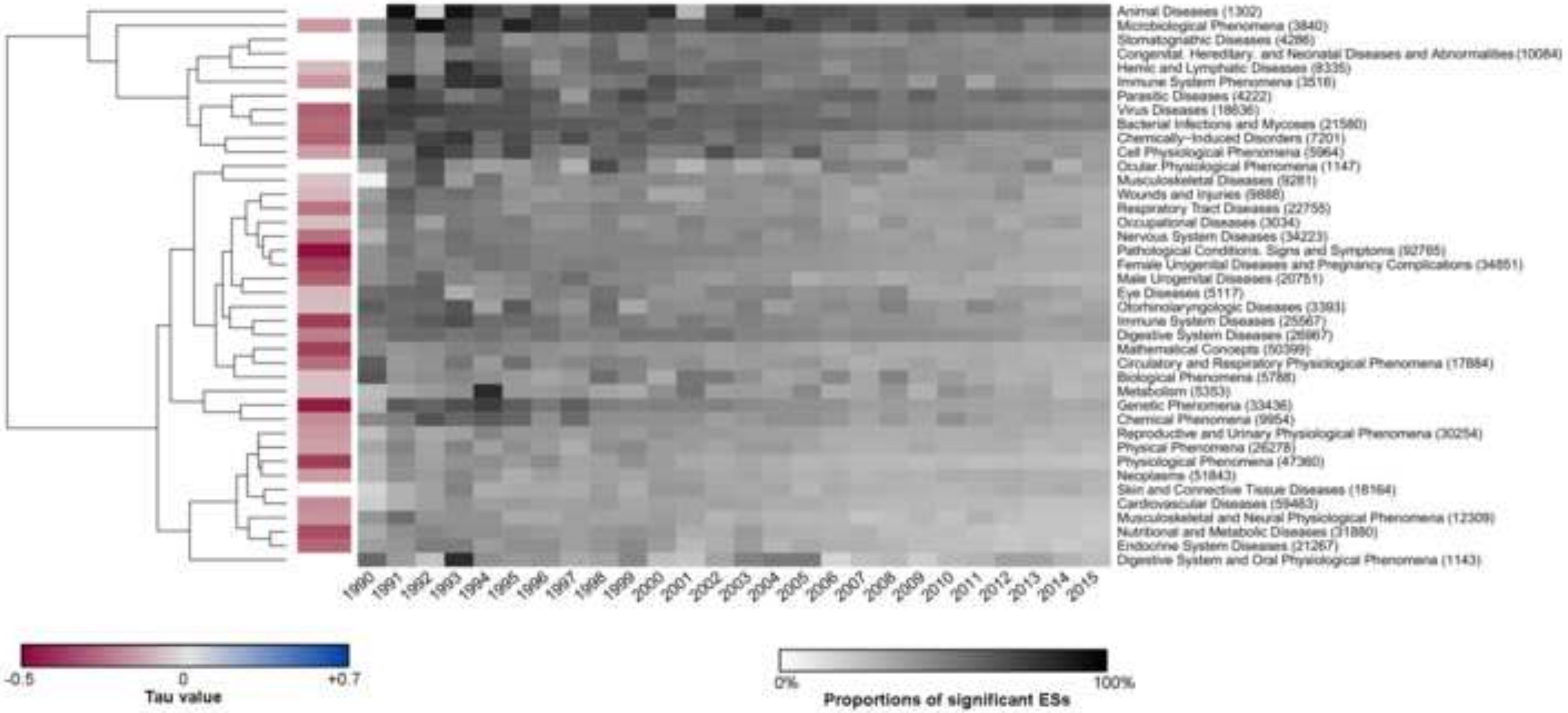

Figure 3

Click here to download Figure Figure 3.tif

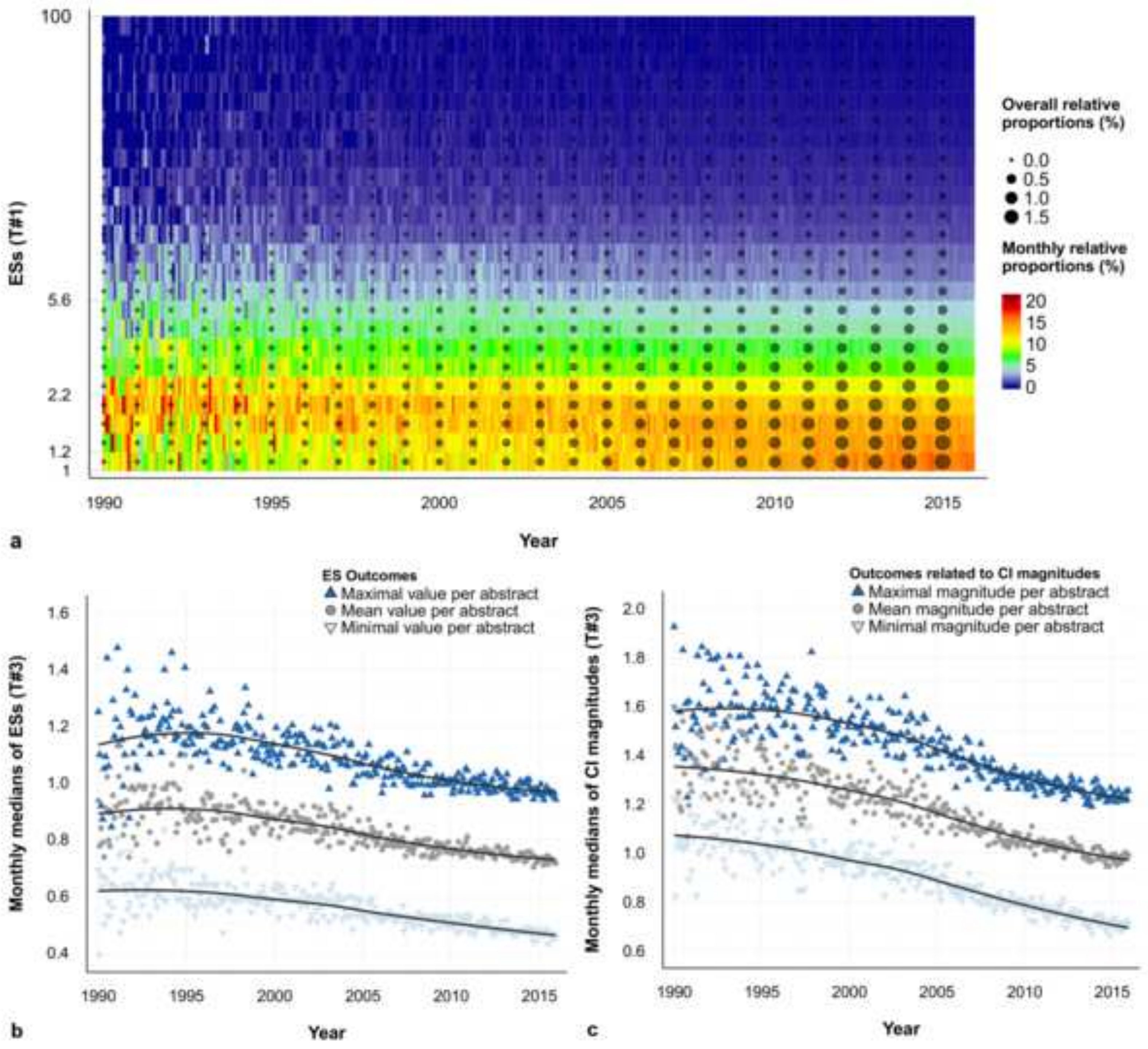

Figure 4

Click here to download Figure Figure 4.tif

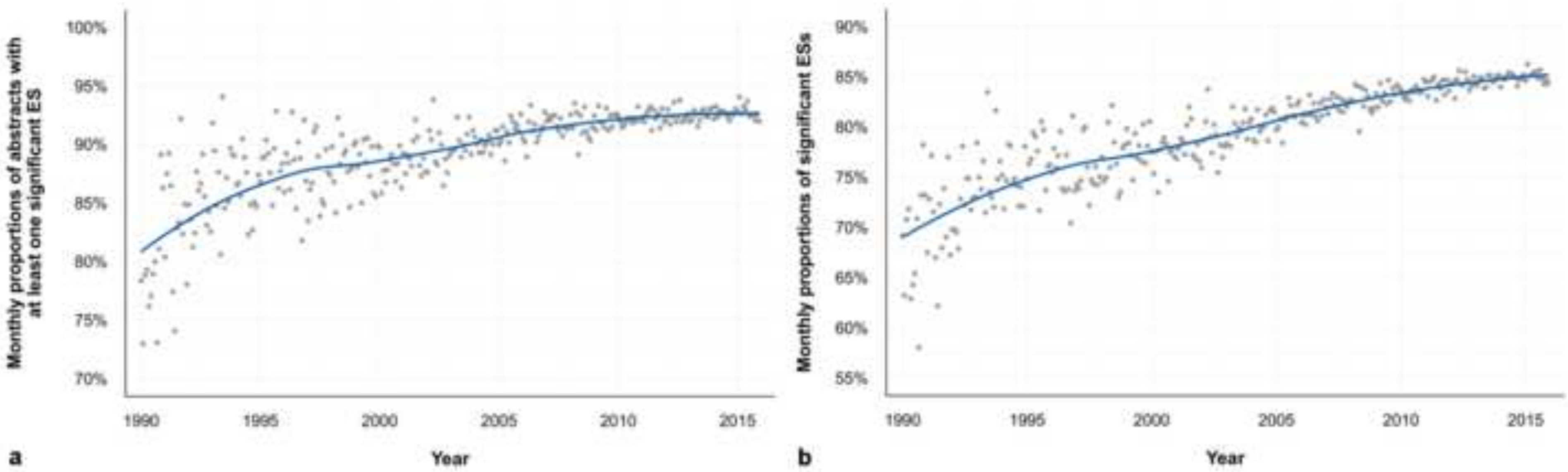

Figure 5 Click here to download Figure Figure 5.tif

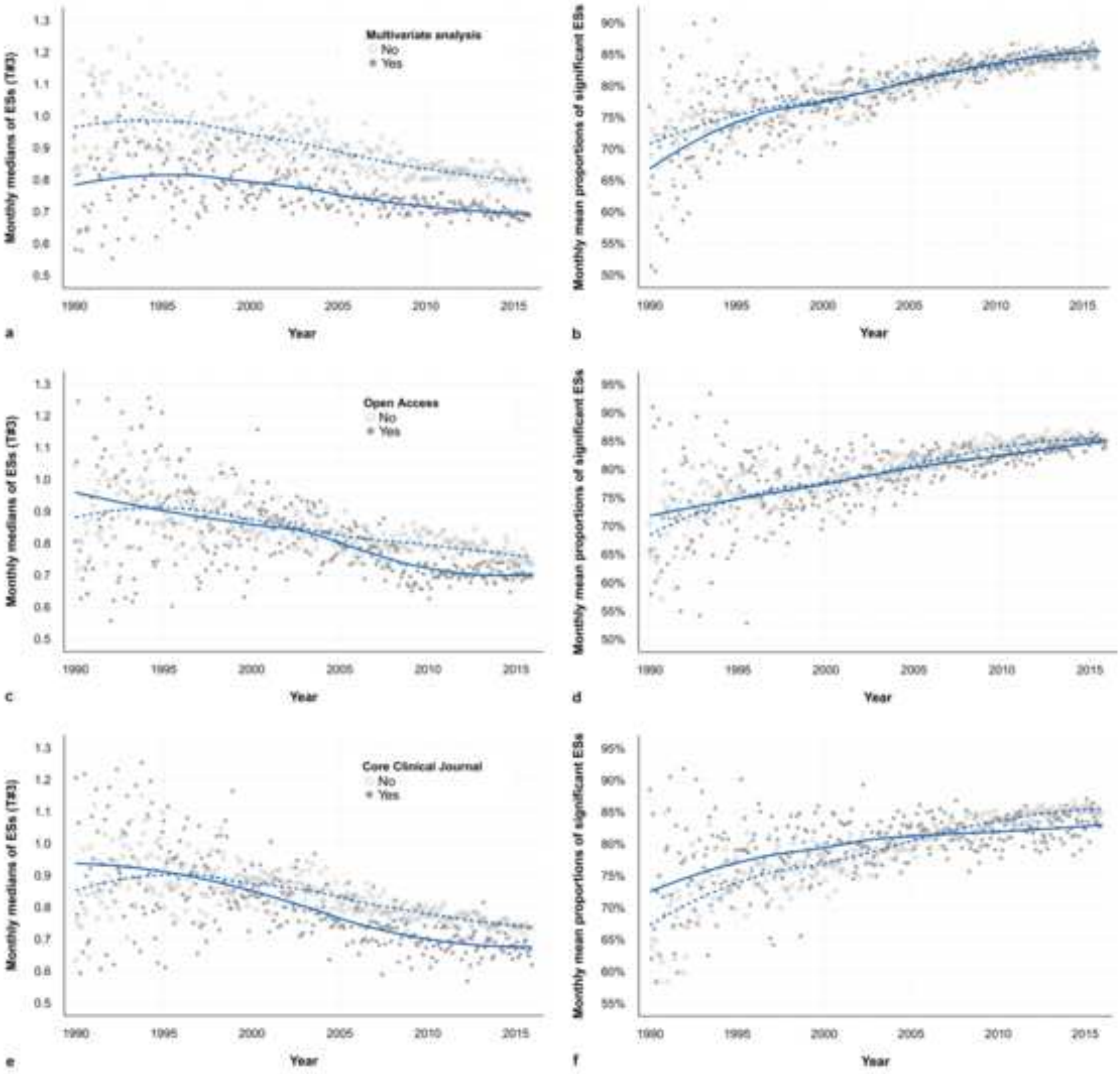

Click here to access/download
Supplementary Material
2017_07_17_Supplementary_data.pdf

Personal Cover
Click here to download Personal Cover
2017_07_17_Cover_letter.docx

Corresponding author
Jean-Noel Vergnes

Paul Sabatier University, Dental Faculty, Department of Epidemiology and Public Health, Toulouse University Hospital, Toulouse, France.
& Division of Oral Health and Society, Faculty of Dentistry, McGill University, Montreal, Quebec, Canada.
Mail address: UFR Odontologie de Toulouse - 3, chemin des maraîchers 31062 Toulouse Cedex 9.
E-mail address: jn.vergnes@mcgill.ca
Telephone number: +33 6 98 00 03 14

July 17nd, 2017

Dear editorial team,

It is with pleasure that we submit our article entitled **"The surprising evolution of effect sizes in biomedical research between 1990 and 2015: smaller but more often statistically significant"** to GigaScience. Here is the brief background story of this paper, together with some arguments as to why we believe it should be of considerable interest to the readers of GigaScience.

All authors have approved the manuscript for submission, the content of the manuscript has not been published, or submitted for publication elsewhere and we have no potential competing interests.

Origin of this viewpoint

The idea for this viewpoint dates from autumn 2015. We had just finished sizeable studies mapping clinical trials in the spheres of periodontal medicine (1) and stem cells (2,3), two fields with natural connections to the work carried out for our individual PhDs. There is little relation between these fields and yet we noticed marked similarities in the study results. In particular, they left us in no doubt that the global research environment, at least in these two fields, was increasingly encouraging the **emergence of new themes** and that researchers tended to **follow the latest trend** more and more as time went on (see, for example, Figure 1, taken from the JCP article (1)).

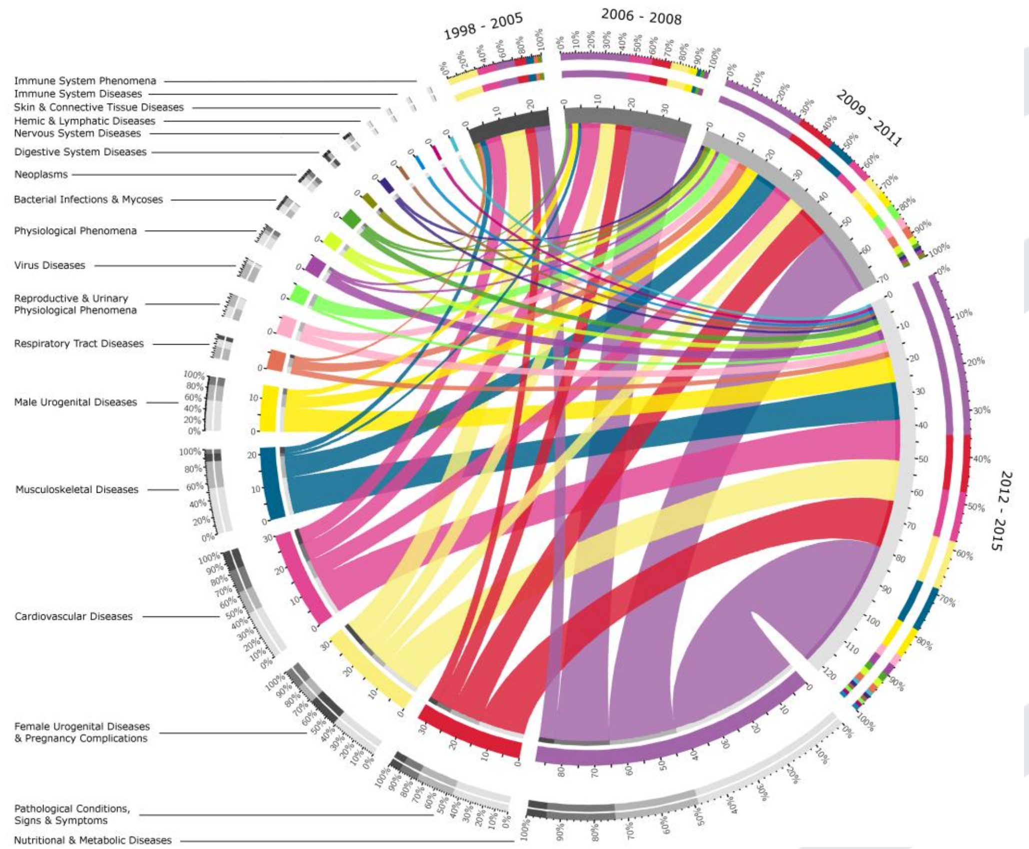

Figure 1. Connectogram of the temporal evolution of topics in periodontal medicine. This chord diagram represents the proportion of registered trials dealing with each sub-branch of the MeSH classifications "Diseases" [C] and "Phenomena and Processes" [G], linked to the respective start years of the trials (grouped in 4 periods: 1998–2005, 2006–2008, 2009–2011, 2012 and beyond). We can see a snowball effect: minor topics in one period are likely to become intermediate topics later (e.g. musculoskeletal diseases, or male urogenital diseases). Finally, there appears to be a trend towards increasing diversification in the registration records.

This demonstration of “fashions” in biomedical research resonated with numerous observations by JPA Ioannidis & colleagues, whose work we have followed with interest over the past ten years or so. They have repeatedly shown how the “publish or perish” environment in science favors the emergence of results that are biased or even false (4,5). Our experience in carrying out systematic reviews leads us to share their fear that contemporary research will become centered more around publication (and potential impact in the media) than around the aim of contributing to true advances in knowledge. A number of cartoons have already caricatured this trend (Figure 2).

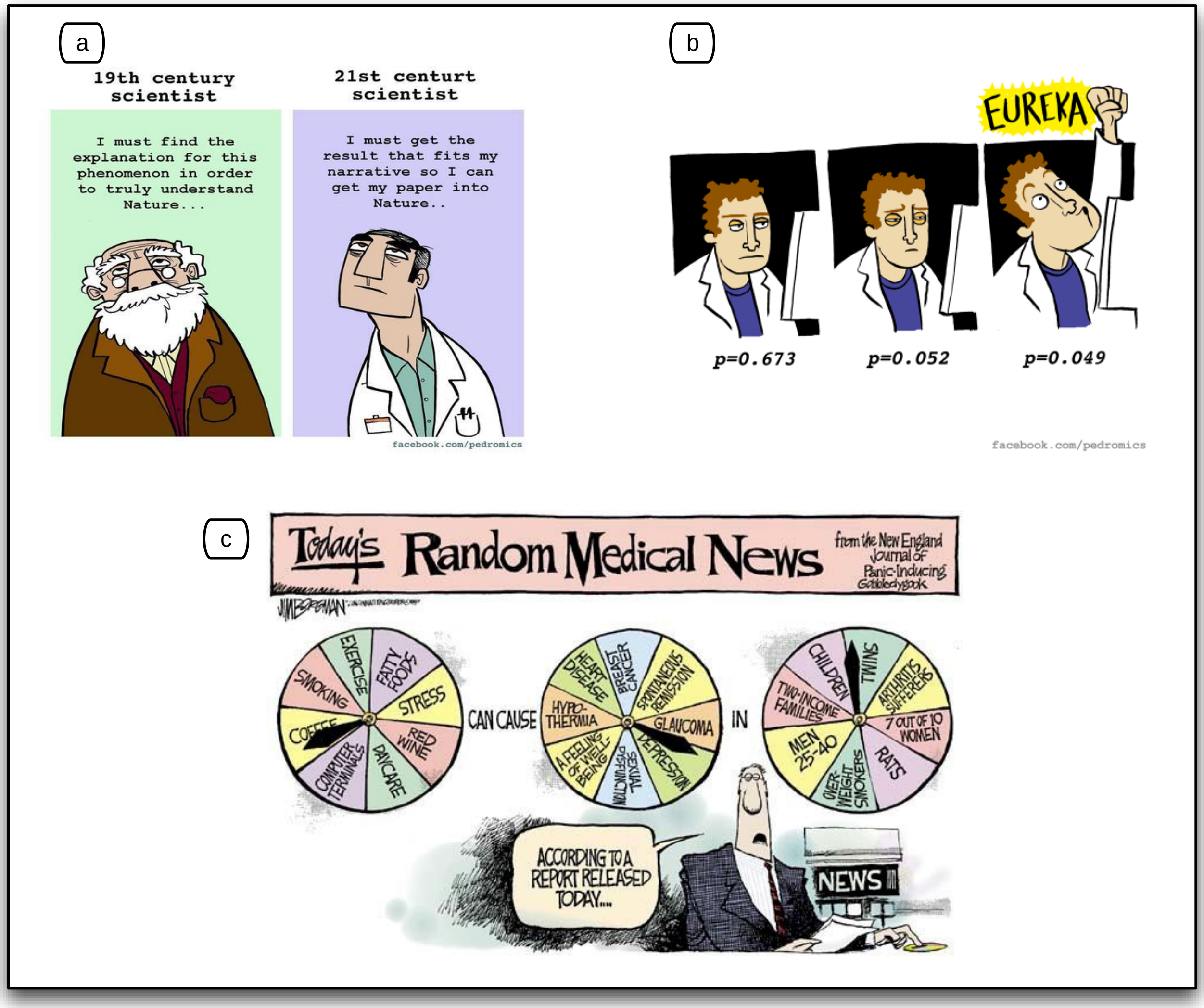

Figure 2. Examples of humorous drawings: (a) and (b) from facebook.com/pedromics, and (c) cartoon by Jim Borgman, first published by the Cincinnati Inquirer and King Features Syndicate 27 Apr 1997; Forum section: 1, and reprinted in the New York Times, 27 April 1997, E4.

By performing an *in-depth* bibliography, we discovered the article published by Gary Taubes in 1995 (6). The title was explicit: **“Epidemiology faces its limits”**. He evoked the problem of contradicting results in epidemiology and the questionable scientific relevance of small effects in observational studies. But what about epidemiology now, in 2017?

Using data mining methods on the complete PubMed database, we observe that **there have been profound changes in the way epidemiological studies are designed, analyzed and reported since 1990.** These changes may have important impacts on the way the scientific community and the public would interpret results from modern epidemiological studies. This article aims to accelerate research towards the understanding of these complex changes.
We hope you will consider an article of this type suitable for submission to your journal. This manuscript is original. If you would like any additional information we will be very happy to supply it and any suggestions of ways to improve the paper will be gratefully received.

Thank you very much for considering our submission inquiry,

The authors

3, chemin des Maraîchers - F - 31062 Toulouse Cedex 9 - Tel. +33 (0) 6 98 000 314

Paul Monsarrat (PhD in Physiopathology, DDS) is a post-doctoral researcher affiliated to the CNRS 5273 team; UMR STROMALab; University of Toulouse UPS; INSERM U1031; EFS Pyrenees – Mediterranean; Toulouse.
Short biosketch: I have been working in collaboration with Prof. P. Ravaud (French Cochrane Centre, INSERM U1153, Paris, France - http://clinicalepidemio.fr/?page_id=2116&lang=en) since 2014 on the development of computer based programs to automate the extraction of data from some large medical databases (ClinicalTrials.gov, PubMed). My field of interest also includes the implementation of graphical methods to better present and interpret results arising from "big data". I have also published various works in several disciplinary fields. https://scholar.google.ca/citations?user=Oem4so8AAAAJ&hl=en
Key-words: bioinformatics, systematic review, meta-analysis, data mining, computer coding, statistics, biology.

Jean-Noel Vergnes (PhD in Epidemiology/Applied Mathematics, DDS) is a senior lecturer in the Department of Epidemiology and Public Health - Paul Sabatier University, Dental Faculty, Toulouse, France. He is also an adjunct professor in the Oral Health and Society Research Unit of McGill University - Montreal, Quebec, Canada.
Short biosketch: Initially involved in the conduct of systematic reviews of the literature and working with the Cochrane Collaboration, I have participated in the design and analysis of different types of studies (randomized clinical trials, observational studies, qualitative research), and published in various areas (American Journal of Obstetrics and Gynecology, Journal of the American Dental Association, Journal of Medical Ethics, Annals of Internal Medicine, International Journal of Whole Person Care, etc.). https://scholar.google.ca/citations?user=G3-Jvi0AAAAJ&hl=en
Key-words: systematic review, humanities, statistics, ethics, meta-analysis, epidemiology, whole person care.

References

1. Monsarrat P, Blaizot A, Kémoun P, Ravaud P, Nabet C, Sixou M, et al. Clinical research activity in periodontal medicine: a systematic mapping of trial registers. J Clin Periodontol. May 2016;43(5):390‑400.

2. Monsarrat P, Kémoun P, Vergnes J-N, Sensebe L, Casteilla L, Planat-Benard V. Spatial and temporal structure of the clinical research based on mesenchymal stromal cells: A network analysis. Cytotherapy. 17 Oct. 2016;

3. Monsarrat P, Vergnes J-N, Planat-Bénard V, Ravaud P, Kémoun P, Sensebé L, et al. An Innovative, Comprehensive Mapping and Multiscale Analysis of Registered Trials for Stem Cell-Based Regenerative Medicine. Stem Cells Transl Med. June 2016;5(6):826‑35.

4. Ioannidis JPA. Why most published research findings are false. PLoS Med [Internet]. August 2005 [cited 5 May 2016];2(8). Available at: http://www.ncbi.nlm.nih.gov/pmc/articles/PMC1182327/

5. Schoenfeld JD, Ioannidis JP. Is everything we eat associated with cancer? A systematic cookbook review. Am J Clin Nutr. 1 Jan. 2013;97(1):127‑34.

6. Taubes G. Epidemiology faces its limits. Science. 14 July 1995;269(5221):164‑9.
