# Supplementary material for: The intriguing evolution of effect sizes in biomedical research over time: smaller but more often statistically significant
Source: Gigascience. 2017 Dec 6;7(1):gix121. doi: 10.1093/gigascience/gix121 (PMC5765564; doi:10.1093/gigascience/gix121)
Supplement: Supplemental materials [file gix121_supp.zip › Supplementary File 1.pdf]

Back to main menu

Trends of effect sizes in biomedical research, 1990-2015

Version dated Sat Feb 4 23:01:46 2017

Specificity and Sensitivity

Specificity for abstracts is 99.91%
Sensitivity for abstracts is 94.99%

Specificity for ESs is 99.85%
Sensitivity for ESs is 96.49%

Kappa

Kappa value for abstracts is **1** **Excellent**
Kappa value for ESs is **0.97** **Excellent**

Summary table

	Not double checked	Concordant	Discordant
		10232713, 10519476, 10630149, 11427386, 11668164, 11870160, 12052584, 12127195, 12360167, 12612248, 12645672, 12796331, 1452455, 14568895, 14713711, 14991750, 15486068, 15605284, 15660776, 15728400, 1593296, 15976172, 16032259, 16148605, 16441469, 16463382, 16493200, 17096437, 17356799, 17436130, 17635599, 17689261, 17852945, 17879493,	

**True Positive**	None	18055733, 18222376, 18275441, 18312617, 18349283, 18641418, 18773998, 18946466, 19057954, 19131353, 19460104, 19556329, 19713451, 19762746, 19821309, 2029495, 20472937, 20538697, 20644620, 20825763, 20847970, 20868465, 20920807, 21315839, 21453469, 21475076, 21484279, 21519059, 21538389, 21584976, 21590571, 21631300, 21632080, 21665043, 21750273, 21768315, 21872930, 22021664, 22023667, 22092606, 22116145, 22352417, 22367574, 22370314, 22435183, 22513920, 22517538, 22926605, 22942268, 22965460, 22972110, 22981555, 22983988, 23121297, 23131377, 23152271, 23162099, 23231085, 23325106, 23364011, 23482242, 23485597, 23541794, 23587032, 23647623, 23773275, 23780375, 23798942, 23810564, 23894450, 23948440, 23982253, 23990434, 23996688, 24014144, 24276781, 24468999, 24482059, 24510598, 24532467, 24586635, 24715396, 24735592, 24859820, 24918321, 24970348, 25006638, 25028975, 25111771, 25122052, 25156527, 25209721, 25222655, 25292060, 25300169, 25411602, 25418654, 25457028, 25495993, 25510744, 25564120, 25651929, 25695003, 25735369, 25753294, 25892162, 25988241, 26064247, 26109961, 26112715, 26166212, 26214203, 26271447, 26350797, 26395344, 26434935, 26546117, 26560469, 26607935, 26670673, 26680142, 26751784, 26822042, 26893929, 26941057, 26956487, 27100449, 27125872, 27297704, 27312697, 27564779, 27605685, 27664555, 27684821, 27939278, 27943267, 3457968, 7435487, 7528370, 8050950, 8465022, 8601227, 8829749, 9088438, 9506204, 9794128,	None
		10024429, 10030687, 10036715, 1003821, 1004719, 10051825, 10063992, 10064590, 10067385, 10067402, 10067671, 10073458, 10074784, 10074812, 10076966, 10079381, 1008032, 10080441, 10084377, 10085683, 10086659, 10088014, 10090691, 10096403, 10098688, 10099130, 10106235, 1010681, 1011264, 1014002, 10141761, 1014783, 10148663, 10152651, 10152845, 10153273, 10155272, 10159956, 10163768, 1016741, 10168539, 10171013, 10184075, 10185823, 10191794, 10193787, 10194026, 10194547, 10199073, 10201916, 10203839, 10204074, 1020520, 10208371, 10208729, 10210787, 10211696, 10212607, 10213303, 10216092, 10216222, 10221380, 10231342, 10231509, 10233038, 10233128, 10234637, 10240151, 10249572, 10275905, 10281281, 10285531, 10296802, 10300680, 10300874, 1030221, 10303954, 1030880, 10309327, 10312606, 10313276, 10320275, 10326359, 10328886, 10330251, 10332041, 10334448, 10338069, 10338208, 1034027, 10344227, 10349440, 10349559, 10354154, 10354450, 10354960, 10357262, 1035781, 10357942, 10359012, 10366986, 10369818, 1037208, 10375797, 10376027, 10377209, 10377324, 10379578, 10382278, 10386009, 10386582, 10386843, 10389448, 10390385, 10392465, 10393814, 10397994, 10398113, 10402396, 10403015, 10407092, 10415328, 10415855, 1041797, 10427769, 10429822, 10431453, 10432150, 10432796, 10435584, 10436398, 10437279, 10438973, 10441771, 10443935, 10445250, 10445620, 10446465, 10449574, 10450414, 10452463, 10455838, 10456065, 10456701, 10457062, 10458777, 10459907, 10461461, 10469368, 10469880, 10470168, 10471344, 10477172, 10480646, 10485503, 10486150, 10486490, 10487167, 10487742, 10488206, 10488332, 10489681, 10489697, 10493234, 10494852, 10494945, 10497925, 10498190, 10501855, 10504142, 10504309, 10505065, 10505703, 10506199, 10507702, 10512733, 1051521, 10517953, 10518032, 10523544, 10526490, 10527502, 10528425, 10529661, 1053260, 10535280, 10536670, 10537167, 10537878, 10538421, 10540956, 10541302, 10545211, 10545634, 10546024, 10553614, 10557442, 10564267, 10564536, 10570287, 10578564, 10580297, 10587112, 10588700, 10590274, 10590457, 10591242, 10592880, 10596015, 10600433, 10600860, 10601094, 10601495, 10601740, 10603291, 10604816, 10609298, 10610260, 10611323, 10611608, 10612343, 10613388, 10616095, 10616691, 10628855, 10629730, 10629852, 10630676, 10631025, 10636635, 10637838, 10642328, 10646803, 10648742, 10652981, 10654596, 10655118, 10655979, 10657214, 10662471, 10664459, 10667496, 10667864, 10668836, 10669107, 10671806, 10672321, 10672906, 10673704, 10675487, 10677970, 10680098, 10680509, 10682567, 10683130, 10683714, 10684991, 1068506, 10685789, 10685899, 10686022, 10689392, 10690972, 10690992, 10694406, 10697026, 10701566, 10703512, 10703725, 10704230, 10704250, 10706185, 10707079, 10709461, 10712330, 10713723, 10714317, 10714404, 10715286, 10717803, 10719273, 10719814, 10721171, 10722203, 10724982, 10726606, 10726703, 107271, 10730326, 10731082, 10731429, 10731912, 10731989, 10732983, 10735427, 10737958, 10738532, 10740417, 10740937, 10741261, 10742057, 10743666, 10747466, 10754532, 10762518, 10762718, 10768107, 10769954, 10775427, 10775765, 10776810, 10780388, 10784434, 10788261, 10788718, 10789449, 10790170, 10790819, 10793134, 10800546, 10802840, 10803235, 10805695, 10805986, 10809843, 10809977, 10811868, 1081410, 10819020, 10820429, 10824117, 10825351, 10826295, 10829390, 10834031, 10836032, 1083696, 10841078, 10841912, 10842740, 10843337, 10843633, 10845813, 10847328,	

10848160, 10849843, 10851266, 10853291, 10859480, 10861043, 10863912, 10863959, 10866291, 10866389, 10867453, 10867510, 10872395, 10873487, 10874370, 1087870, 10880152, 10880495, 10882514, 10884346, 10886448, 10889321, 10889820, 10890297, 10896042, 10898311, 10900024, 10901662, 1090179, 10902800, 1090432, 10908729, 10912825, 10914598, 10915003, 1091904, 10921737, 10923827, 10924043, 10927731, 10929732, 10929949, 10930474, 10933700, 10937345, 10939540, 10943544, 10944919, 10944950, 10947529, 10947633, 10949350, 10952350, 10958294, 10958404, 10960762, 10964047, 10967835, 10969524, 10970966, 10974358, 10975609, 10976391, 10979328, 10980731, 10982291, 10985994, 10987862, 10987929, 10995208, 10998790, 11001602, 11003349, 11003395, 11003728, 11004350, 11005076, 11005995, 11006496, 11006960, 11009335, 11013728, 11016690, 11018527, 11019790, 11021466, 11024029, 11029116, 11029273, 11031251, 11033324, 11034858, 11035025, 11038190, 11039348, 11044203, 11045840, 11052345, 11055382, 11056884, 11061808, 11062572, 11062723, 1107093, 11074949, 11077452, 1107836, 11078714, 11082488, 11084642, 11085274, 11085292, 11087999, 11088131, 11091061, 11091198, 11092812, 11093036, 11093413, 11093536, 11094678, 11095564, 11096067, 11097444, 1109804, 11102198, 1110418, 11105470, 11106684, 11108084, 11109412, 11110963, 11112665, 11113560, 111171, 11118503, 11118801, 11121684, 1112251, 1112257, 11123496, 11125413, 11127197, 1113011, 11133263, 11135396, 11141334, 11142848, 11147816, 11148775, 11153775, 11154965, 11154979, 1115551, 11156583, 11157087, 11158920, 11159045, 11159163, 11159726, 11159744, 11160627, 11161132, 11161507, 11163476, 11163691, 11163731, 11164791, 11166255, 11167789, 11170564, 11172415, 11175195, 11175482, 11176717, 11179012, 11181324, 11181480, 11183229, 11189245, 11189839, 11189891, 11190598, 11193895, 11194181, 11200543, 11206722, 11209501, 11212105, 11214625, 11218259, 11218874, 11219615, 11222378, 11222587, 11222902, 11223252, 11225590, 11229418, 11229601, 11233930, 11234969, 11240623, 11242490, 11245217, 11248884, 11249557, 11256259, 11258893, 11261648, 11262461, 11263338, 11271030, 11277856, 11280548, 11280744, 11281324, 11282661, 11283857, 11284967, 11289812, 11292226, 11292806, 11296447, 11299787, 11301855, 11309509, 11312472, 11317346, 11317353, 11323863, 11325162, 11326984, 11327619, 11330215, 11331368, 11333310, 11334738, 11336111, 11338665, 11338967, 11340630, 11343789, 11343947, 11345927, 11347390, 11350038, 11351404, 11352149, 11355482, 11358522, 11359718, 11361954, 11368876, 1137357, 11375338, 11377725, 11377839, 11379976, 11381112, 11381123, 1138551, 1138852, 11390640, 11397072, 11397412, 11397847, 11403099, 11403990, 11409128, 11413749, 11418436, 11422628, 11423480, 11426792, 11427169, 11428699, 11430482, 11432296, 11433139, 11441919, 11441920, 11448034, 11449803, 11451368, 11452864, 11455292, 11461047, 11464426, 11465047, 11465071, 11467199, 11469521, 11471348, 11471662, 11472682, 11476775, 11479396, 11481345, 11483673, 11485389, 11490085, 11490218, 11490297, 11490975, 11491382, 11493424, 11493465, 1149805, 11503946, 11505683, 11506459, 11508351, 11513411, 11517510, 11520566, 11522336, 11523012, 11523938, 11524553, 11529688, 11533844, 11533978, 11534200, 11534732, 11534788, 11538967, 11539145, 11540369, 11542624, 11542887, 11543762, 11547900, 11549310, 11554361, 11556090, 1155795, 11559764, 11560034, 11560901, 11561075, 11563341, 11565029, 11565075, 11565481, 11568584, 11571031, 11572975, 11574419, 11576057, 11576703, 11577091, 11580815, 11587465, 11587530, 11587877, 11588364, 11589997, 11594155, 11595982, 11599555, 11602203, 11604517, 11607075, 11607779, 1161240, 11625008, 11642688, 116471, 11666366, 11668212, 11669166, 11671134, 11672302, 11673852, 1167407, 11674691, 11676534, 11682427, 11684866, 11685208, 11697686, 11698281, 11699227, 11699544, 11703574, 11704440, 11706213, 11712631, 11713848, 11715194, 11716709, 11716987, 11717896, 11718374, 11723287, 11732949, 11744199, 11745036, 11745089, 1174717, 11749748, 11750192, 11753566, 11753932, 11755952, 11756453, 11757015, 11759763, 11760609, 11761772, 11763553, 11764159, 11764361, 11765224, 11767867, 11768625, 1177158, 11771976, 11773426, 11774822, 11775649, 11775662, 11779133, 11779554, 11782183, 11784164, 11785592, 11785632, 11787698, 11787977, 11788376, 1178851, 11792442, 11792678, 11797296, 11799731, 11800925, 11807441, 11812720, 11816646, 11816824, 11818363, 11820018, 11823451, 11823497, 11825264, 11825983, 1182706, 11827473, 11827711, 11828376, 11831316, 11831596, 11831984, 11837023, 11841080, 11845560, 11845886, 11846180, 1184665, 11847623, 11849709, 11850041, 118511, 11851798, 1185223, 11852647, 11853506, 11854625, 11860605, 11861990, 11864542, 11865362, 11869780, 11870546, 1187188, 11872152, 11872259, 11874009, 11881274, 11883180, 11894534, 11894842, 11897763, 11900053, 11907736, 11908611, 11912491, 11912930, 11913857, 11913952, 11913962, 11913991, 11914383, 11921170, 11921721, 11923096, 11924450, 11926976, 11931138, 11938772, 11942003, 11942367, 11942771, 11950892, 11951487, 11954557, 11957385, 11957451, 11960902, 11961862, 11966864, 11969900, 11969923, 11969929, 1197144, 11971672, 11972981, 11976380, 11980456, 11993068, 11995908, 11998834, 11999196,

		1200028, 12000665, 12002558, 12002759, 12003262, 12011847, 12012881, 12013358, 12016765, 12017506, 12019349, 12020032, 1202224, 12023552, 12024847, 12026455, 1202744, 12030992, 12034757, 12036059, 12037217, 12037424, 12039082, 1204581, 12048373, 1205050, 12051341, 12057115, 12060130, 12060482, 12062538, 12065542, 12073096, 12081141, 1208189, 12082452, 12083578, 12086347, 12086444, 12088608, 12093821, 1209891, 1210122, 12101350, 12102767, 12104061, 12105938, 12107585, 12107757, 12111989, 12112349, 12113675, 12113956, 12116882, 12121075, 12121369, 12121708, 12126149, 12126252, 12127405, 12131848, 12131952, 12135893, 12143173, 12144143, 12146996, 12147774, 12153236, 12153323, 12162051, 12162489, 12163305, 12164073, 12167703, 12169627, 12170812, 12175743, 12176734, 12177164, 12177575, 12182851, 12183332, 12190161, 12190599, 12196081, 12198764, 12201154, 12202595, 12206665, 12209292, 12209376, 12212861, 1221335, 12217880, 12221745, 12223400, 12225081, 12227676, 12227755, 12235371, 12236559, 12237804, 12242965, 12243779, 1224780, 12283178, 12289429, 12324447, 1232998, 12344796, 123503, 12351217, 12353753, 12366144, 12368267, 12371162, 12375251, 12379310, 12381539, 12391144, 12394831, 1239641, 12399460, 12400811, 12404880, 12405271, 1240691, 12408278, 12409379, 12411028, 12415219, 12417030, 12419204, 12419746, 12420113, 1242111, 12421550, 12425778, 12425791, 12426473, 12432701, 12433286, 12437814, 12438394, 12439174, 12440892, 12441038, 1244243, 12444377, 12447694, 12448042, 12451984, 12453503, 12456906, 12458207, 12458397, 12459716, 12467239, 12471887, 1247235, 12472657, 12473553, 12476242, 12482123, 124885, 12492651, 1250280, 12505963, 12505970, 1250629, 12507536, 12513501, 12514282, 1251768, 12523734, 12525021, 12527239, 12527559, 12527763, 12531257, 12532242, 1253381, 12536041, 12538422, 12539589, 12552554, 1255565, 12561804, 12562378, 12567858, 12568647, 12568953, 12575378, 12575877, 12577674, 12587219, 12588534, 12598255, 12605613, 12605614, 12609049, 12609844, 12610320, 12610766, 12611258, 12614575, 1262131, 12623158, 12628117, 12628199, 12628320, 1263197, 12638674, 12641494, 12642607, 12643724, 12643945, 12646742, 12646761, 12649292, 12652422, 12669628, 12670172, 12670903, 12679530, 12680624, 12688631, 12688813, 12688921, 12691082, 12699124, 12700391, 12701515, 12702008, 12708700, 12709016, 12713162, 12713965, 12714861, 12715909, 12716666, 12717148, 12717250, 12720361, 12723949, 12724397, 12727464, 12732680, 12733924, 12735618, 12736461, 12736718, 12739735, 12742198, 12743298, 12744346, 12745012, 12745452, 12749977, 1275474, 12761967, 1276219, 12762394, 12762807, 12763623, 12765424, 12765825, 12768866, 12771792, 12773728, 12775848, 12778581, 12780144, 12787047, 12790326, 12792257, 12794107, 12796055, 12797489, 12799020, 12799476, 12808228, 12809570, 12810463, 12813334, 12813593, 12816395, 12818536, 12820890, 1282098, 12822382, 12823383, 12824265, 12825953, 12826395, 12828934, 12830452, 1283060, 12831590, 12832802, 12833141, 12841700, 12842179, 12843439, 12851616, 12853172, 12855587, 12856306, 12858510, 12862377, 12868787, 12873099, 12874674, 12880729, 12881513, 12884159, 12887048, 12888486, 12892388, 12899196, 12900861, 1290299, 12905068, 12905598, 12906056, 12913000, 12913722, 12917256, 12917440, 12920525, 12921452, 1292579, 12926704, 12929713, 1293143, 12932419, 12935912, 12940424, 12941218, 12941515, 12942340, 12946547, 12949321, 12955623, 12962467, 12963012, 12963076, 12963379, 12966104, 12967008, 12967873, 12971157, 12971489, 12972706, 1302157, 1305643, 1306124, 130860, 1309279, 13130146, 1316564, 1317441, 1318794, 131944, 1319510, 1319701, 1322118, 1322528, 13245, 1325992, 1328480, 1328846, 133085, 1334099, 1334528, 1339317, 1339858, 1341433, 1345675, 134720, 1350147, 1356722, 1357133, 1358489, 1361007, 1365036, 13658986, 13678608, 1371179, 1376411, 1376493, 137867, 1380378, 138046, 1382374, 1382445, 1386617, 1395944, 1396088, 1400302, 1400851, 1402373, 1404530, 1407736, 1410701, 14113110, 1411323, 1414847, 1416441, 14169722, 1420883, 1422081, 1423046, 1423665, 1427414, 1427787, 1428396, 1438302, 1441797, 1443094, 1443332, 1444645, 14499101, 14500580, 14505490, 14505760, 1450841, 14509418, 1451223, 14512433, 14514260, 14516545, 14518482, 14527059, 14529791, 14531426, 1453353, 1454687, 14550375, 1455169, 14557490, 14559057, 14567251, 14567472, 14567543, 14568905, 14575838, 14589735, 14592451, 14594799, 14597116, 14598043, 14600077, 14607163, 14610269, 14610951, 14614050, 14614637, 14621688, 14625477, 14625684, 14630220, 14636268, 14639603, 14640219, 14640818, 14641618, 14643345, 14647409, 14654323, 14656600, 14660720, 14660788, 14661141, 14662060, 14663885, 14664613, 14666321, 14666829, 1466778, 14669247, 14669318, 14670202, 14671443, 1467372, 14677056, 14678172, 14679045, 14680729, 14682480, 14685246, 14687280, 14688995, 14689121, 14690160, 14690256, 1469064, 14692462, 14692758, 14693708, 14696522, 14698466, 14699603, 14700598, 14706599, 1470664, 14708653, 1471153, 14717232, 14719710, 14721991, 14723377, 14725220, 14728859, 14734200, 14735357, 1473538, 1473805, 14744447,	

14744891, 14745572, 14746354, 14747464, 14747501, 14757365, 14762075, 1476241, 14763047, 14767501, 14768402, 14768538,
1477640, 147816, 14821823, 1482609, 1482762, 1482870, 1482946, 1484656, 1487953, 148917, 1495893, 14961681, 14962781,
14963119, 14971128, 14971959, 14974710, 14975940, 14977969, 14978409, 14978651, 14981089, 1498451, 14987706, 14988247,
14989682, 14992431, 14993745, 15003264, 15006656, 15008251, 15012179, 15013151, 15016857, 15017409, 15017490,
15018737, 15019239, 15019447, 15019951, 15021911, 15021971, 15023342, 15025426, 15028001, 15028558, 15030454,
15033838, 15033955, 15035265, 15035882, 15037310, 15038633, 15041351, 15044028, 15045109, 15045675, 15047266,
15049808, 15051200, 15052346, 15054352, 15055325, 1505670, 15058789, 15065931, 15067821, 15068283, 15071450, 15072295,
15081326, 15082797, 1508394, 15084488, 15084504, 15084657, 1508649, 15086795, 15088070, 15090321, 15091229, 15091386,
15093113, 15093789, 15096446, 15100807, 15101397, 15101671, 15102521, 15105219, 15106649, 15107699, 15108770, 1511013,
15110560, 15110978, 15115281, 15115361, 15115986, 15116426, 15122304, 15123727, 15126661, 15128592, 15130305,
15132265, 15132301, 15132672, 15142000, 15146907, 15151889, 1515251, 15156835, 15157327, 15160258, 15160667, 15162209,
1516562, 15166456, 15168463, 15168858, 151716, 1517308, 15176187, 15176424, 15181212, 15181626, 15183069, 15184711,
15187855, 15191319, 15191931, 15193349, 15193846, 15197148, 15201001, 1520460, 15207046, 15209229, 15213874, 15219543,
15219705, 15220937, 15223365, 15231861, 15233782, 1523675, 1523767, 15238877, 15240567, 15240607, 15242169, 15243162,
15245960, 15250070, 15251218, 15255846, 15258486, 15259024, 15260317, 15261652, 15264502, 15265478, 15266077, 1526696,
15267975, 15268029, 15269897, 1527060, 15272732, 15276214, 1527655, 15279999, 15284269, 15286530, 1529636, 15296559,
15296711, 15297527, 15299351, 15303916, 15304520, 15305982, 15307593, 15312079, 15313015, 15313449, 15313569,
15313637, 15313656, 15317850, 15323226, 15323413, 15324364, 15324465, 15324685, 15325166, 15327404, 15328108,
15330191, 15331535, 15332798, 153343, 15335585, 15337022, 1533877, 15342267, 1534356, 15344529, 15347330, 15347634,
1534805, 15348667, 15352652, 1535293, 15353889, 15354009, 15355086, 15358021, 15358517, 15358800, 15359000, 15362742,
15363057, 15364153, 15365825, 15366301, 15372249, 1537323, 15374520, 15375648, 15376206, 15384814, 1538696, 1538751,
15389943, 1539625, 1540569, 1544493, 1544710, 1544720, 15450116, 1545036, 15451363, 15451729, 15452290, 15456205,
15459146, 15459330, 15460937, 15467412, 15468398, 15469651, 15470125, 15474671, 15476420, 15476998, 1548493, 15487268,
15489580, 15494030, 15498111, 15501641, 15503808, 1550496, 15505279, 15509507, 15513388, 15513438, 15514373, 15514856,
15516879, 15517484, 15522913, 15527314, 15534562, 15535119, 15535194, 15539081, 15540838, 15542599, 15542710,
15543403, 15544632, 15547978, 15549669, 15550901, 15556284, 15556434, 15563431, 15564061, 1556537, 15565539, 15570938,
15582561, 15584721, 1558777, 15592987, 15597199, 15597586, 15609208, 15610187, 15611192, 15613307, 15617020, 15617514,
15618513, 15620663, 15621121, 15624081, 15626655, 15629800, 15630540, 15634083, 15634777, 15638977, 15639653, 1563974,
15642044, 15645306, 15649668, 15650870, 15652697, 1565311, 15653682, 15654425, 156560, 15656074, 15656218, 15658689,
15661477, 15663979, 15664428, 15668117, 15672680, 15673422, 15674942, 15675321, 15675850, 15676709, 15678173,
15685956, 15690275, 15697794, 15702098, 15705746, 15708927, 15712589, 15713650, 15716535, 15719950, 15720094,
15724846, 15729882, 15730106, 15730266, 15730457, 1573046, 15732045, 15732960, 15734684, 15735119, 15738238, 15738550,
15738787, 15739332, 15740213, 15742329, 15743771, 1575170, 15753434, 15754281, 15759128, 15760630, 15765002, 15765031,
15770475, 15773577, 15775288, 15776183, 15781664, 15782579, 15787195, 15788117, 15791573, 15796701, 15799716,
15808075, 15808339, 15809346, 15812309, 15822081, 15824635, 15827223, 15827795, 15830961, 15832870, 15833266,
15834146, 1583417, 15836289, 15840756, 15841561, 15841757, 1585002, 15850539, 15852852, 15853105, 15857538, 15857797,
15858845, 15861483, 15862496, 15862521, 15863108, 15863882, 15866647, 1586956, 15870640, 15871043, 15871441, 1587552,
15880109, 15884394, 15885332, 15885539, 15888432, 15890222, 15890527, 15891197, 1589316, 15897031, 15902514, 15908051,
15911300, 15911400, 15912314, 15912347, 15912678, 15913762, 15914078, 15927224, 15930927, 15933469, 15933743,
15934905, 15936432, 15937815, 15941646, 15943095, 15944897, 159459, 1594600, 15950455, 15951467, 15955367, 15956880,
15960624, 15961355, 15964513, 15965115, 15968427, 15969381, 15973523, 15974634, 15976992, 15977652, 15978146,
15978974, 15981807, 15986258, 15986602, 15986892, 15990078, 15992754, 15993008, 15994326, 15994722, 15996425,
15999147, 16000954, 16003826, 1600485, 16005188, 16005737, 16011263, 16011615, 16014736, 16015101, 1601769, 16018844,
16019041, 16022461, 16024903, 16025529, 16027038, 16027809, 16028031, 16028095, 16028127, 1602843, 16032364, 16043062,

		16043096, 16049954, 16049976, 16052830, 16055009, 1605576, 16061048, 16078036, 16080796, 16085744, 1608614, 1608716, 16089298, 16090725, 16092865, 16093754, 16094154, 16100749, 16102948, 1611702, 16118452, 16118604, 16119461, 16122349, 16127698, 16128551, 16136284, 16138223, 16139489, 16141521, 16142216, 16143271, 16145073, 16153041, 16154574, 16156967, 16167798, 16169632, 16172623, 16175119, 16176757, 16183666, 16186376, 16187915, 16190810, 16191613, 16203120, 16206463, 1620741, 16212032, 16214016, 1621505, 16218469, 16224670, 16226250, 16229964, 16232170, 16232301, 16234128, 16235333, 16243694, 16247777, 16248123, 16248335, 16249013, 1624958, 1625225, 16255921, 16258237, 16259779, 162632, 16263420, 16263579, 16269176, 16270959, 16274625, 16275296, 1627620, 16277630, 16278078, 16280239, 16280314, 16280384, 16285022, 16289792, 16290100, 16290111, 16295904, 16297255, 16298231, 16300006, 16308551, 16311517, 16319675, 16319881, 1632298, 16327459, 16327531, 16328288, 16328657, 16330024, 16332487, 16336946, 1634246, 16346470, 16347396, 16349060, 16349707, 16352558, 16352722, 16356550, 16357290, 1635857, 16358791, 16364733, 1636720, 16369173, 16369875, 16370459, 16376398, 16376406, 1637969, 16381475, 1638294, 16385623, 16386951, 16387371, 16387606, 16390469, 1639079, 16391706, 16393518, 16397581, 16399781, 16403334, 16405054, 16405193, 16406514, 16407666, 16408277, 16409079, 16410201, 16413934, 16414836, 16415378, 1641613, 16417285, 1641765, 16423275, 16425387, 16427000, 16427101, 16430209, 16431338, 16434174, 16438371, 16440115, 1644186, 16444072, 16445387, 16446832, 16446968, 16449069, 16449654, 16452443, 16453417, 16453607, 16455859, 16458520, 16459863, 16460188, 16467776, 16469915, 16471763, 16471973, 16472182, 16473650, 16478958, 16481210, 16482327, 16482407, 16482570, 16486153, 16486779, 16491537, 164926, 16492665, 16492670, 16495272, 1649730, 16497916, 16501670, 16502226, 16504504, 16507293, 16507487, 16511757, 16513242, 16513899, 16517203, 16521448, 16521588, 16521736, 1652364, 16525114, 16526927, 16527929, 16533780, 16534447, 16535226, 16535836, 16540453, 16541258, 16541784, 16543264, 1654768, 16549036, 16551864, 16553437, 16553782, 16562401, 16562776, 16565051, 16565169, 16568329, 16568902, 16571544, 16574596, 16575173, 16575773, 16580262, 16582589, 16584440, 16585092, 16594391, 16594400, 16594617, 16599844, 16600793, 16605539, 16607155, 16615761, 1662954, 16634038, 16634696, 16638700, 16639153, 16639853, 16640828, 16641144, 16644913, 16645211, 16645956, 1664832, 16651704, 16659650, 16660384, 16663226, 16665018, 16665518, 16667716, 16675158, 166754, 16686710, 16687232, 16689484, 16697297, 16697727, 16698790, 16700389, 16700918, 16703140, 16704845, 16705996, 16706709, 16713648, 16713674, 16713769, 16719599, 16723046, 16723357, 16723558, 1672370, 16727492, 16728187, 16728508, 16728905, 16729118, 16730454, 16730714, 16731295, 16731396, 16734775, 16737736, 16738891, 1674182, 16742556, 1674548, 16755560, 16760403, 16761045, 16761924, 16762174, 1676394, 16767574, 16777128, 16778189, 16787293, 16789785, 16792735, 16793855, 16794855, 16801307, 1680188, 16804659, 1680545, 16805888, 16809429, 16812768, 1681441, 16818526, 16821219, 16821635, 16822963, 16826579, 16828299, 16830913, 16833461, 16834473, 16837900, 16841641, 16845526, 1684560, 16846840, 16852807, 16854058, 16856176, 16857747, 16858268, 16863723, 16864773, 16865391, 16867672, 16867979, 16870338, 16870340, 16874191, 16875579, 16876403, 16877398, 1687909, 16885288, 16887576, 16893474, 168938, 16896814, 16899637, 16900009, 16900325, 16900414, 16900790, 16901685, 16902670, 16904066, 16904729, 16904752, 16905073, 1690597, 16907832, 16909201, 16909780, 16909913, 16910711, 16911002, 16911075, 16912225, 16917857, 16920483, 16922100, 16923593, 1692399, 16924604, 16924983, 16926322, 1692907, 16933419, 16934375, 16936966, 16938703, 16940557, 16941573, 1694620, 1694734, 16949996, 16951891, 1695318, 16954504, 16954592, 16957571, 16957744, 16957791, 16958418, 16958670, 16959485, 16961742, 16962484, 16965871, 16967786, 16969516, 1697099, 16978585, 16978774, 16982124, 16987031, 16987767, 16995629, 16996566, 16997719, 16998319, 1699845, 17000278, 17002308, 17004188, 1700556, 1700714, 17010781, 17010804, 17012693, 17013708, 17014600, 17018766, 17020522, 17022454, 17024955, 17029822, 17030051, 1703273, 17034304, 17034917, 1703495, 17035366, 17035572, 17036745, 17037454, 17039288, 17039396, 17042166, 17043391, 17043425, 1704616, 17050733, 17051420, 17051479, 17055915, 17056563, 17057200, 17058249, 1705903, 17062871, 17064417, 17066117, 17066465, 17070787, 17074763, 17076918, 17077434, 17081056, 17081666, 17082199, 17083288, 17086896, 17087588, 17088263, 17088494, 17089759, 17090019, 17091439, 17091749, 17096069, 17097279, 1709784, 17097965, 17101109, 17107787, 1710949, 17113191, 17115108, 17115926, 17116517, 1711895, 17123614, 17123972, 17124258, 1712535, 17125718, 1712588, 17129226, 17129454, 1713048, 171306, 17142595, 17142997, 17143956, 17146377, 17151404, 17156298, 17157730, 17159673, 17161806, 17165033, 1716824, 17168524, 17170964, 17173076, 17176956, 1717734, 17177999, 17178176, 17181321, 17182163, 17185277,

		17188408, 17194160, 17196904, 17198758, 1719925, 17201427, 17203430, 17204297, 172045, 17204646, 17207870, 17208962, 17210449, 17211865, 17216341, 17217128, 17217448, 17219059, 17221195, 17223032, 17223187, 17226980, 1723073, 17235300, 17236246, 17238871, 17239293, 17240929, 17242655, 17242683, 17243045, 1724357, 17246153, 17246174, 17248880, 17249234, 17250642, 17255033, 17257723, 17261643, 17263499, 17263610, 17265223, 17266984, 17267252, 17268698, 17269273, 17271177, 17273412, 17276420, 17279769, 17284375, 17285643, 17286985, 17292132, 17294750, 17301882, 17302811, 17303027, 17303366, 17306510, 1730771, 17308121, 17308824, 17311298, 17314071, 17316937, 17317744, 17319956, 17322541, 17323074, 17326690, 17326957, 17329388, 17330206, 17331763, 17335380, 17336979, 1733740, 17339329, 17339959, 17339967, 1734011, 17342593, 17346164, 17346813, 17349, 17356340, 17357900, 17358042, 17358124, 17358278, 17359268, 1735971, 17359919, 17360927, 17361358, 17361569, 17362087, 17362508, 17365096, 17366348, 17367951, 17368550, 17368839, 17368894, 17369628, 17372964, 1737371, 17374849, 17376703, 17381046, 17381349, 17381850, 17382152, 17384514, 17387017, 17387308, 1738737, 17387606, 17392942, 17394218, 17394539, 1739618, 17404880, 17405348, 17405356, 17408253, 17408262, 17411460, 17412464, 17413467, 17417772, 17424715, 17424934, 17425155, 17426592, 17428013, 17436023, 17436330, 17438701, 17441552, 1744158, 17442764, 17443782, 17445368, 17445407, 17447000, 17449035, 17454352, 17457421, 17465561, 17466389, 17471562, 17476202, 17481522, 17483461, 1748408, 17484228, 17484414, 17485418, 17488272, 17492279, 17492366, 17500799, 17501272, 17502109, 1750445, 17504507, 17505854, 17507250, 17509085, 1750955, 17510752, 17511158, 1751956, 17522080, 17524254, 17526503, 17531537, 1753237, 17535920, 17539707, 17540445, 17546271, 17549522, 17555122, 17555765, 17556503, 17562065, 17562860, 17564509, 17572459, 17574378, 17575156, 17575302, 17576066, 17576893, 17577146, 1757722, 17577631, 17578108, 17579481, 17586020, 17586427, 175868, 17590477, 17594050, 17596339, 176009, 17601608, 17602966, 17603785, 1760559, 17606521, 17607919, 17613547, 1761697, 17617025, 17617929, 17620332, 17626720, 17627674, 17630767, 17630992, 1763244, 17633899, 17635181, 17638027, 17639199, 1764199, 17645907, 17646909, 17652404, 1765247, 17654596, 17655274, 17656090, 17657983, 17660276, 17660745, 17661465, 17661867, 17663387, 17665597, 17665809, 17666070, 17666887, 17669549, 17671012, 17671213, 17671553, 17671571, 17672057, 17672308, 17673569, 17674752, 17686359, 17691082, 17692199, 17693547, 17697976, 17698631, 17699360, 17700651, 17701720, 17702903, 17703393, 17703616, 17707025, 17707981, 17708473, 17709080, 17712285, 17717982, 1771834, 17718857, 17719847, 17720481, 17720566, 17721497, 1774725, 17748756, 17754139, 17760545, 17760977, 17762169, 17762199, 17766102, 17767200, 17774003, 17774791, 17785648, 17788491, 17802357, 17805079, 17810293, 1781781, 17821369, 1782686, 17827386, 17827881, 1782920, 1784562, 17851574, 17852353, 17854850, 17855577, 17870039, 17872615, 17876835, 17877642, 17878714, 17879205, 178804, 17883097, 17885498, 17886369, 17887732, 17888651, 17889514, 17894484, 17905709, 17906085, 17908360, 17910385, 17910600, 17911188, 17913404, 17913621, 17913827, 1791911, 17921930, 17924437, 17924472, 17926835, 1792697, 17928564, 17932248, 17932285, 17932727, 17935366, 17936103, 17938748, 17940103, 17940123, 17945709, 17947195, 17947483, 17950040, 17955250, 17956960, 17958394, 17959957, 17960283, 17960486, 17962014, 17966168, 17967332, 17967626, 17967701, 17969017, 17969033, 17970227, 17975005, 1797548, 17981598, 17983275, 17983402, 17983566, 17987060, 17988511, 17989375, 17995184, 17997600, 17998606, 17999222, 18000655, 18005210, 18019146, 18023236, 18023632, 18028607, 18034012, 18034824, 18041707, 18042555, 18043142, 18045444, 18053976, 18056386, 18057701, 1805793, 18058850, 18059748, 1806339, 18064291, 18064474, 18069332, 1807053, 18073272, 18073522, 18078941, 18082990, 18084038, 18084090, 18085751, 18086302, 18086752, 18087706, 18091261, 18093007, 18093984, 18095760, 18096060, 18096285, 1812767, 1813810, 1815219, 18154181, 18155254, 18156793, 18156937, 18157245, 18157458, 18159098, 18164546, 18172127, 18176778, 18180385, 18182451, 18183225, 18183347, 18184609, 18184763, 18189274, 18192357, 18193427, 18195780, 18198261, 18201930, 1820277, 18203635, 18204046, 18206463, 18212427, 18215930, 18217334, 1821802, 18218501, 18218522, 18218545, 18219817, 18220732, 18222642, 1822269, 18226120, 18231821, 18232615, 18236682, 18237331, 18239904, 18244636, 18247233, 18255010, 18255148, 18257295, 18258664, 18259968, 18261721, 18262774, 18265023, 18267051, 18270818, 18272602, 18272864, 18278213, 18278561, 18280409, 18281705, 1828175, 18286812, 1829368, 18294385, 18295114, 18296352, 18299081, 1830127, 18301529, 18302616, 18305764, 18307840, 18309370, 18310752, 18311288, 18311771, 18311983, 18313930, 18315238, 18316581, 1831967, 1832023, 18325721, 18328430, 18328590, 18332048, 18335255, 18338050, 18339411, 18343150, 183441, 18351458, 18356001, 18356377,	

		18361062, 1836108, 18362915, 18367067, 18367957, 18368510, 18374161, 18375176, 18375796, 18376074, 18377068, 18378046, 18385592, 18386813, 18386998, 18387998, 18388309, 18390653, 18390705, 18390967, 18392559, 18393492, 18394568, 18395150, 18398334, 18400524, 18401174, 184019, 18402068, 18403987, 18406069, 18408790, 18410229, 18410497, 18411147, 18411578, 18412306, 18413949, 18417133, 18419748, 18421455, 18422390, 18424592, 18425228, 18426215, 18427651, 18428355, 18429671, 18434503, 18435420, 18437359, 18439727, 18441028, 18441325, 18441957, 18442089, 18446422, 18449291, 18449865, 18450050, 18451615, 18452779, 18453634, 18457813, 18458579, 1846030, 18462268, 18466695, 18466742, 18468251, 18469377, 18469544, 18473703, 18479589, 18483611, 18485034, 18485693, 18486098, 18486516, 18487394, 18487658, 18490431, 18494590, 18495057, 18495610, 18498124, 18499224, 18501667, 18504234, 18509063, 1851355, 18515168, 18516246, 18518051, 18519648, 18523497, 1852795, 18535721, 18536920, 18540292, 18540800, 18541229, 18543902, 18547020, 18547132, 18548090, 18551679, 18552234, 18555788, 18556020, 18556436, 18559927, 18561088, 18561734, 18563738, 18566424, 18567657, 1856813, 18568865, 18570563, 18576061, 18577364, 18579422, 18583515, 18584135, 18585175, 18586297, 18588123, 18588335, 18589196, 18591144, 18592700, 18593610, 18597480, 18600051, 18601130, 18601505, 18602630, 1861112, 18614178, 18616526, 1862358, 18625522, 18627861, 18633284, 18633319, 18635821, 18639579, 18640097, 18643320, 1864757, 18648743, 18649340, 18650607, 18653730, 18654924, 18656291, 18665619, 18666580, 18681956, 18682558, 18682703, 18683735, 18686707, 18687303, 18687385, 18691053, 18691600, 18692081, 18692276, 18693089, 18694021, 18694270, 18695514, 18695717, 18702340, 18702984, 18703439, 18704484, 18704675, 18705469, 18708870, 18710147, 18711299, 18714341, 18722636, 18725336, 18726555, 18728004, 18729242, 1874788, 18755162, 18756430, 18758484, 18758489, 18761081, 18764819, 18769831, 18770134, 18770298, 18770474, 18772674, 18773917, 18775324, 18776525, 18779933, 18781933, 18784833, 1878525, 18789794, 18789815, 1879141, 1879220, 18796623, 18797935, 18797959, 1880175, 18802123, 18803834, 18804346, 18806135, 1880702, 18807058, 18809134, 18810789, 18810841, 18818276, 18818572, 18819204, 18819357, 18821986, 18823001, 18823039, 18828012, 18828120, 18830920, 18832852, 18836449, 18837047, 18842002, 18843001, 18844528, 18846561, 18850256, 18852082, 1885372, 1887700, 1891501, 18922041, 18923813, 18926862, 18928113, 18928752, 18929025, 1893608, 18936492, 18937650, 18938107, 18948850, 18951012, 18951453, 1895292, 18954875, 18960954, 18961538, 18963870, 18964304, 18966045, 18968009, 18968517, 18968753, 18969391, 18969716, 18978678, 189813, 1898262, 18987587, 18988088, 18988747, 18989053, 18989093, 18991111, 1899185, 1899637, 18996467, 18996898, 19003264, 19007388, 19007864, 19009754, 19011208, 19012174, 19012715, 1901724, 19018804, 19020345, 19021537, 19022297, 19026775, 19029882, 19036985, 19037964, 19040980, 19042478, 19043393, 19043919, 19045828, 19046216, 19050254, 19059790, 19060602, 1906073, 19065367, 19069848, 19072009, 19073232, 19073853, 19074623, 19082115, 19082270, 19082718, 19083376, 19087010, 19089839, 19090048, 19090865, 19092346, 1909383, 19095112, 19101318, 19102184, 19104456, 19108488, 19111463, 19111970, 1911556, 19117200, 19120966, 19121686, 19123959, 19126778, 19129126, 19135693, 19138066, 19140240, 19147015, 19150153, 19153042, 19153559, 19156629, 19158551, 19162795, 19163570, 19163869, 19164991, 19168964, 19171355, 19172223, 19174422, 19175528, 19176946, 19178758, 19180242, 19184626, 19184997, 19185567, 19189205, 19190061, 1919144, 19192409, 1919297, 19193637, 19193726, 1919688, 19197982, 1920404, 19204456, 19208177, 19210873, 19212517, 19215299, 19216557, 19224300, 19225212, 19225875, 19227323, 19227752, 1923365, 19235136, 19235956, 19237993, 19241354, 19243390, 19243878, 19246867, 19250220, 1925107, 19259400, 19260501, 19264168, 19264348, 19264532, 19267029, 19271413, 19273179, 19276285, 19277013, 19277359, 19279767, 19283475, 19283824, 19284115, 19286449, 19289507, 19291216, 19291685, 1929674, 19300514, 19302231, 19302302, 19302701, 1930396, 19308775, 19308950, 19317371, 19317835, 19318353, 19326965, 19331901, 1933435, 19337152, 19338710, 1933922, 19345404, 19349185, 19349618, 19355934, 19356869, 19357805, 19359864, 19363978, 19369731, 19375790, 19378003, 19378894, 19379927, 19380467, 19381774, 19383060, 19385288, 19395910, 19399667, 19403342, 19403664, 19407935, 19409348, 1941048, 19410627, 19418568, 19418953, 19419498, 19420057, 19420589, 19420947, 19422847, 19423405, 19427334, 19427356, 19427409, 19429119, 19429551, 19431700, 19437829, 19437929, 19439555, 19440144, 19447626, 19450711, 19462435, 19467487, 19470614, 19471547, 19477942, 1947820, 19479071, 19479968, 19481530, 19483209, 19484288, 19486035, 19486191, 1948643, 19487732, 19490513, 19493203, 19494626, 19498867, 1949887, 19505649, 19508305, 19508530, 19508829, 19515327, 19517408, 19519820, 19519897, 19522217, 19524403, 19525268, 19527537, 19528135, 19529993,	

		19530071, 19534109, 19534530, 19535636, 19536742, 19542009, 19545810, 19546346, 19546832, 19549071, 19549305, 19549582, 19549748, 19552540, 19553482, 19554180, 19555120, 19557792, 19560253, 19565023, 19567730, 19568312, 19573328, 19573668, 19573754, 19574155, 19574908, 19575463, 19576088, 1958023, 19580964, 19582459, 19588049, 19594822, 19595304, 19602308, 19603963, 19607869, 19609866, 1961122, 1961388, 19618569, 19620192, 19621119, 19622536, 19625391, 19627114, 19627484, 19627497, 19627712, 19629866, 19629914, 19629972, 19631827, 19632475, 19635874, 19637992, 19643847, 19643852, 19645022, 1964556, 19649250, 19649658, 19652501, 19653642, 19656816, 19660547, 19663590, 19663805, 19664682, 19665072, 19666694, 1966782, 19668151, 19673432, 19674189, 19675987, 19678996, 19679377, 19682999, 19683045, 19683576, 19691976, 19696881, 19700643, 19700873, 19704155, 19705949, 19706229, 19712760, 19712882, 19714954, 19715087, 19717202, 19719477, 19723104, 19724143, 19725725, 19727334, 19731195, 19731204, 19731587, 19734060, 19739672, 19740692, 19742244, 19743401, 19744119, 19744257, 19744862, 19745017, 19747594, 19758925, 19760514, 19763989, 1976531, 19767416, 19768853, 19772199, 19776259, 1977799, 19779305, 19781418, 19782067, 19784679, 19784787, 19792546, 19792795, 19800990, 19801208, 19801934, 19803965, 1980453, 19808243, 1981057, 19815342, 19816512, 19816701, 19818210, 19820936, 19821528, 19825897, 19826542, 19829801, 19830549, 1983165, 19832035, 19833400, 19836316, 19836755, 19837518, 19837590, 19837732, 1984329, 19843528, 19844387, 19847865, 19853941, 19855779, 19857161, 1985822, 1986149, 19861952, 19862748, 19863582, 19865103, 19872284, 19874203, 19875747, 19876318, 19877672, 1987988, 19881105, 19891823, 19894312, 1989519, 19897374, 19897678, 19897834, 19899529, 19899930, 19901417, 19901694, 19902605, 19902810, 19906864, 19911748, 19914560, 19918549, 19926129, 19927586, 199283, 19929069, 19929291, 19930072, 19933366, 19937606, 19937982, 19939484, 1994830, 19950330, 19954994, 1995642, 1995766, 19958014, 19959107, 19960699, 19962714, 19963886, 19963949, 19966109, 19966272, 1997889, 1999197, 19996632, 19997756, 19998810, 19999970, 20004471, 20007633, 20008431, 20009207, 20010146, 20013024, 20024341, 20024413, 20026269, 20026903, 20027364, 20027581, 20035646, 20036634, 20036757, 20038029, 2004222, 20044170, 20047134, 20047671, 20052653, 20052679, 20054405, 20056307, 20060089, 20062731, 20066250, 20066325, 20066395, 20066989, 20069843, 20070558, 20071596, 20072727, 20072900, 20074878, 200784, 20083308, 20086475, 20086838, 20086979, 20087044, 2008727, 2009269, 20102922, 20107640, 20107662, 2011072, 20113509, 20117976, 20122021, 20123451, 20127017, 20127125, 20129800, 20131403, 20133048, 20134074, 20135182, 20136451, 20138411, 20140787, 20141292, 20141496, 20145303, 20146547, 2014892, 2015005, 20154710, 20161521, 2016321, 20163415, 20164930, 20166904, 20167223, 20167379, 20169949, 20173597, 20175627, 20178622, 20179397, 20182762, 2018385, 20184321, 2019177, 201922, 20192817, 20195504, 20198268, 20199442, 20203347, 20204534, 20205757, 20206222, 20210498, 20212825, 20213324, 2021427, 20214573, 20216635, 2022837, 20229008, 20233676, 2023435, 20234873, 2023918, 20297892, 2029816, 20304754, 20307619, 2031458, 20329854, 20335958, 20337349, 2034226, 20347344, 20347655, 20347787, 20349394, 20351055, 20351194, 20351199, 20355800, 20356377, 20358147, 20361723, 20363044, 20363500, 20365203, 20366047, 20368037, 2036958, 20370568, 20370943, 20371105, 20371395, 20373236, 20374940, 20375556, 20377392, 20379374, 20380017, 20383274, 20384302, 20384474, 20384574, 20384813, 20388849, 20389396, 20389466, 20389664, 20390578, 2039336, 20397272, 2039744, 20397578, 2039770, 20398480, 20398927, 20399486, 20400509, 20400740, 20401255, 20403389, 2040523, 20411258, 20416942, 20422097, 20423141, 20423761, 20425227, 20426334, 20426353, 20428582, 20438325, 20438642, 2044266, 2045380, 20457868, 20458644, 20458741, 20458890, 20464219, 20464393, 20469747, 20471028, 20473380, 20473434, 20477508, 20477549, 20478142, 20478233, 20478395, 20491067, 20491210, 20491497, 20492119, 20492829, 2049671, 20496766, 20497745, 20502513, 20509759, 20510455, 20515766, 20517678, 20519181, 2051923, 20523042, 20523729, 2052391, 20524994, 20531480, 20533914, 20534671, 20536627, 20536931, 20538010, 205386, 20539152, 20541512, 20542298, 20542996, 20543897, 20545595, 20547153, 20549470, 20550105, 20550380, 20551791, 20551999, 20553072, 205535, 20556003, 20556571, 20557505, 20557618, 20561157, 20562296, 20566579, 20567670, 20567763, 20569097, 20569725, 20570254, 20575856, 20576438, 20577466, 2057868, 20580947, 20584708, 20587955, 20589243, 20589841, 20593271, 20593588, 20597094, 205983, 2060266, 20605846, 2060615, 20609378, 2061124, 20614261, 20615794, 20616380, 20616988, 20618307, 20620143, 20620267, 20620930, 20622361, 20626250, 20628331, 20630818, 20634125, 20635160, 20635403, 20637459, 20637809, 20637847, 20638613, 20638839, 2063994, 20640482, 20642948, 20643792, 20646293, 20650894, 20651910, 20652642, 20655152, 20656971, 20658204, 20660213, 20660675, 20661527, 206630, 20663770,	

		20665325, 20674108, 20677579, 20680129, 20681389, 20684153, 20684975, 2068595, 20686924, 20688175, 20690005, 2069136, 20694678, 20699065, 20700292, 20701630, 20701728, 20702350, 20702934, 20704259, 20709613, 2071231, 20712732, 20717361, 20718290, 20720408, 20722426, 20722947, 2072295, 20724058, 20725529, 20728517, 20730609, 20731342, 2075298, 2075485, 207898, 20795735, 20797850, 20801691, 2080326, 20805525, 20806049, 20807621, 20810927, 20811134, 20812912, 20814349, 20816412, 20816837, 2081717, 20820417, 20820759, 20824431, 20825491, 20826699, 20827438, 20829912, 20833213, 20833938, 20835918, 20839241, 20839470, 20839920, 20846170, 20848794, 20849585, 20854073, 20856456, 2085745, 20859782, 20862095, 20862183, 20865365, 20866397, 20866493, 2086763, 20869177, 20872664, 20876087, 2087933, 20880884, 20881902, 20884355, 20884587, 20886393, 20886821, 20888606, 20889911, 20890105, 2089376, 2089965, 2090981, 20921203, 20922254, 20929238, 20929974, 20930913, 2093320, 20938692, 20942238, 20942483, 20942728, 20943819, 20951290, 20955374, 20957546, 20959091, 20959291, 20967271, 20969767, 20975528, 20977192, 2097829, 2098421, 2098987, 2099526, 21031634, 21033147, 21035156, 21036436, 21041619, 21042363, 21042610, 21042753, 2104393, 210448, 21054369, 21056028, 21056431, 21058047, 2105895, 21064013, 21068290, 21071120, 21075672, 21080788, 21082201, 21084399, 21085526, 21085816, 21087158, 21087336, 21087597, 21088604, 21090855, 21093426, 21096503, 21103140, 21108101, 21110900, 21111721, 2111731, 21118468, 21119457, 21121794, 2112217, 21122449, 21124668, 21124689, 21124693, 21129030, 21130843, 21131426, 21131657, 21135361, 21137361, 21139861, 21141559, 21147141, 21149986, 21151033, 21151039, 21153001, 2115484, 21154889, 21156111, 2115655, 21160352, 2116331, 21167241, 2117499, 21175498, 21175872, 21176481, 21177698, 2118106, 21182394, 21183982, 21184652, 21185115, 21194218, 21194429, 2119445, 21200204, 21200841, 21202591, 21205496, 21205840, 21210345, 21214481, 21215824, 21220750, 21222298, 21225392, 21228222, 21231591, 2123495, 21235876, 21236957, 21239226, 21240622, 21241259, 21244731, 21246048, 21248726, 21249674, 21251994, 21255143, 21257697, 21258749, 21262582, 21262744, 21263250, 21266044, 21267507, 21269955, 21271105, 21272477, 21273315, 21273760, 21275520, 21277913, 2127826, 21280839, 21281709, 21291855, 21293872, 21295518, 2129600, 21298251, 2130143, 21304137, 21306938, 21311700, 2131353, 21316131, 21316171, 21319257, 21320038, 21321694, 21323801, 21323999, 21327915, 21335238, 21337044, 21337316, 21338334, 21338958, 21340364, 21341244, 21344028, 21344998, 21347136, 21347410, 21348389, 21348420, 21353489, 2135453, 21355013, 21356613, 21356951, 21360747, 21362197, 21365827, 21367486, 21369007, 21369918, 21371545, 21378150, 21382522, 21384261, 21385236, 21387119, 21388080, 21389057, 2139030, 21390342, 21391237, 21391641, 21391686, 2139406, 2139445, 21396388, 21399280, 21401958, 21402456, 21403791, 21404116, 21405542, 21413046, 21413525, 2141359, 21414720, 2142017, 21420683, 21422087, 2142253, 21422632, 21424048, 21429530, 21432834, 21440018, 2144006, 21449675, 21451732, 21455087, 21455988, 21456715, 21457148, 21460834, 21462012, 2146449, 21464540, 21476882, 21479156, 21480746, 21481198, 21484568, 21489253, 2148977, 21491198, 21493312, 21497012, 21499416, 21501641, 21503423, 21508868, 21511957, 21514734, 21519717, 21522399, 21522828, 21522907, 21525386, 21525929, 21527203, 21528313, 21531214, 21532221, 21534506, 21534588, 2153488, 21536207, 21537683, 21543160, 215444, 21555367, 21557972, 2155901, 21559639, 21559785, 21563392, 21566477, 21569815, 21570673, 21570802, 21574688, 21577164, 2157848, 21580550, 21580769, 21580986, 2158104, 21581783, 21589016, 21590774, 21596326, 21596703, 21596803, 2160101, 21607579, 21614741, 21621409, 21621502, 21636497, 21636706, 2163793, 21638537, 21641715, 21642717, 21645789, 2164915, 2164917, 21650173, 21656224, 2166276, 21664595, 21669785, 21676505, 21676607, 21677146, 21679247, 21679894, 21680367, 21682734, 21687596, 2168780, 21688273, 21689063, 21693070, 21693566, 21695525, 21696738, 21700332, 21700424, 2170181, 21702505, 21703032, 2170474, 21708830, 21709196, 21712097, 21712475, 21714456, 21718789, 21719834, 21723658, 21725623, 21726310, 21726377, 21726612, 21727904, 21733518, 21736372, 21736796, 21739239, 21740363, 21741649, 2174470, 21744892, 2174598, 21748065, 21753209, 21754002, 21754500, 21756078, 21757324, 21762857, 21765074, 21767177, 21773608, 21773863, 21773917, 21774970, 21777432, 21780059, 21781062, 21781256, 21784329, 21785588, 21786998, 21787338, 21787643, 21787974, 21790621, 21792555, 21795433, 21796044, 2179625, 2179631, 21797127, 21800175, 21801369, 21801703, 21803473, 21805388, 2180697, 21811262, 21811738, 21813486, 21813991, 21815389, 21816142, 21816346, 21819045, 21821070, 21822831, 21824553, 21825349, 21827977, 21829566, 21831653, 21834119, 21838819, 21839992, 21842408, 21844973, 2184927, 21855684, 21859343, 21859438, 21861505, 21861510, 21864358, 21866999, 21869143, 2187097, 21870971, 21874433, 21877751, 21882228, 21883272, 2188969, 21892478, 21898944, 21902709, 2190554, 2190921, 21911348,	

**True Negative**	None	21914085, 21914703, 21919687, 21921070, 21925092, 21937722, 21941914, 21942702, 21947565, 21952289, 21953708, 21953815, 2195700, 21959843, 21961712, 21964557, 21967294, 21967494, 21970414, 21971732, 21975277, 21978926, 21982102, 21982124, 21986663, 21993859, 21997404, 2200192, 22003739, 22004902, 22013182, 22015445, 22016549, 2201848, 22021004, 22022906, 22023476, 22023720, 22023880, 22029939, 22031074, 22035178, 22037248, 22037913, 22039064, 22039957, 22040101, 2204348, 2204352, 22043588, 22045240, 22049175, 22049826, 2206007, 22062193, 22064753, 22066278, 22067089, 22068036, 22068383, 22069685, 22071686, 2207394, 2207602, 22076288, 22077770, 22081691, 22082160, 22083312, 22087726, 22091331, 22093310, 22099967, 2210158, 22103737, 2210658, 22107108, 22108389, 22113082, 22114161, 22122282, 22123168, 22125586, 22125994, 22127766, 22128990, 22131390, 22134448, 2213505, 22140155, 22146008, 22149727, 22150686, 22151330, 22151697, 22164461, 22164628, 22164783, 22167042, 22167629, 22171108, 22173006, 22178394, 22179804, 22183265, 22185449, 22185560, 22195267, 22197543, 22197734, 22198226, 2220129, 22203410, 22205651, 2220719, 22207231, 22209602, 22210534, 22212204, 22212649, 22218299, 22222165, 22225472, 2222649, 22227235, 22227850, 22228717, 22229083, 22230933, 22233441, 22239618, 22241158, 2224304, 22243313, 22243719, 22247910, 2224904, 22253476, 22255036, 22256538, 2225710, 22261143, 22262727, 22263050, 22265774, 22265963, 22269013, 22270410, 22273884, 22275144, 2228415, 22286783, 22290408, 22291572, 2229572, 22296208, 2229917, 22299717, 22300950, 22303812, 22305276, 22308332, 22311008, 22311141, 22311479, 22314130, 22316392, 22323364, 22323577, 22330066, 22330450, 22330489, 2233378, 22339671, 22341871, 22341941, 22342328, 22342599, 22343107, 22345089, 22348819, 2235303, 22353687, 22362364, 22366397, 22369589, 22372390, 22376269, 22377309, 22379034, 2238098, 22381900, 22388500, 22389543, 22392605, 22398026, 22402836, 22406623, 22407347, 22408169, 224087, 22409599, 22417526, 22417682, 22418139, 22419286, 2242634, 22427189, 22433052, 22434343, 22438661, 22440172, 2244032, 22440745, 22442674, 22443696, 22444181, 22446018, 22448452, 22451576, 22452570, 22453505, 2245371, 2245494, 22455729, 22457622, 22459330, 22461614, 22461812, 22463980, 2246447, 22464945, 22473186, 22474418, 22475452, 22477095, 2247729, 22479501, 2248047, 22481791, 22482366, 22482932, 22486679, 22490823, 22491552, 2249205, 22492141, 22497610, 22500063, 22501065, 22502766, 22503838, 22504569, 22505860, 22507526, 22507601, 22507866, 2251432, 22514762, 22515891, 22516959, 22517994, 22523511, 22523972, 22526429, 22526494, 22527791, 22529870, 22530490, 22533458, 22535195, 22540282, 22540467, 22551888, 22552206, 22564611, 22573615, 22579694, 22581860, 22582334, 22584057, 22585639, 22586090, 22588065, 22590168, 22590361, 22591224, 2259279, 22596255, 2259789, 22607017, 22608990, 2261046, 22612021, 22614588, 22616524, 22616601, 22617814, 22624072, 22632823, 22633861, 2263515, 22637968, 22642541, 2264498, 22645461, 22650311, 226576, 22661912, 22662075, 22668525, 22675073, 22677433, 22679237, 22681545, 22682620, 22682789, 22686063, 22686203, 22690191, 22692536, 22693572, 22693875, 22697395, 2270141, 22702192, 22706747, 22708492, 22709547, 22710827, 22713253, 22713765, 2272254, 22723775, 22724584, 22728133, 22728637, 22730656, 22737613, 22738940, 22743018, 22743957, 22745764, 22749760, 22749952, 22753094, 22761984, 22768014, 22770592, 2277158, 22775566, 22777156, 22778045, 22778469, 22778833, 22779756, 22782527, 22791504, 22797192, 22798526, 22804106, 22804986, 22809244, 22809246, 2281206, 22812389, 22812603, 22814213, 22815713, 22818535, 22820045, 22820451, 22820504, 22820545, 22820747, 22821530, 22824473, 22825495, 22830254, 22832333, 22833406, 22837534, 22842744, 22843170, 22846401, 22850192, 22855313, 2286620, 22866931, 22869759, 22870708, 22872479, 22874249, 22876664, 22877886, 22878191, 22880014, 22880838, 22881987, 2288382, 2288728, 22888405, 22889609, 22889880, 22898245, 22899821, 22900001, 22906708, 22911291, 22911702, 22912818, 22913294, 22916316, 22919773, 22920884, 22925225, 22925396, 22926326, 22930280, 22931607, 22933044, 2293319, 22934045, 22934186, 22941794, 22942363, 22945586, 22948092, 22952321, 22953449, 2295355, 22955224, 22956542, 22957631, 22958287, 2296035, 22961453, 22966163, 22969329, 22969705, 22970505, 22971639, 22971860, 22975713, 22975962, 22976388, 22977867, 22978013, 22985413, 2298910, 22993201, 22994708, 22994948, 22995182, 23002361, 23002813, 23003113, 23007338, 23009289, 23010283, 23013700, 23014769, 23015879, 23016397, 23019917, 23020696, 23025902, 23026004, 23026974, 23027770, 23028410, 23033238, 23036130, 23039745, 23041058, 23042005, 23043117, 23044359, 23044539, 23047013, 23049847, 23053541, 23054840, 23056022, 2305982, 23061787, 23063834, 23064544, 23077306, 23078474, 23078764, 23083147, 23084208, 23086953, 23088863, 23091626, 2309265, 23097674, 23104179, 23106504, 23109773, 23111241, 23113015, 23113339, 23116117, 23116382, 23116853, 23120697, 23122695, 23125589, 2312776, 23128310, 23128370, 2312874, 23129230, 2313297, 23133383, 23133400,	27792997,

			23134934, 23138103, 23139605, 23143281, 23144030, 23149037, 23149731, 23152898, 23153411, 23155495, 23158208, 23164441, 23166639, 23167968, 23168777, 23169773, 23173189, 2317522, 23178940, 23179111, 23182463, 2318561, 23186564, 23187835, 2319204, 23192545, 23194212, 23195242, 23199676, 23199824, 23201626, 23201797, 23207497, 23208250, 23210258, 23211929, 23213979, 23214599, 23219798, 23221142, 23221864, 23222908, 23226928, 23227260, 23227628, 23229429, 23230658, 23231845, 23237698, 23237907, 23239198, 2324134, 23241665, 2324284, 23246431, 23248358, 23249176, 2325232, 23255439, 23256161, 23259089, 23264900, 23265669, 23266365, 23266476, 23270085, 23273611, 2327472, 2328068, 23283228, 23284520, 23289416, 23291247, 23299224, 23302988, 23303925, 23308312, 2331243, 23313708, 23315699, 23317708, 23320062, 23323043, 23323466, 23328042, 23329855, 2333049, 23335982, 23337481, 23338120, 23342507, 23345679, 23346129, 23349662, 2335034, 23350971, 23351489, 2335434, 23355455, 23355756, 23361060, 23362319, 23362478, 23363618, 23363643, 23363947, 23364080, 23368143, 23368929, 23371249, 23373816, 23375510, 23378533, 23382305, 23382995, 23383502, 23393376, 23393836, 23398773, 23400426, 23406545, 23408601, 23414270, 23415083, 23415598, 23416910, 23419839, 23419872, 23420010, 23420565, 234210, 23422355, 23423597, 23425123, 23425612, 23427432, 23428066, 23428519, 23430397, 23434196, 23435942, 23437130, 23437318, 23438768, 23439297, 23440011, 23441629, 23447133, 23449039, 23449130, 23449175, 23450332, 23451763, 23453545, 23454520, 23454782, 23454904, 23455438, 23456203, 23459131, 23460815, 23461271, 23469022, 23469789, 23473766, 23474220, 2347488, 2347582, 23478900, 23480487, 2348083, 2348302, 23486285, 23486441, 23493858, 23494412, 23495637, 23497001, 23497808, 23498765, 23498904, 23499613, 23501835, 23501940, 23501944, 23512584, 23515235, 23515373, 23519531, 23520215, 23524154, 23524701, 23525529, 23529911, 23530810, 23531031, 2353318, 23534222, 23534393, 23535916, 23537, 23537842, 23539913, 23542116, 23543886, 23548184, 23554626, 23555318, 23556144, 23556597, 23559075, 23560560, 23560698, 23563160, 23563753, 2356443, 23565418, 23569594, 23571094, 2357281, 23574872, 23576741, 23579574, 23579802, 2358395, 2358521, 23587449, 23588232, 23589863, 23590732, 23591204, 2359175, 23595519, 23596348, 23596757, 23599101, 23599638, 23601222, 23603431, 23603755, 23605372, 23606436, 23608978, 23609719, 2361066, 23611543, 23612878, 23613412, 23619818, 23622723, 23622749, 23624649, 23626680, 23629684, 23635390, 23642085, 23652537, 23656914, 23658250, 23658456, 23658858, 23669599, 23669974, 23673529, 23675770, 23677267, 23678667, 23679662, 23680277, 23684079, 23689894, 23691323, 23696706, 23702420, 23702807, 23705594, 2371245, 23716210, 23716227, 23721583, 23722916, 23725457, 23725858, 23726220, 23726344, 23729997, 23733243, 23734935, 23737179, 23738058, 23738124, 23740003, 23742700, 23744452, 23746324, 23747457, 23747645, 23749258, 2376091, 23761623, 23762374, 23763191, 2376650, 23766569, 23766959, 23770395, 23774085, 23775386, 23775999, 23777456, 23777537, 23779226, 23783458, 2378657, 23801764, 23803249, 23803430, 23804088, 23806237, 23806502, 23811922, 23814611, 23821599, 23824770, 23829793, 23831795, 2383291, 23833557, 23835048, 23837060, 23837604, 23838558, 23841004, 23843185, 23845027, 23846859, 23849147, 23851095, 2385166, 23852539, 23856000, 23856378, 23856765, 23867537, 2386870, 23869243, 23869547, 23871690, 23871695, 23876573, 23876932, 23879006, 23880034, 23880398, 23883401, 23886669, 23888245, 23889307, 23896277, 23896763, 23897109, 23898948, 23905443, 23907735, 23910079, 23910879, 23916001, 23920044, 2392093, 23920940, 23922482, 23923631, 23924575, 23925125, 23925703, 23927277, 23927829, 23930200, 23932656, 23932771, 23935967, 23942801, 23945562, 23946147, 23949337, 23955745, 23958584, 2396343, 23966880, 23967944, 23972903, 23974112, 23974562, 23979060, 23979546, 23982340, 23982427, 2398246, 23982679, 23984960, 23985452, 23985903, 23988790, 23992861, 23994782, 23995744, 23996171, 23997826, 23998101, 24002235, 24003589, 24005414, 24006877, 24010405, 24019790, 24020032, 24020232, 24021298, 24023376, 24029597, 24030213, 24031294, 24032302, 2404050, 24041722, 24045639, 24047049, 2404715, 24049923, 24050954, 24052979, 24054484, 24055465, 24062834, 24063369, 24063620, 24067909, 24069852, 24072877, 2407347, 24073960, 24076513, 24077756, 2407911, 24080823, 24090213, 24091976, 24094635, 24098012, 24101457, 24102848, 24108551, 2410943, 24109859, 24113197, 24113728, 24119385, 24119474, 24121502, 24122178, 24122626, 24124968, 24127311, 24130099, 24130749, 24132456, 24133332, 24135845, 24137388, 24138021, 24141084, 2414273, 24144157, 24144251, 24149240, 24153155, 24154725, 24154826, 24157573, 2416079, 24162234, 24164818, 24166503, 24167739, 2416906, 24173563, 24174315, 24174900, 24179411, 24179491, 24184420, 24186191, 24187295, 24190468, 2419566, 24198366, 24198967, 24203469, 24205917, 24207158, 24211295, 24212492, 24213283, 24215213, 24215917, 24217409, 2421855, 24218929, 24219634, 24220179, 24220293, 24223041, 24223796,

		24225737, 24226604, 24226918, 24227912, 24231801, 24232879, 24233071, 24237181, 24238319, 24239415, 24246555, 24248198, 24249229, 24252570, 2425530, 24257775, 2426350, 24265112, 24266811, 24267444, 24273634, 24275060, 24276038, 24279400, 24279969, 24282378, 24291151, 24292814, 24293464, 24295414, 24296331, 24297600, 24301799, 24302122, 2430287, 24304626, 24305940, 24306618, 24307048, 24314301, 24314360, 24314367, 24314620, 24315801, 24319117, 24319684, 24320327, 24321689, 24324923, 24327547, 24330637, 24330864, 24332793, 24342833, 24353633, 24354169, 2435438, 24355917, 24356218, 24356945, 24357144, 24360827, 24367425, 24368593, 24370432, 24377447, 24379523, 24382391, 24385215, 24393755, 24395244, 24400777, 24402079, 24404536, 24406401, 24407134, 24407525, 24408311, 24409109, 24409315, 24413546, 24415522, 24419499, 24424165, 24424232, 24424714, 24427177, 24427772, 24428291, 24430402, 2443221, 24434323, 24434759, 24436669, 24437742, 2443860, 24439047, 2443997, 24442268, 24442910, 2445339, 24457551, 24459152, 24460096, 24461137, 24464708, 24466377, 24469040, 24469254, 24469724, 24477261, 24483091, 24484191, 2448426, 24484877, 24486891, 24488979, 24492092, 24494865, 24495919, 24499056, 24500721, 24503859, 24504155, 24504899, 24505529, 24506367, 24516497, 24518221, 24527217, 24529954, 24530416, 24531622, 24532720, 24533743, 24534025, 24535316, 24547620, 24548316, 24549683, 24553673, 24554615, 24555107, 24556421, 24556826, 24557887, 24558265, 2455871, 24559681, 24559717, 24559770, 24566556, 24568117, 24568184, 24576612, 24579406, 24580450, 24582690, 24588146, 24588465, 24592050, 24596624, 24597943, 24599915, 24601036, 24602732, 24605431, 24608216, 24612607, 24613364, 24614746, 24615252, 24615530, 24619223, 24622350, 24624320, 24630244, 24630831, 24631471, 24632060, 24633179, 2463565, 2463973, 24644055, 24647651, 246496, 24652277, 24654370, 24655901, 24659692, 24661540, 2466671, 24668157, 24671002, 24673711, 24676585, 24678952, 24681317, 24682985, 24683156, 24683502, 24694419, 24699694, 24699979, 2470025, 24707166, 2471293, 24714015, 24714030, 24718917, 24721682, 24726311, 24729866, 24738583, 24740255, 24740904, 24742064, 24743080, 24749015, 24749308, 24752330, 24763274, 2476657, 24768206, 2476897, 24772231, 24777145, 24778473, 24778570, 24779895, 24780421, 24782510, 24783246, 24783784, 24784301, 24785106, 24790623, 24791729, 24791778, 24792990, 24794130, 2479587, 24796662, 24798257, 24799815, 24799889, 24801803, 24802409, 24802489, 24803386, 24803769, 24804003, 24808909, 24813710, 24813827, 24815027, 24822232, 24823604, 24824242, 24825857, 24829135, 24829623, 24830862, 24832651, 24837404, 24843554, 24845362, 24846663, 24847527, 24850070, 24851676, 24853843, 24853927, 24860040, 24860253, 24860410, 24861385, 24861473, 24863053, 24864092, 24867291, 24867565, 24869775, 24872213, 24876331, 24876366, 24884852, 24884953, 2488615, 24886399, 24886683, 24890479, 24891305, 24891714, 24892850, 24894024, 24895881, 24897880, 24902727, 24904929, 24905377, 24905427, 24906429, 24907486, 24908051, 24909772, 24910163, 24912029, 2491425, 24918022, 24923093, 24926203, 24928316, 24930207, 24931925, 24932585, 24932657, 24933462, 24933499, 24935571, 24939282, 24941089, 24941823, 24942875, 24949332, 24949412, 24949810, 24953317, 24956238, 24956687, 24960474, 24962406, 24962627, 24966637, 24967026, 24968294, 24975297, 2497591, 24976066, 24979767, 24980588, 24984166, 24984654, 2499071, 24991737, 24991771, 24992040, 24992518, 24992674, 2499660, 24999153, 24999336, 24999594, 25000014, 2500125, 25004637, 25005078, 25007113, 25007649, 25009160, 25015371, 25016301, 25017066, 25020173, 25025905, 25028170, 25030029, 25031088, 25038193, 25038975, 25040342, 25043073, 25047174, 25048527, 25050758, 25051020, 2505441, 25060386, 25060545, 25061085, 25062458, 25063095, 2506766, 25069901, 25072362, 25073712, 25074308, 2507508, 25075330, 25075569, 25077998, 25078751, 25079789, 25080217, 25085770, 25089211, 25091989, 25092839, 25093992, 25096937, 25099053, 25100214, 25101231, 25104317, 25104648, 25104935, 25106863, 25107374, 25108195, 2510932, 25112997, 25113374, 2511617, 2512410, 25125958, 25129039, 25132414, 25141778, 25145120, 25146455, 25146907, 25149145, 25149779, 25153010, 25155236, 25164300, 25165569, 25168812, 25170485, 25171258, 25172188, 25175354, 25175709, 25176445, 25178905, 25179757, 25179997, 25180682, 25183056, 25190622, 25191040, 25196219, 25196251, 25198143, 2520163, 25203252, 25206424, 25208922, 25210535, 25210867, 25211545, 2521547, 25216828, 25217732, 2521801, 25219035, 25222526, 2522557, 25226385, 25246723, 2524794, 25253860, 25255110, 25256153, 25256286, 25261239, 25262969, 25263237, 25263561, 25263867, 25264499, 25264894, 25269842, 25277782, 25279649, 25279650, 25281389, 25285156, 25288334, 25288512, 25292090, 25292226, 25294127, 25295004, 25295062, 25295067, 2529819, 25305724, 25305791, 25311113, 25312299, 25313690, 25318174, 25318911, 25319951, 25320769, 25321504, 25321744, 25322737, 25324978, 25327707, 25328330, 25332007, 25332315, 25338294, 25339865,	

		25341498, 25342236, 25342355, 25343442, 25346100, 25346102, 25347163, 25348288, 25354177, 25362450, 2536416, 25366711, 2536675, 25368656, 25369299, 25370418, 25371528, 25372632, 25373151, 25377567, 25379131, 25382260, 25382987, 25384467, 25388257, 25389054, 25390503, 25393535, 25393897, 25395001, 25395621, 25395942, 25396143, 25399776, 25400039, 25400623, 25401016, 25401504, 25402166, 25402402, 25403510, 25403583, 25404694, 25405875, 25408886, 25408907, 25410257, 25412790, 25413399, 25414227, 25414820, 25415323, 25415371, 25415617, 25418895, 25419274, 25420661, 25420947, 25422451, 25429929, 25434225, 25434757, 25435660, 25437700, 25439207, 25440008, 25442473, 25442561, 25443080, 25444487, 25444521, 25445797, 25448479, 25448571, 25448981, 2545025, 25450906, 25451287, 2545271, 25461224, 25463054, 25463245, 25464059, 25466082, 25470262, 25473152, 25473179, 25475136, 25476203, 25479944, 25480364, 25482909, 25483907, 2548666, 25487438, 25488620, 25495188, 25496001, 25499074, 25499685, 25503286, 25504806, 25505719, 25505854, 2550656, 25507659, 25508186, 25511841, 25520343, 25520447, 25521058, 25526452, 25527394, 2552959, 25530708, 25534832, 25536958, 25540372, 25540407, 25548035, 25548384, 25551064, 25553582, 25555213, 25555840, 25558913, 25559170, 25560176, 25561339, 2556269, 25565137, 25570048, 25570793, 25571691, 25573229, 25575738, 25576683, 25576963, 2557837, 25579538, 25584094, 2558842, 25589049, 25596376, 25596420, 25596479, 25598348, 25598662, 25602205, 25602231, 25603564, 25604982, 25605585, 25607244, 25609247, 25611892, 25612260, 25616163, 25617656, 25617860, 25619461, 25620450, 25620897, 25621404, 25621604, 25623241, 25624878, 25625085, 25625363, 25625378, 2562724, 25628144, 25628320, 25630550, 25631349, 25631523, 25633333, 25640005, 25641873, 25644464, 25645726, 25647572, 25649234, 25651042, 2565217, 25656578, 25656639, 25657572, 25658909, 25661606, 25663900, 25664319, 25666774, 25669122, 25669959, 25671199, 25671835, 25672500, 25675710, 25677592, 25680431, 25680876, 25683521, 25685216, 25686258, 25688383, 25691333, 25691926, 25694903, 25695028, 25698793, 25701044, 25705242, 25708986, 25712548, 25713757, 25715592, 25716960, 25720016, 25721352, 25722548, 25725358, 25726287, 25727015, 25727316, 25728349, 25728785, 25729162, 25729368, 25730109, 25732274, 25733269, 2573761, 25737913, 2573918, 25747656, 25753318, 2575538, 25755569, 25755966, 25756214, 25756361, 25758961, 25759054, 25759595, 25760478, 25763758, 25765969, 25766196, 25767048, 25767673, 25768693, 25770993, 25782764, 25784290, 25788052, 25789306, 25789755, 25792194, 25792870, 25797704, 25799124, 25804487, 2580548, 25805701, 25806053, 25807438, 25809192, 25811725, 25812100, 25812572, 25812588, 25813976, 25815136, 25815395, 25823168, 25826886, 25827339, 25828369, 25829329, 25832079, 25832539, 25832914, 25835881, 25836962, 25838565, 25842257, 25842864, 25845389, 25846944, 25847911, 25847912, 25855084, 25856953, 25858173, 25860047, 25869655, 25872641, 25872712, 25875486, 25876182, 25878658, 25882886, 25884111, 25885193, 2588784, 25892206, 25897257, 25901491, 25904123, 25904427, 25905744, 25910023, 25911323, 25911503, 25912877, 25915449, 25916879, 25917172, 25923124, 25929130, 25934621, 25936714, 25940827, 25940840, 25949727, 25950860, 25952953, 25953456, 25956785, 25958386, 25959525, 25960367, 25960757, 25961523, 25961968, 25964261, 25965009, 25965821, 25967290, 25971877, 25972868, 25973637, 25973982, 25976610, 25979862, 25980172, 25980815, 2598100, 25982872, 25984018, 25989241, 25989905, 25990098, 25991265, 25993369, 25994016, 25994785, 26000037, 26002053, 2601365, 26015630, 26016874, 26017559, 26022234, 26025520, 26028118, 26030269, 26031072, 26031101, 26031226, 26032560, 26035185, 26037138, 26038549, 26039226, 26039551, 26042075, 26042185, 26043987, 26045358, 26049389, 26049478, 26050589, 26052364, 26055247, 26055668, 26056874, 26067833, 26071572, 26071632, 26072231, 26072357, 26073522, 26073548, 26073827, 26074135, 26081680, 26086513, 26088304, 26090203, 26090678, 2609087, 26091097, 26091297, 26092391, 26092804, 26094595, 26096891, 26097968, 26098610, 26099271, 26100133, 2610288, 26104518, 26104663, 26105484, 26105817, 26106085, 26108777, 26109775, 26116552, 26118813, 26119651, 26120828, 26122972, 26125057, 26126762, 26131853, 26133599, 26135612, 2613882, 26142061, 26143001, 26145254, 26149019, 26150027, 26155623, 26155826, 26158608, 26161143, 26161283, 26161337, 26162035, 2616261, 26164284, 26165156, 26167778, 2616801, 26168616, 26169102, 2617231, 26174757, 26175404, 26176921, 2618049, 26181331, 26182166, 26182196, 26182372, 26186337, 26186625, 26187173, 26190954, 26195763, 26195815, 26197475, 26197614, 26200102, 26200752, 26201288, 26201397, 26202887, 26203017, 26206249, 26206287, 26207461, 26209244, 26211591, 26215453, 26215719, 26216194, 26217279, 26219655, 26220154, 26221525, 26221549, 26224019, 26227382, 26229336, 2623603, 26236071, 26237355, 26238251, 26241579, 26243722, 26244939, 26248822, 26249736, 26251974, 26253495, 26254643, 26256173, 26256458,	

		26256887, 26258031, 26259652, 26259670, 26261299, 26262711, 26265181, 26267246, 26267596, 26269987, 26270019, 26270265, 26272735, 26274263, 26278128, 2627816, 26281753, 26284361, 26284447, 26288444, 26289215, 26289316, 26290493, 2629418, 26294342, 26297428, 26298807, 2630097, 26307071, 2631082, 26311533, 26312916, 26313520, 26316406, 26319255, 26320633, 26320845, 26328348, 26330159, 26330939, 26335399, 26335438, 26335666, 2633606, 26336608, 26346907, 26347477, 26350230, 26350658, 2635358, 26353814, 26358190, 26359235, 26359245, 26361511, 2636462, 26367229, 26367680, 26370936, 2637908, 26380441, 26380829, 26382105, 26383186, 26384183, 26384501, 26387521, 26391791, 26394127, 26399125, 26400076, 26400569, 26403890, 26406946, 26408976, 26410139, 26412141, 26413194, 26415128, 26421843, 2642279, 26423799, 2642837, 26428723, 26430510, 2643395, 26434934, 26437099, 26437371, 26440532, 26446683, 26447218, 26449196, 26451156, 26452882, 2645391, 26454623, 26457419, 26460480, 26463227, 2646423, 26466397, 26469267, 26469780, 26470368, 2647041, 26471054, 26475458, 26475997, 26478115, 26479699, 26481288, 26482624, 26483255, 26484044, 26488048, 26494106, 26494651, 26495887, 26497318, 26497699, 26498624, 26501457, 26508452, 26509824, 26510830, 26511270, 26520294, 26520369, 26521314, 26522600, 26522708, 26524452, 26525595, 26525650, 26529550, 26529774, 26531016, 26536379, 26541033, 26542040, 2654533, 26550507, 26551218, 26553293, 26553490, 26554590, 26556492, 26559024, 26559564, 26560359, 26562935, 26562987, 26567020, 26568707, 26568885, 26568939, 26574831, 26574982, 26575348, 2657690, 26578486, 2658097, 26581329, 26586695, 26587681, 26589234, 26589506, 26590188, 26590207, 26593137, 26593489, 26594296, 26595254, 2659584, 26596759, 26597247, 26600800, 26603107, 26605110, 26605538, 26609250, 26617321, 26617930, 26622513, 26628730, 26631170, 26632886, 26633412, 26633698, 26635562, 26635679, 26636208, 26639175, 26645864, 26648755, 26651733, 2666148, 26665472, 26667607, 26669869, 26670355, 26675708, 26676070, 26678517, 26679770, 26679779, 26680759, 26682605, 26685022, 26685587, 26686486, 26687238, 26687624, 26688330, 26693851, 26695755, 26697075, 26700400, 26705542, 26706033, 26706910, 26709576, 26709923, 26710412, 26710822, 26712728, 26714699, 2671933, 26720295, 26721131, 26721445, 26724327, 26726306, 26729118, 26730125, 26731955, 26739543, 2674299, 26744676, 26750399, 26751024, 26751991, 2675314, 26756028, 26759778, 26759925, 26759956, 26761093, 26764383, 26774606, 26778486, 26779151, 26779321, 2678066, 26781159, 26782990, 26785042, 26785164, 2678591, 26791217, 2679226, 26794281, 26796119, 26798406, 26803161, 26803197, 26803680, 26805365, 26805687, 26807705, 2680871, 26811081, 26813256, 2681353, 26814408, 26815770, 26821754, 26823590, 26825866, 26827605, 26828827, 26831027, 26832992, 26834651, 26837962, 26843658, 26843995, 26847838, 26848646, 26851390, 26851409, 26851591, 26852910, 2685391, 26854353, 26854864, 26856920, 26857163, 26859658, 26866788, 26866837, 26869989, 26872920, 26873412, 26873910, 26876205, 26880641, 26886149, 26886483, 26888745, 2688877, 26892188, 26894667, 26894922, 2689677, 26898592, 26900729, 26900942, 26906175, 26911858, 2691200, 26914717, 26915665, 26916810, 26924933, 26924958, 26925878, 26926676, 26929106, 26931862, 26932754, 26933352, 2693388, 26937329, 26937627, 26939366, 26939644, 26939937, 26942078, 26942846, 26943387, 26945662, 26946624, 26946861, 26951423, 26963367, 26966856, 26967151, 26968807, 26970136, 26974812, 26974924, 26976834, 26978342, 26978983, 26981315, 26982473, 26984176, 2698430, 26984364, 26986520, 26987736, 26987901, 26988184, 26989003, 26990104, 2699215, 26995023, 27000889, 27003284, 27004647, 27005281, 27007236, 27007336, 27007681, 27009584, 27014575, 27021020, 27021671, 27024912, 27025900, 27027383, 27029099, 27030561, 27032883, 27037026, 27039260, 27040591, 27043438, 27043498, 27043706, 27047156, 27050906, 27053392, 27056713, 27060080, 27061983, 27062173, 27062681, 27067238, 27068460, 27070691, 27071299, 27074005, 27076165, 27076784, 27077797, 27080157, 27080263, 27082744, 27084649, 27085715, 27086543, 27089016, 27092657, 2709339, 27094015, 27094763, 27096798, 27097747, 27100128, 27100708, 27102424, 27105209, 27105903, 27107481, 27110226, 27111406, 27113406, 27113880, 27118867, 27119539, 27119828, 27122371, 2712317, 2712495, 27128199, 27134213, 27136560, 27137743, 27139672, 27145612, 27146668, 27147666, 27148143, 27153075, 27161390, 27161764, 27163491, 27164071, 27164334, 27164579, 27165035, 27165248, 27169411, 27173989, 27175547, 27179861, 27180298, 27184593, 27187359, 27191351, 27193591, 27198759, 27198789, 27199795, 27208009, 2721017, 27213574, 27215010, 27215221, 27225759, 27229500, 27235578, 27238206, 27242420, 27245133, 27245918, 27246174, 27247646, 27250189, 27251709, 27252657, 27257372, 27258929, 27264388, 27265356, 27271526, 27271808, 27275836, 27276207, 27285854, 27286381, 27286960, 27294565, 27294751, 27295911, 27297218, 27300186, 27302976, 27309085, 27310433, 27311391, 27313553, 27315785, 27316262, 27320849, 27323867, 27327294,	

		27334527, 27335720, 27339487, 27341175, 27341371, 27342458, 27344826, 2734801, 27350752, 27350907, 27353116, 27354199, 27358177, 27363713, 2736400, 27364875, 27365544, 2736560, 27365741, 27366410, 27368055, 27373870, 27375464, 27375612, 27380603, 27381384, 27381399, 27381838, 2738343, 27391222, 27391305, 27393176, 27394306, 27394385, 27397580, 27401623, 27404093, 2741189, 27414921, 27415317, 27416518, 27417783, 27419530, 27422101, 27424216, 27426104, 27427345, 27428376, 27435763, 27436082, 27440808, 27443987, 27446964, 27449018, 27449916, 27450257, 27450952, 27453560, 27454080, 2746613, 27471546, 27473522, 27473618, 27474567, 27475632, 2747590, 27480521, 27480623, 27481008, 27487500, 27489059, 27494418, 27494921, 27497377, 27502545, 27503283, 27504124, 27504556, 27505831, 2750621, 2750650, 27507219, 27513415, 27513524, 27514901, 27514926, 2751626, 27516339, 27517698, 27518044, 27518496, 27518807, 27520601, 27520818, 27520906, 2752104, 2752319, 27524670, 27525247, 27528266, 27528419, 27528681, 27535856, 2754297, 27543960, 27546930, 27548814, 27549571, 27550396, 27555051, 27556200, 27556367, 27559214, 27560723, 27561351, 27562582, 27563648, 27572254, 27572402, 27573921, 27576136, 27577102, 2757895, 27582540, 27586699, 27586946, 27592626, 27595856, 27597233, 27598509, 2760032, 27604878, 27605290, 27606669, 27607155, 2760902, 27610909, 27611077, 27614592, 27616633, 27617337, 27617657, 27617869, 27618458, 27619532, 27622329, 27622979, 27624099, 27625364, 27629977, 27631703, 27632655, 27633393, 27644568, 27645533, 27652910, 27656637, 27656661, 27657032, 27658192, 27659309, 27659897, 27660731, 27663053, 27664234, 27668215, 27670602, 27671804, 27676328, 27676442, 27676759, 27677252, 27680267, 27685019, 27687004, 27687177, 27688383, 27694438, 27695501, 27696487, 2769668, 27698149, 27698704, 27699085, 27699706, 27701564, 27705013, 27707276, 27711899, 27713212, 27714086, 27714744, 27715397, 27715760, 27718144, 27720608, 27721267, 27724072, 27724725, 27726308, 27732498, 27735009, 27735981, 27737482, 27740615, 27741740, 27743332, 27744055, 27745000, 27747710, 27750827, 27751319, 27752805, 27754707, 27757207, 27757386, 27761059, 27764458, 27765988, 27766598, 27768641, 27773659, 27775307, 27778792, 27782051, 27785278, 27786512, 27786961, 27787412, 27788522, 27789081, 27789228, 27790443, 2779167, 27793668, 27796444, 27798034, 2780065, 27800967, 27809170, 27810983, 27811102, 27812322, 27815605, 27816084, 27818452, 27818917, 27820734, 27824358, 27826871, 27826973, 27827506, 27828490, 27829252, 27829448, 27830928, 27831560, 27833082, 27834476, 27834730, 2783617, 27838251, 27839890, 27844241, 27848501, 27852328, 27855161, 27857486, 27857909, 27860643, 27861533, 27861936, 27862434, 27864158, 2786591, 27866350, 27867866, 27869519, 27873519, 27874337, 2787443, 27874703, 27876718, 27877341, 27878781, 27879649, 27879673, 27879694, 27885819, 27887464, 27888686, 27888966, 27889735, 27890757, 27892067, 27892576, 27894243, 27894335, 27896763, 27897253, 27897618, 27898782, 27900699, 27901126, 27902199, 27905373, 27907034, 27910619, 27914064, 27915255, 27918539, 27919522, 27921015, 27921543, 27926002, 27930405, 2793357, 27934403, 27936816, 27937509, 27944203, 27947473, 27949195, 27951085, 27954107, 27955816, 27955922, 280143, 2801503, 2807073, 2808843, 2810204, 2810462, 2816523, 2818265, 2818489, 2819670, 2823122, 2824374, 282542, 2826364, 2826948, 2827646, 2831880, 2835135, 2835231, 2836466, 2837859, 2839481, 2841364, 2843675, 2843766, 284578, 2847154, 2850420, 2852254, 2853497, 2854220, 2854733, 2858100, 2861986, 2872849, 2879262, 2879881, 2879959, 2882884, 2891605, 289811, 2901055, 2903724, 2905436, 2905507, 2906793, 2910053, 2910463, 2912945, 2915087, 2917564, 2925656, 2931524, 2933531, 2942560, 2946239, 2955899, 2966922, 2968879, 2971013, 2982928, 2982990, 2983136, 2984346, 2984828, 2987711, 2988815, 2989867, 2990671, 2993397, 2994274, 2996809, 2997529, 2997569, 300480, 3007141, 3007214, 3007473, 3008817, 301016, 3010976, 3012594, 3014533, 3015981, 3017038, 3017900, 3018068, 3028829, 3030295, 3030727, 3034903, 3035095, 3037704, 3037960, 3038961, 3039859, 3041980, 3048908, 3049107, 3052958, 3057286, 3058402, 3058427, 3060986, 3064715, 3066059, 3068658, 3071383, 3071873, 3077991, 3078776, 3079824, 3079934, 3081036, 3083494, 308567, 3086643, 3087349, 3091590, 3098716, 3098804, 3101507, 3102551, 3103637, 3106034, 3108804, 3110419, 3110780, 311159, 3111765, 3119755, 3121608, 3122362, 3122506, 3128108, 3128226, 3131334, 313453, 3134589, 3134852, 314397, 3145385, 3153596, 3155593, 3155790, 3158603, 316289, 3166491, 3169338, 3171549, 3183510, 3183932, 3183999, 3191530, 3194824, 3198834, 320444, 3214258, 3215182, 3220852, 3221218, 3228615, 3231658, 323214, 323366, 323449, 3241757, 32437, 3245855, 3253032, 3253247, 3255349, 3258107, 3258843, 3259471, 3260562, 3262335, 3262809, 3275, 3277450, 3278978, 3281711, 3295773, 329591, 3300758, 3306182, 3307599, 3309772, 3311334, 3319819, 3320274, 3324764, 3332484, 3343089, 3344556, 3345183, 3348191, 3348898, 3354586, 3359586, 3360812, 3362980, 3365058, 3375504, 3379237, 3384655, 3385411,	

		338580, 3388290, 3389807, 338991, 3389993, 3391833, 3394708, 3395474, 3395665, 3396477, 3399555, 340021, 3401059, 3401584, 3403536, 3403550, 3407453, 3411461, 3415197, 3416305, 3418672, 3418735, 3421943, 3423153, 3423897, 3425598, 343105, 3432101, 3433800, 3436509, 3454349, 3457089, 3458586, 3458915, 3468090, 3471218, 347454, 3475954, 3476860, 3477051, 3478008, 3478407, 3478963, 3484486, 3486560, 3489552, 3490511, 3491801, 3494376, 3494637, 3495514, 3495575, 3495586, 3496606, 3497027, 3498492, 3498818, 350048, 3501092, 3507226, 3508044, 3512753, 3517125, 3517639, 3517866, 3520264, 3521627, 3525172, 3526962, 3535531, 3536748, 3536779, 3537705, 3542852, 3545224, 3546266, 3548453, 355045, 3558940, 3561883, 3564881, 3570786, 3575454, 3585282, 3588004, 3588502, 3588687, 3589242, 3594777, 3599556, 3600279, 3600292, 3605397, 3609315, 3610878, 361316, 3616439, 3617306, 3618637, 3620048, 3626642, 3629175, 3630836, 3632627, 364035, 3642157, 3653720, 3655147, 3658544, 3664493, 3667616, 3670395, 3674372, 3675109, 3676570, 3678825, 3679899, 3684194, 3684570, 3685452, 3689778, 3692316, 3692569, 3695442, 3696781, 3699939, 3702858, 3703694, 3703745, 371170, 3714614, 3717379, 3725257, 3726443, 3730490, 3731525, 3736139, 3737706, 3738930, 3742237, 3742828, 3743340, 3752472, 375379, 3754026, 3759918, 3763263, 3773526, 3774236, 3774726, 3780862, 3790557, 3792529, 3793618, 3794168, 3795381, 3800183, 3805745, 3807137, 3810092, 3815186, 3815481, 3822737, 3827815, 3828150, 3828309, 3831161, 3836311, 3839867, 3839995, 3840553, 3840789, 3842879, 3860820, 3861003, 3861142, 3863788, 3883214, 3888829, 3889913, 3895975, 3898078, 3905441, 3908323, 3910436, 3911402, 3917116, 3917748, 3919134, 391994, 3928325, 3930415, 3931682, 3933523, 3933795, 3937538, 3938828, 3940829, 394375, 3945546, 3950565, 3950748, 3951265, 3957735, 3957926, 3958408, 3958809, 3959088, 3960322, 3960415, 3962920, 3965157, 3966140, 3966664, 3968817, 3969301, 3975479, 3980085, 3981085, 3981565, 3985317, 3986354, 3989196, 3993436, 3993693, 3995524, 3996334, 3998987, 4000645, 4004603, 4006751, 4012524, 4013130, 4023621, 4025750, 4026900, 4027973, 4032043, 4032470, 403440, 4036955, 4039158, 4041298, 4044528, 4053277, 4055868, 4059038, 4059567, 4060015, 4064576, 4067308, 4067703, 4079342, 4081404, 4086500, 4091491, 411765, 415693, 4193936, 421493, 425274, 4306135, 4310509, 4314120, 4336940, 4352917, 435350, 435554, 437895, 4379287, 4384487, 447041, 449143, 4501841, 452216, 4531025, 456339, 4565545, 459220, 459528, 4621369, 463115, 466912, 4673888, 4685942, 468746, 469862, 4724074, 474598, 475176, 4752034, 478474, 478640, 4808707, 4812395, 482454, 488626, 489138, 489205, 490852, 4928006, 493093, 496608, 497201, 4978742, 5013684, 50210, 5031207, 5033839, 505929, 508292, 5090868, 509788, 511212, 5130611, 513978, 51589, 521143, 528036, 532714, 535141, 5498617, 550624, 552245, 552277, 554248, 5559145, 557316, 566406, 568357, 5688933, 573366, 58044, 581022, 581733, 5971035, 599575, 6084812, 6086323, 6086953, 6087288, 60896, 6089646, 6090827, 6099172, 6099178, 6099982, 6106740, 611485, 6115679, 6116550, 6121444, 6124215, 6125541, 6126333, 6128154, 6129559, 6133787, 6141756, 6149843, 6151953, 6153998, 6159851, 6160926, 6161912, 6162383, 6164247, 6164489, 6177266, 6177831, 6179098, 6181818, 618333, 6184716, 6186006, 6187706, 6189661, 6192279, 6194613, 6194740, 6195487, 6200905, 6203213, 6205247, 6208884, 6208977, 6209222, 6215854, 6217918, 6218331, 6222429, 6226873, 6232351, 6234435, 6234685, 6235783, 6237020, 6242029, 6246813, 6248163, 6250748, 625128, 6251963, 6253529, 6254558, 6256395, 6257327, 6259179, 6263317, 6264812, 6269178, 6269960, 6270738, 6274913, 6278943, 6284866, 6294625, 6300291, 6306251, 630689, 6307954, 6308243, 6309805, 6310770, 6312850, 6315119, 6315199, 6317112, 6319076, 6321881, 6322863, 6326038, 6332622, 6334505, 6336931, 6339892, 6339994, 6340641, 6340670, 6348208, 6348779, 6350156, 6365563, 6371479, 637200, 6372971, 6374165, 6376266, 6378614, 6380187, 6384427, 6389611, 638991, 639013, 6390762, 6391562, 6391599, 6393296, 6394871, 6401440, 6403443, 6406021, 6408961, 6410304, 6412556, 6414365, 6415242, 6416400, 6418388, 642101, 6424586, 6426981, 6428287, 6431278, 6434682, 6434915, 6437482, 6439230, 6440087, 6449388, 6452055, 6454164, 6454542, 6455072, 6456714, 6457299, 6458438, 6461806, 6464506, 6467235, 647436, 6474448, 6476871, 6478560, 6478638, 6479913, 6484940, 6486215, 6491356, 6492002, 6495267, 64958, 6496741, 6500447, 6503506, 6503510, 6507682, 6511791, 6514212, 6514495, 6518791, 6521202, 6525170, 6528705, 6530554, 6530910, 6531438, 653519, 6537053, 653746, 6537964, 6539798, 6541117, 6543576, 6546349, 6546473, 6546671, 6555436, 657106, 657396, 6575768, 657852, 6584936, 6587530, 6588525, 6601203, 6601306, 6606012, 6610211, 6618100, 661816, 6619772, 6622613, 6624424, 6625707, 6626074, 6628239, 6644449, 6645862, 6646651, 6648970, 6652169, 6663805, 6665737, 6666466, 6667922, 6669405, 6673834, 6679512, 6680261, 6683588, 6685438, 6687558, 6690400, 669211, 6695592, 6695846, 6696917, 6701202, 6702429, 6702465, 6702698, 6704394, 6705404, 6706730, 6707136, 6709496, 6714007, 6714331,	

6716162, 6721888, 6726865, 6727729, 6730003, 6734517, 6735135, 6739379, 6740962, 6747182, 6758965, 6761592, 6764332,
6765121, 6766799, 6768830, 6769546, 6772873, 6778067, 6781288, 678437, 6784965, 678517, 6791537, 6793048, 679543,
6799915, 6800630, 6808886, 6810388, 6820056, 6821490, 6821613, 6822744, 6823592, 6824118, 6825694, 6829012, 6829310,
6830837, 6831358, 6834257, 6836245, 6842005, 6843154, 6843205, 6844073, 684670, 6849274, 685225, 6852819, 685397,
6854693, 6856387, 6856399, 6857630, 6858068, 6858070, 6858664, 6863320, 6864828, 6865317, 686596, 6869640, 6870159,
6871752, 6871907, 6878077, 6878260, 6878625, 6880053, 6881141, 6885622, 6890502, 6893707, 6921243, 6928193, 6928456,
6931938, 6939213, 6944595, 6954938, 6961138, 696864, 6968735, 6974590, 6975385, 6981720, 6985524, 6994006, 6998531,
7005015, 7007606, 7009503, 7009745, 7011371, 7014325, 7017096, 7018380, 702447, 7025660, 7033482, 7034916, 703588,
7037939, 7040133, 7040592, 7044147, 7046472, 7050981, 7051873, 7055595, 7055815, 7056278, 7058369, 7065012, 7067042,
7068632, 7068725, 7072897, 708226, 7084884, 7091568, 710288, 7104249, 7107644, 7114797, 711587, 711872, 7118730,
7118995, 7119657, 711980, 7128818, 7135498, 7137104, 7143032, 7143251, 7143465, 7153201, 7153592, 7157545, 7166563,
7191866, 7196225, 719703, 7198327, 7201923, 7202610, 7203826, 7204217, 7206636, 7209788, 7217168, 7224500, 7231977,
7234152, 7235816, 7236475, 7240078, 7245934, 7252057, 7257237, 7263360, 7272342, 7272495, 7275558, 7276100, 7276187,
7276435, 7277560, 7280135, 728116, 7282078, 7284235, 7290579, 729841, 7302318, 7304501, 7304573, 7310677, 7311183,
7317373, 7328598, 733132, 7340685, 7341733, 7346676, 7350155, 7358018, 7358667, 736164, 7363489, 7367089, 736802,
7372951, 7374879, 7374911, 7394053, 7395725, 7395923, 7400012, 7408780, 7409753, 7418262, 7426531, 7430384, 7435682,
74395, 7440097, 7441542, 7444058, 7450718, 7451013, 7451691, 7456325, 7458557, 7458626, 7458756, 7466813, 7475415,
7476195, 7478508, 7480344, 7484623, 7486296, 7490433, 7490771, 7499729, 7500381, 7501699, 7502173, 7503373, 7508327,
7515827, 751731, 7517626, 7517705, 7520752, 7520971, 752233, 7525876, 7527669, 7528176, 7529025, 7529367, 7530288,
7530933, 753442, 7536770, 7539795, 7540830, 7542145, 7543876, 7546263, 7546328, 7548069, 7549293, 7549789, 7550053,
7550548, 7553130, 7553834, 7554710, 7556943, 7558312, 7558464, 7562336, 7567696, 7567884, 7568232, 7568445, 7569549,
7569576, 7574391, 7574777, 7578055, 7580850, 7580995, 758140, 7583901, 7584015, 7585152, 7587804, 7593003, 7595335,
7595700, 7597601, 7605533, 7620995, 7622998, 7625123, 7626243, 7630213, 7631511, 7636093, 7644105, 7645028, 7645238,
7645463, 7646358, 7646893, 7647327, 7651564, 7654091, 7656400, 7667038, 7667926, 7673266, 7673773, 7673870, 7677299,
7679012, 7687582, 7688642, 7689484, 7691626, 7693046, 7694468, 7695302, 7700013, 7700061, 77019, 7702575, 7709055,
7710156, 7713167, 7714679, 7715578, 7717243, 7725768, 7726075, 7726776, 7727509, 7728771, 7729709, 773161, 7732787,
7734390, 7740660, 7742729, 7750122, 7750426, 7751461, 7756158, 7757090, 7759066, 77601, 7761292, 7764627, 7766899,
7772037, 7773085, 7785653, 7785794, 77893, 7789896, 779003, 7791565, 7792368, 7800687, 7800871, 7801141, 7807262,
7812013, 7812557, 7813945, 7817727, 7818568, 7823029, 7823587, 7825566, 7831338, 7831911, 7833378, 7840098, 7842412,
7842585, 7842921, 7843429, 7843643, 7845393, 7851628, 7852025, 7853208, 7853495, 7857759, 7858216, 7858282, 7859048,
7867509, 7868682, 7868750, 7875993, 7878112, 7880958, 7890778, 7893106, 7894255, 7902996, 7908121, 7912431, 7917937,
7919615, 7920079, 7921548, 7921762, 7922169, 7922660, 7927397, 7933297, 7933609, 7934631, 7935701, 7937174, 7940640,
7941532, 7945251, 7945435, 7945914, 7949104, 7949278, 7949806, 7951575, 7953587, 7954216, 7956340, 7958882, 7962992,
7963529, 7964490, 7968078, 7968521, 7970143, 7971585, 7972893, 797381, 7976452, 7977853, 7980256, 7981633, 7984052,
7984839, 7986197, 7995563, 8005806, 8006000, 8006349, 8009333, 8010096, 8011182, 8013541, 8013959, 8015556, 8016700,
8017677, 8020227, 8022709, 8023190, 8024450, 8024647, 8026267, 8027518, 8032577, 8034605, 8042124, 8042874, 8043156,
8045379, 8050039, 8050534, 8050906, 8051844, 8053262, 8053585, 8055901, 8057308, 8057551, 8059893, 8063972, 8065463,
8065496, 8067469, 8067980, 8070457, 8072520, 8073117, 8075149, 8076645, 8076665, 8076826, 8079642, 808073, 8085687,
8087655, 809031, 8091200, 8091609, 8094939, 8095947, 8098906, 8104555, 8106122, 8112521, 8112588, 8117281, 8117693,
8119309, 8121772, 8124219, 8124944, 8125498, 8126006, 8131304, 8134991, 8137247, 8141922, 8144756, 8147151, 8148272,
8151413, 8160955, 8161507, 8161994, 8163701, 8166642, 8168351, 8171132, 8171148, 817192, 8173183, 8174553, 81755,
8176720, 8179847, 818318, 8186981, 8191830, 8192609, 819425, 8198563, 8199694, 8202170, 820228, 8203924, 8208339,
820944, 8209573, 8211013, 8212222, 8215760, 8217488, 8218105, 8219019, 8220460, 8221914, 8224882, 8228314, 8229417,
8229515, 8229638, 8229710, 8230904, 8231334, 8231661, 8232280, 8237095, 8237325, 8238378, 8240393, 8242232, 8243167,

8243927, 8245265, 8246976, 8249345, 8251524, 8252064, 8253852, 8254071, 8257786, 8262702, 8264991, 826532, 8272692,
8273728, 8275976, 8277431, 8279444, 8285060, 8286223, 8287769, 8288453, 8289015, 8290500, 8290835, 8292920, 8294047,
82949, 8295093, 8295479, 8298808, 8299159, 8299296, 8302020, 8302514, 8307246, 8308368, 8310087, 8316357, 8317060,
8318355, 8319506, 8322325, 8324775, 8324906, 8336336, 8339565, 8342625, 8344055, 8345249, 8345572, 8346592, 8348225,
8349868, 8352381, 8352802, 8359153, 8361309, 836491, 8365048, 8369453, 8372764, 8374504, 8377161, 8377257, 8379752,
8379926, 8388304, 8390477, 8390706, 8390896, 8391411, 8391423, 8392776, 839299, 8397851, 8398647, 8401309, 8401504,
8402630, 8411393, 8412051, 8412691, 8414752, 8414779, 8417992, 8422385, 8424483, 8430334, 8433090, 8434992, 8436214,
8440140, 844050, 8440671, 8445790, 8448811, 8449182, 845232, 8453820, 8456899, 8457416, 8458922, 8458936, 8473558,
8476254, 8476387, 8486627, 8488392, 8491194, 849513, 8500081, 8500633, 8501430, 8503864, 850995, 8510297, 8511241,
8525779, 8526424, 8527222, 8529562, 8531212, 8531408, 8539295, 8539499, 8548984, 8549080, 8551038, 855132, 8553460,
8556593, 8556891, 8557090, 8558275, 8559193, 8559629, 8562708, 857110, 8573904, 8574663, 8574704, 8575257, 8575930,
8578444, 8582123, 8582385, 8587531, 8587565, 8588485, 858947, 8593487, 8597830, 8601034, 8602554, 8603873, 8604979,
8606845, 8609995, 8611635, 861168, 8619858, 8622800, 8623975, 8625547, 8626841, 8627067, 8633586, 8634435, 8637691,
8640655, 8640743, 8641960, 8643691, 8645049, 8651537, 8651773, 865264, 8652737, 8653422, 8654195, 8654388, 8654580,
8656983, 8657457, 8663335, 8667014, 8667410, 8670202, 8675547, 8675874, 8679824, 8680555, 8681978, 8683358, 8683661,
8684660, 8684836, 8694511, 8696340, 8699127, 8700965, 8702736, 8704062, 8706522, 8706823, 8707054, 8711174, 8712523,
8714464, 8721685, 8722028, 8727589, 8730223, 8731713, 8734706, 8735398, 8735521, 8738613, 8739333, 874544, 87503,
8756232, 8757077, 8760320, 876087, 8761102, 8762449, 8763410, 8764491, 8764799, 8767150, 8769645, 8772521, 8774918,
8779812, 8785280, 8791049, 8792618, 879606, 8796367, 8799218, 8800862, 8805935, 8807927, 8809808, 8810809, 8813539,
8814412, 8815893, 8817365, 8821305, 8824062, 8824331, 8824666, 8826690, 8831303, 8831680, 8831955, 8835181, 8835664,
8836929, 8838572, 8839926, 884415, 8844494, 8844890, 8845330, 8846178, 8846879, 8847631, 8854336, 8871404, 8872923,
8873514, 8876451, 8877452, 8878020, 8878711, 8880477, 8883515, 8883924, 8884852, 8889844, 8891385, 8892026, 8892500,
8895758, 8900822, 8901221, 890208, 8906521, 8907631, 8912164, 8917348, 8923090, 8936305, 8943186, 8944821, 8944834,
8946663, 8946984, 8947049, 8948839, 8953384, 8953762, 8961046, 8961122, 8965738, 8966779, 8968192, 8977847, 8980557,
8982472, 8982620, 898295, 8983548, 8988945, 8989485, 8989829, 8990084, 8991538, 9001064, 9003018, 9003533, 9003875,
9009069, 9009870, 9010783, 9011091, 9013005, 9013429, 9017398, 9019238, 9024102, 9030811, 9031310, 9035671, 9036897,
9038176, 9038761, 9040940, 9042593, 9042948, 9043872, 9051684, 9052952, 9055281, 9056765, 9063977, 9064174, 9066176,
9067724, 9068839, 9069172, 9070572, 9074122, 9078370, 9078520, 9082588, 9086559, 9087169, 9088347, 9089530, 9090639,
9095076, 9098984, 9099073, 9100411, 9102843, 9103103, 9103520, 9103847, 9103976, 9104500, 9111806, 9112338, 9113584,
9118989, 9122060, 9125847, 9130312, 9130694, 9132121, 9133357, 9137835, 9139325, 9140080, 9140167, 9143026, 9145036,
9146729, 9146778, 9148239, 9149131, 9150036, 9152103, 9153593, 915459, 9154719, 9157330, 9158024, 9159376, 9161688,
9161915, 9163769, 9164940, 9165471, 9169139, 9169372, 9170428, 9170449, 9171413, 9171527, 9174928, 9175696, 9175962,
9177144, 9180634, 9181677, 9186806, 9187333, 9187933, 9192638, 9193029, 9193428, 9195218, 9195735, 9197228, 9198452,
9201773, 9206025, 9206571, 9211806, 9212924, 9216992, 9217374, 9217672, 9228651, 92288, 9230902, 9231324, 9240422,
9242091, 9243437, 9246375, 9249012, 9252325, 9255150, 9256640, 9259163, 9260487, 9260932, 9262027, 9262264, 9263187,
9264380, 9270342, 9270998, 9272011, 9272698, 9273776, 9276639, 9278429, 92864, 9293848, 9293958, 9294474, 9295763,
9303544, 9304220, 9307115, 9308194, 9308899, 9309416, 9309839, 9310004, 9314855, 932145, 9323079, 9323134, 9326166,
9326602, 9329024, 9331977, 9332575, 9333892, 9334104, 9338869, 9340736, 9344325, 9345254, 9345693, 9345914, 9347356,
9349914, 9350034, 935122, 9355080, 9356091, 935636, 9357964, 9358461, 9363533, 9364556, 9365814, 9371838, 9372056,
9372502, 9372503, 9372945, 9373705, 9374174, 9376168, 9376740, 9377191, 9379278, 9380437, 9384475, 9385652, 9386288,
9390230, 9393776, 9394547, 9394839, 9399433, 9400624, 9402214, 9403034, 9404409, 9405786, 9407030, 9408412, 94096,
941020, 9412461, 9415602, 9415857, 9417168, 9417457, 941792, 9421461, 9422615, 9425755, 9426691, 9430226, 9436111,
9438276, 9438525, 9438799, 9439592, 9439892, 944062, 9445034, 9445836, 9447488, 9450264, 9451757, 9460641, 9461098,
9464154, 9465362, 9474139, 947677, 9478327, 9481763, 9484267, 9487894, 9493463, 9494024, 9501215, 9506205, 9508328,

		9513331, 9514757, 9516943, 9520080, 9523212, 9523453, 9525532, 9527685, 9529320, 9530829, 9531938, 9534531, 9538623, 9539366, 9541434, 9547743, 9550287, 9552190, 9555256, 9556193, 9557957, 9558024, 9558554, 9562267, 9562321, 95627, 9563698, 9564934, 9567269, 9568659, 9570684, 9574099, 9575674, 9577752, 9584990, 9585550, 9585725, 9586722, 9588587, 9588719, 9588786, 9590369, 9591300, 9591686, 9596443, 9598468, 9608692, 9610423, 9614001, 9615816, 961739, 9617633, 9618504, 9618676, 9623714, 9626123, 9626185, 9626296, 9630152, 9630650, 9632256, 9633683, 9634497, 9643513, 9644085, 9648551, 9649016, 9650050, 9651081, 9651471, 9657993, 9661025, 9663725, 9665174, 9666332, 9666984, 9677151, 9680017, 9680667, 9686238, 9688467, 9691155, 969379, 9695917, 9697311, 9701524, 9707972, 97082, 9714886, 971617, 9716583, 9725466, 9726944, 97287, 9730251, 9733092, 9737750, 9740432, 974156, 9742590, 9747204, 9750345, 9751959, 9755876, 9757133, 9757738, 9758723, 9759542, 9759627, 97608, 97613, 9762694, 9765139, 9766066, 9766087, 9767093, 9768452, 9769286, 9770221, 9770373, 9775677, 9776143, 9777017, 9778439, 9778646, 9778808, 9781306, 9783306, 9787317, 9791126, 9794840, 9797, 9797344, 9798939, 9802094, 9802359, 9804274, 9805135, 9809091, 9810581, 9813734, 9817938, 9821106, 9821489, 9826409, 9829875, 9831051, 9831734, 983469, 9836198, 9839186, 9839500, 984168, 9842425, 9844257, 9844397, 9847266, 9852962, 9854469, 9857965, 9866780, 986855, 9872143, 9874001, 9874080, 9874105, 987721, 9880214, 9880699, 9880922, 9884039, 9884241, 9887509, 9888352, 9888596, 9889085, 9889118, 9892786, 98943, 9895119, 989728, 9914285, 9914331, 9914420, 9916141, 9916361, 9919109, 9923990, 9925063, 9928418, 9930766, 9931169, 99326, 9933197, 9949743, 9950935, 9951971, 996448, 9972719, 997418, 9988599, 9990648, 999229,	
**False Negative**	None	11448407, 11988613, 17173200, 17297204, 20471772, 21310400, 24023667, 24704659, 26225583,	27792997,
**False Positive**	None	19120729, 20651016, 21697852, 22831982, 23488941, 24072556, 25527152, 25597924, 26587795,	None

Powered by Dancer 1.3202
