# Supplementary material for: The intriguing evolution of effect sizes in biomedical research over time: smaller but more often statistically significant
Source: Gigascience. 2017 Dec 6;7(1):gix121. doi: 10.1093/gigascience/gix121 (PMC5765564; doi:10.1093/gigascience/gix121)
Supplement: Supplemental materials [file gix121_supp.zip › Supplementary_data.pdf]

Supplementary Information for

The intriguing evolution of effect sizes in biomedical research over time: smaller but more often statistically significant

Paul Monsarrat, Jean-Noel Vergnes.

correspondence to: jn.vergnes@mcgill.ca

Supplementary Methods **3**
Methods for data mining 3
Details on the Perl algorithm for data mining of ESs 4
Performance tests of the algorithm 7
Definitions of citation characteristics 7
Supplementary Tables **9**
Additional Table 1 - Check for polysemy of terms related to types of ESs 9
Additional Table 2 - Examples of “undetectable” ESs, false negative ESs and false positive ESs 12
Additional Table 3 - Mathematical transformations and main outcomes 14
Additional Table 3a - Mathematical transformations 14
Additional Table 3b - Main outcomes 14
Additional Table 4 - Summary table of the analysis plan 15
Additional Table 5 - Geographical analysis 19
Supplementary Figures **22**
Additional Fig. 1 - Overview of the “Knowledge Discovery in Databases” (KDD) approach used in this study 22
Additional Fig. 1a – The different steps that compose the KDD process (adapted from Fayyad [40]) 22
Additional Fig. 1b – Flowchart of the algorithm for PubMed data mining changer 23
Additional Fig. 1c – Flow diagram of the selection process for abstracts (PubMed data mining) 24
Capital letters at the left of rectangles correspond to specific parts of the analysis plan detailed in 24
Additional Fig. 2 - Descriptive analysis of the comprehensive database and descriptive analysis of ESs in abstracts 25
Additional Fig. 2a - Proportion of abstracts containing at least one ES also increased over time 25
Additional Fig. 2b - A majority of OR and a trend for RR to be substituted by HR 25
Additional Fig. 2c - A majority of abstracts with ESs >1 25
Additional Fig. 2d - Disparities between geographical areas: the weight of Europe and North America, the growth of Asia 26
Additional Fig. 2e - The proportion of abstracts with a multivariate analysis increased with time 26
Additional Fig. 2f – The proportion of abstracts with a free full-text available increased with time 26
Additional Fig. 2g - The proportion of abstracts from Core Clinical Journals decreased with time 26
Additional Fig. 2h - Increase of the proportion of abstracts with at least one detected ES from Reviews 27
Additional Fig. 2i - 95%CI type represented the overwhelming majority of CIs, with no major evolution over time .. 27
Additional Fig. 3 - Histogram distribution of ESs (T#2) 27
Additional Fig. 4 - Heatmap of the temporal evolution of proportion of statistically significant ESs per abstract: disparities among fields of research 28
Additional Fig. 5 - ESs decreased over time 29
Additional Fig. 5a - Both “protective” and “risk” ESs decreased 29

Additional Fig. 5b - ES decreased for both protective and risk values 29
Additional Fig. 5c - HR, OR and RR ESs were decreasing 29
Additional Fig. 5d - The simultaneous increase of statistically significant tiny effects and decrease of statistically significant large effects 30
Additional Fig. 5e - ESs decrease in all continents 30
Additional Fig. 6 - Knowledge checking 31
Additional Fig. 6a - ESs from Reviews declined slightly but significantly with time 31
Additional Fig. 6b - ESs decrease was only found for ESs with 95% confidence intervals 31
Additional Fig. 6c - Knowledge checking: decrease of ESs was also observed among PMC full-texts 31
Additional Fig. 6d - The proportion of statistically significant ESs in Reviews remained stable over time 32
Additional Fig. 6e - No trend was found for 90% and 99% confidence intervals 32
Additional Fig. 6f - The proportion of statistically significant ESs increased over time for values in both Abstracts and Results of PMC full-texts 32
Additional Fig. 7 - Proportion of statistically significant ESs increased over time 33
Additional Fig. 7a - Proportion of statistically significant ESs increased for both “protective” and “risk” ESs 33
Additional Fig. 7b - Proportion of statistically significant ES increased for both “protective” and “risk” abstracts 33
Additional Fig. 7c - Proportion of statistically significant ESs increased whatever the type of ES (HR, OR and RR) 33
Additional Fig. 7d - Proportion of statistically significant ESs increased for nearly all the geographical areas 34
Supplementary File 1 35
Performance testing: kappa, sensitivity and specificity 35
Supplementary References 35

Supplementary Methods

Methods for data mining

The comprehensive database was generated using a Perl script specially developed for the present work. The flowchart of the proposed algorithm is presented in Additional Fig. 1b and the source code of the Perl script is available online [41]. The script was developed and run using Strawberry Perl 5.20.2.1-64bits on Windows 8.1 operating system (Professional edition), with 8 Go RAM memory. Three specific modules were developed:

1. ES_detector.pm is the main module for data preprocessing and data mining:

   1.1. Subroutine '/extract':

   - Concerned all PubMed abstracts.
   - The algorithm extracted effect sizes (ES) along with upper and lower limits of their confidence intervals, using the regular expression (or "regex") capacities of the Perl programming language.
   - Other characteristics: PubMed identifier (PMID), ±PMC identifier (PMCID), month/year of publication, detection of a multivariate analysis (yes/no), Open Access (OA) publication (yes/no), publication in a Core Clinical Journal (CCJ) (yes/no), CI level (i.e. 90%, 95% or 99%), and type of publication ("Review": yes/no).

   1.2. Subroutine '/extractPMC':

   - Concerned all PubMed abstracts with a detected ES and a PubMed Central full-text available in xml format.
   - ESs were extracted from the results sections and the tables of PMC articles.
   - For tables, ESs types were sought inside the table head, then ES values were identified from the corresponding columns. Confidence intervals were not retrieved because of the wide range of presentation in the tables.

   1.3. Subroutine '/random' and "/sensspe':

   - During the final iteration of the PubMed data mining, a special procedure was performed to select a random sample of enough abstracts to test the performance (sensitivity/specificity) of the data mining process. Based on our preliminary results (2% of total citations with abstracts reported at least one ES), we evaluated the number of abstracts that would be needed to accurately measure the specificity and sensitivity of the data mining process. Considering that approximately 200 abstracts with at least one ES should be necessary to assess sensitivity, we randomly selected 1 citation out of 1700 (over 16 820 871) to obtain a random sample of 9697 abstracts.
   - Once the sample had been collected, a specific program allowed User1 (author 1) and User2 (author 2) to independently assess each of the randomly selected abstracts, and identify putative False Negative or False Positive values. A kappa test was also performed as a measure of agreement between the two observers.

   1.4. Subroutine '/check_fft':

Using the list of included PMIDs, the PubMed database was queried to determine which citations were indexed as “free full-text” articles.

2. Load_module.pm:

- Using the database of the tool MapAffil, the different countries inside authors’ affiliations were identified. MapAffil provides access to disambiguated geo-political entities and institution types inferred from affiliation strings per paper in PubMed [46]. The last available update was 09/21/2016. MapAffil data were bulk downloaded with the consent of the owner, and the geographical locations were retrieved for each PMID. For PMIDs unknown by MapAffil (10 502 (4.2%) abstracts), countries were retrieved from affiliation strings by text mining using country names (in full or using the 3-letter code) and states/provinces (for USA and Canada), using the current norm ISO-3166.

- Open Access (OA) Journals indexed in the Directory of Open Access Journals (11/04/2016) were identified using ISSN numbers.

- The Core Clinical Journals list was exported from the NLM catalog (11/04/2016), and compared to the ISSN from the abstracts with a detected ES.

- This module also checked for polysemy of acronyms. Using the MediLexicon online database of pharmaceutical and medical abbreviations (http://www.medilexicon.com/), all potential synonyms were identified by text mining on entire abstract with some minor variations tolerated (plural, hyphen, spaces). For example, OR may be an acronym for “Ovulation Rate”, ORA for “Opioid Receptor Agonist” or ORS for “Oculo-Respiratory Syndrome”. See full details in Additional Table 1.

3. Mesh_detector.pm:

- This module was used to perform data mining on Mesh keywords. Mesh Headings were first detected using the 2017 Mesh database (MH code from the d2017.bin file). Variants of entry terms were also identified (ENTRY code from the d2017.bin file). To improve detection, Mesh Headings and variants were also identified after pre-treatment of the chains of characters (all in lowercase, parentheses and punctuation replaced by space).
- Several abstracts only contained free keywords provided by authors not related with Mesh keywords. To detect the maximum of these, the UMLS Metathesaurus was downloaded (file MRCONSO.RRF). This file contained a list of lexical variants [47]. If a free keyword was identified from the Metathesaurus, the corresponding Mesh word was retrieved (Mesh Headings related to the same medical concept, i.e. with the same CUI code).
- This package also listed the different MeSH keywords derived from [C] or [G], the “Diseases” and “Phenomena and Processes” branches, respectively.

Details on the Perl algorithm for data mining of ESs

Below are some lines of code, emphasized to explain the key elements of the data mining algorithm. The complete source code can be found in ES_detector.pm [41].

```
while($abstract =~
m/((?:(?:(?<!un)|(?<!not)|(?<!non)|(?<!no))\W?[aA]djust)?.{0,50}?\W[aA]?)((?:[Rr]elatives?\W*[
Rr]ates?)|(?:[Rr]elatives?\W*[Rr]isks?)|(?:(?:[Rr]elatives?\W*[Rr]isks?)|(?:[Hh]a[sz]ards?\W*[
Rrates?])|(?:[Pp]revalences?\W*[Rrates?])|(?:[Pp]revalences?\s[Oo]dds?)|(?:[Rr]isks?)|(?:[Oo]d
ds?)|(?:[Hh]a[sz]ards?)|(?:[Pp]revalences?)|(?:(?:[Ii]ncidences?)?[Rr]ates?))\W*[Rr]atios?|(?:
POR|por|Por|OR|IRR|irr|Irr|rrr|RRR|Rrr|hrr|HRR|HR|PRR|Prr|prr|PR|rr|RR|Rr))([sAa]?(?:djusted|c
rude)?\W)(.*?((?<!\d,)\d{1,3}(?:\.\d+)?|(?:\.\d+)).*?(?=\W[aA]?(?2).*)|.*(?!(?2)))/g){
        […]
        my
        ($c1,$c2,$c3,$abstractpart)=(defined($1)?$1:"",defined($2)?$2:"",defined($3)?$3:"",defi
        ned($4)?$4:"");
        […]
        while($abstractpart =~ m/(.*?)(?<![-
        \w\.])((?<!\d,)\d{1,3}(?:\.\d+)?|(?:\.\d+))([^\.\/\)\}\]\w]\D*?(?<![-
        \.\w<>]))((?2))([^=\+<>\.\/\)\}\]\w][^\d=\+<>]*?(?<![=\+<>\(\{\[\w]))((?2))(?![0-
        9]|\.[0-9])(?(?{($2<=$4||$2>=$6)})1)/g){
                […]
                %atrisk=Loadmod::identify_abs_atrisk($duplicate_abstract);
                […]
                if($duplicate_abstract =~
                m/(?:[Cc]onfidence\s+(?:[Ii]ntervals?|[Ll]imits?\s?|[Ll]evels?)|CI|c\.?i\.?)\D{1,
                10}(9[059])\W?(?:%|per\W?cent)?|[^\.0-
                9](9[059])\W?(?:%|per\W?cent)?\D{1,10}(?:[Cc]onfidence\s+(?:[Ii]nterval|[Ll]imits
                ?\s?|[Ll]evels?)|CI|c\.?i\.?)/){[…]}
                […]
                if((length($c5)<=100) && (length($c7)<=10)){
                        if(length($c2)<=4){
                                if(defined($atrisk{$ortemp2})){$atriskyn=$atrisk{$ortemp2}};
                        }
                        if((int($c4) eq "$c4") && (int($c6) eq "$c6") && (int($c8) eq
                        "$c8")){$atriskyn="WARNING"};
                        if((($c7 =~ m/,/)||(length($cx)>200)) && (int($c4) eq "$c4") && (int($c6)
                        eq "$c6") && (int($c8) eq "$c8")){$atriskyn="DANGER"};
                        […]
                }
                […]
        }
}
```

Red part of the algorithm

```
while($abstract =~ m/[…]/g){[…]}
```

The *regex* algorithm (=~) identifies (m/) all (/g) the following regular expressions that match the formula, one after the other. The algorithm is case sensitive.

```
((?:(?:(?<!un)|(?<!not)|(?<!non)|(?<!no))\W?[aA]djust)?.{0,50}?\W[aA]?)
```

This part allows the algorithm to record the characters placed before a detected ES in the text of the abstract. A maximum of 50 characters are extracted. A word beginning by “adjust”, or the letter “a” placed just before the ES, was identified from this string as an adjusted ES.

```
((?:[Rr]elatives?\W*[Rr]ates?)|(?:[Rr]elatives?\W*[Rr]isks?)|(?:(?:[Rr]elatives?\W*[Rr]isks?)|
(?:[Hh]a[sz]ards?\W*[Rrates?])|(?:[Pp]revalences?\W*[Rrates?])|(?:[Pp]revalences?\s[Oo]dds?)|(
?:[Rr]isks?)|(?:[Oo]dds?)|(?:[Hh]a[sz]ards?)|(?:[Pp]revalences?)|(?:(?:[Ii]ncidences?)?[Rr]ate
s?))\W*[Rr]atios?
```

```
|(?:POR|por|Por|OR|IRR|irr|Irr|rrr|RRR|Rrr|hrr|HRR|HR|PRR|Prr|prr|PR|rr|RR|Rr)
```

Several types of acronyms for ESs were detected: prevalence odds ratio (POR), odds ratio (OR), incidence rate ratio (IRR), relative risk ratio (RRR), hazard rate ratio (HRR), hazard ratio (HR), prevalence rate ratio (PRR), prevalence ratio (PR), relative risk (RR). The algorithm also considered some minor typographical differences, such as abnormal spaces between words, hyphen, singular or plural, upper or lower case of the first letter of the words. As the algorithm is case sensitive, the acronyms “or” and “Or” were ignored to minimize the risk of False Positive detection, thus avoiding confusion with the coordinating conjunction.

```
([sAa]?(?:djusted|crude)?\W)
```

A word beginning by "adjust", or the letter "a" placed just after the ES, was identified. The word "crude" was also tolerated. A non-alphanumeric \W is mandatory at the end.

```
(.*?(?=\W[aA]?(?2).*)|.*(?!(?2)))
```

After the type of ES was identified, the remaining characters were considered to detect the value along with its confidence interval. An important strength of this algorithm relied on the part `(?=\W[aA]?(?2).*)|.*(?!(?2))`. Two different cases were managed:

- First, if another type of ES (second pattern `(?2)`) was detected, adjusted or not `(?=\W[aA]?(?2).*)`, the considered characters were searched between the two ESs, and the characters after the second ES were ignored at this time `(?=[…].*)`.
- Second, if no other type of ES was detected, the considered characters were found after the ES, using the greedy quantifier `(.*)`.

This string (referenced by `$4`) was then stored in the variable `$abstractpart` for subsequent text mining.

```
($c1,$c2,$c3,$abstractpart)=(defined($1)?$1:"",defined($2)?$2:"",defined($3)?$3:"",defined($4)
?$4:"");
```

Ternary syntax. The `$c4` part contains all the ES values to be extracted after an ES type has been detected (further detected by the green part of the algorithm).

Green part of the algorithm

```
while($abstractpart=~m/[…]/g){[…]}
```

The *regex* algorithm (`=~`) identifies (m/) all (`/g`) the following regular expressions that match the formula, one after the other. The algorithm is case sensitive.

```
(.*?)
```

Contains all characters placed before the ES value. Adjusted values are also detected from this string.

```
(?<![-\w\.])
```

The ES value could not be preceded by an alphanumeric character, a point or a negative sign.

```
\d{1,3}(?:\.\d+)?|(?:\.\d+))
```

Between 0 and 3 numbers, followed or not by a point, followed or not by one number. An integer or decimal could thus be detected, but they must not be preceded by a negative sign. For some decimal numbers, the first zero could be absent.

```
([^\.\/\)\}\]\w]\D*?
```

The ES value should be followed by a non-alpha-numeric character, except some special characters: `. / ) }` This decreased the risk of False Positives, for example the wrong detection of a chemical formula (e.g. NH2-(CH2)3-SiO(4)).

```
(?<![-\.\w<>])((?2))
```

Another value `((?2))` must be detected for the left part of the confidence interval. The value must not be preceded by an alphanumeric character, a point or a negative sign.

```
([^=\+<>\.\/\)\}\]\w][^\d=\+<>]*?(?<![=\+<>\(\{\[\w]))((?2))
```

Another value `((?2))` must be detected for the right part of confidence interval. The value must not be preceded by some special characters, particularly closing characters.

```
(?![0-9]|\.[0-9])(?(?{($2<=$4||$2>=$6)})1)
```

Some checks are then performed: the extracted ES value must be situated between the extracted values of lower and upper confidence intervals. If not, the algorithm backtracks to find better values.

Black part of the algorithm

```
%atrisk=Mesh::identify_abs_atrisk($duplicate_abstract);
```

Given that an ES value has been detected, a check for polysemy of acronyms was performed for the entire abstract.

```
if($duplicate_abstract =~
m/(?:[Cc]onfidence\s+(?:[Ii]ntervals?|[Ll]imits?\s?|[Ll]evels?)|CI|c\.?i\.?)\D{1,10}(9[059])\W
?(?:%|per\W?cent)?|[^\.0-
9](9[059])\W?(?:%|per\W?cent)?\D{1,10}(?:[Cc]onfidence\s+(?:[Ii]nterval|[Ll]imits?\s?|[Ll]evel
s?)|CI|c\.?i\.?)/){[…]}
```

The confidence interval level was identified from the entire abstract, according to the definition given in the paragraph "Definitions of citations' characteristics" of Supplementary Methods.

```
if((length($c5)<=100) && (length($c7)<=10)){[…]}
```

A last check was performed about the length of the strings to decrease the risk of False Positive detection. The number of characters between the extracted ES value and the lower bound of the confidence interval, and between the upper and lower bound of the confidence interval must be less than or equal to 70 and 10 characters, respectively.

```
if(length($c2)<=4){if(defined($atrisk{$ortemp2})){$atriskyn=$atrisk{$ortemp2}};}
```

If the detected ES was an acronym `(?:POR|por|Por|OR|IRR|irr|Irr|rrr|RRR|Rrr|hrr|HRR|HR|PRR|Prr|prr|PR|rr|RR|Rr)`, the risk was assessed according to the control of polysemy previously achieved.

```
if((int($c4) eq "$c4")&& (int($c6) eq "$c6") && (int($c8) eq "$c8")){$atriskyn="WARNING"};
if((($c7 =~ m/,/)||(length($cx)>200))&& (int($c4) eq "$c4")&& (int($c6) eq "$c6") && (int($c8)
eq "$c8")){$atriskyn="DANGER"};
```

If the ES value and confidence interval bounds were integers, the risk was high that these values were false positive. So a confidence interval level was required to be included. But if the string length between ES type and its value was above 200 characters, or if characters between the lower and upper bounds contained a comma (risk of being a list of values, not ES), values were excluded ("DANGER").

Performance tests of the algorithm

The performance of the algorithm (designed to automatically detect ESs) was first tested at the ES level. The reference standard was the human recognition of 1) an OR/RR/HR type of ES (see Additional Table 1), 2) having a value that was strictly positive and 3) bounded by a confidence interval. ESs that did not fulfill these three properties were not recognized as ES in our study, and were then classified as "Undetectable ES". "False negative" ESs were ESs possessing the three properties but that were not detected by the algorithm. False positive ESs were values that were wrongly detected as ESs by the algorithm. Additional Table 2 reports examples of Undetectable, False positive and False negative ESs. Secondly, performance testing was carried out at the abstract level. An abstract was considered as "well classified" if human recognition matched to the automated detection for all ESs, without any False positive or False negative ESs.

We iteratively improved the performance of the algorithm using random batches of 200 PubMed abstracts. This was done until an equilibrium point was reached, for which 1) further attempts to improve sensitivity impaired specificity, or vice versa and 2) no other change of the coding script could improve both sensibility and specificity.

At the final stage, data from two observers were merged. Specificity and sensitivity were respectively 99.9% and 95% for abstracts and 99.9% and 96.5% for ESs. Inter-evaluator agreement kappa was superior to 0.97. The list of all assessed PMID and classifications performed by User1 and User2 is available in Supplementary File 1.

Definitions of citation characteristics

Here is how we precisely defined the retrieved characteristics of each citation (see online data [41] for coding details):

- **Month/year of publication**: The year and month were contained inside the <Year> and <Month> tags, nested in <PubDate>. If these were unavailable, the <MedlineDate> tag nested in <PubDate> was used instead. In some cases, <MedlineDate> only provided the season and the year: the month was March for Spring, June for Summer, September for Autumn and December for Winter. In cases when, <MedlineDate> contained two years (e.g. 1991-1992), the first year was kept. When no month was available in <PubDate>, the xml tag <DateCreated> was considered. This tag corresponds to the date of the creation of the citation within Medline. So, if the year of <PubDate> was equivalent to the year provided by <DateCreated>, then the month provided by <DateCreated> was kept. If the Medline year was the following year, the month was considered as December. Otherwise, January was assumed. See Additional Fig. 1b ("Algorithm for year and month extraction")

- **Authors' affiliation country(ies)**: Using the database of the tool MapAffil [46] (linked to PMIDs), the different countries inside authors' affiliations were identified (Methods). For PMIDs unknown by MapAffil (10 502 abstracts), countries were retrieved from affiliation strings by text mining using country names (in full or using the 3-letter code) and states/provinces (for USA and Canada), using the current norm ISO-3166. See Additional Fig. 1b ("Countries and sub-continent extracted").

- **Medical SubHeadings keywords**: Mesh Headings were first detected using the 2017 Mesh database (MH code from the d2017.bin file). Variant entry terms were also identified (ENTRY code from the d2017.bin file). To improve detection, Mesh Headings and variants were also identified after pre-treatment of the chains of characters (all in lowercase, parentheses and punctuation replaced by space). The UMLS Metathesaurus (file MRCONSO.RRF) was used to obtain a list of lexical variants [47] and link them to the corresponding Mesh terms. See Methods and Additional Fig. 1b for more details.

- **Detection of a multivariate analysis**: for each abstract, the presence of multivariate analysis was identified using two mining strategies: 1) search for the presence of an adjusted ESs (e.g. "adjusted odds ratio", "ORa" or "aOR"), and 2) keyword searching of the term "adjusted", "multivariate" or "multivariable" in the abstract. An abstract was identified as reporting a multivariate analysis if conditions 1 or 1+2 were fulfilled.

- **Labeling as a citation from an Open Access Journal**. A citation was considered as being published in an Open Access (OA) journal: 1) if the International Standard Serial Number (ISSN) of the journal where the cited article was published was listed in the Directory of Open Access Journals [48] (https://doaj.org/), and/or 2) if its PMID could be associated with a PubMed Central Identifier (PMCID), and/or 3) if the citation was indexed in PubMed as a "free full-text" article.

- **Labeling as a citation from a Core Clinical Journal**: a citation was considered as being published in a Core Clinical Journal if the International Standard Serial Number (ISSN) of the journal where the cited article was published was listed in Abridged Index Medicus (https://www.nlm.nih.gov/bsd/aim.html).

- **Identification of the type of Confidence Interval**: a single confidence interval level was chosen from each abstract. If multiple levels were identified, the lowest value of precision level was chosen ("90%CI", "95%CI" then "99%CI"). If no level was identified, "95%CI" was assumed.

- **Labeling of a citation as a "Review"**: We attributed the "Review" status to a cited article if one of the terms "review", "systematic review" or "meta-analysis" were identified from title and/or MeSH keywords and/or free keywords and/or Publication Type in xml tags.

Supplementary Tables

Additional Table 1 - Check for polysemy of terms related to types of ESs

The algorithm checked for the polysemy of acronyms. Through the MediLexicon online database of pharmaceutical and medical abbreviations (http://www.medilexicon.com/), all potential synonyms were identified by text mining on the entire abstract. All the terms considered are presented below. From regular expressions, some variations were considered to increase the detection of ES acronyms: presence or absence of plural, hyphen or spaces. The presence of any of these terms in an abstract oriented the data mining process towards a more restrictive procedure, in order to minimize the “False positive” rate.

Type of ES	Terms considered as risk
**OR type**	
OR, ORs and ORa	Oculo-Respiratory Syndrome; Olfactory Reference Syndrome; Oral Rehydration Salt; Oral Rehydration Solution; Oral Surgery; Oral Surgeon; Orthopedic Research Society; Orthopedic Surgeon; Orthopedic Surgery; Orthopedic Research; Outer Root Sheath; Oxygen Radical Scavenger; Objective Remission; Objective Response; Odorant Receptor; Oestrogen Receptor; Olfactory Receptor; Operating Room; Opioid Receptor; Organ at Risk; Orienting Response; Ovulation Rate; Oil Retention; Open Reduction; Optic Radiation; Operation Research; Oroso-mucoid; Outcome Research; Rate of Outflow; Outflow Rate; Orbital; Ocular Residual Astigmatism; Office of Regulatory Affair; Opiate Receptor Agonist; Opioid Receptor Agonist; Opposite Ray Algorithm; Optical Rapid Assay; Outwardly Rectifying Astrocyte; Oxidoreductase Activity.
aOR and aORs	Acridine Orange Reaction; Adequate Ovarian Reserve; Aldehyde Ferredoxin; Oxido-Reductase; Aldehyde Oxido-Reductase; Alvarados Orthopedic Research; Aorta; Aortic Root; At Own Risk; Audio-Ocular Response; Auditory-Oculogyric Reflex; Occlusion and Reperfusion; Adult Onset Rheumatoid Arthritis; Onset Rheumatoid Arthritis.
POR, PORs and PORa	Patient Oriented Research; Physician Of Record; Porfiromycin; Porin; Portion; Post-occlusive Oscillatory Response; Post-occlusive Reduction; Post-rhinal Cortex; Problem Oriented Record; Protochlorophyllides Oxydoreductase; Protochlorophyllide Reductase; Pyruvate Ferrodoxin Oxidoreductase; Pyruvate Oxidoreductase; Probability Of Remission; Porphyrin; Proportional Operation Ratio; Psychiatric Outpatient Rating Scale; Porin A.
aPOR and aPORs	Apolipoprotein, Apoptosis Resistant, Appendiceal Orifice; Association Of Patients Oriented Research.
PR, PRs, PRa, PRRs and PRR	Per rectum; Pulse Rate; Pagetoide Retikulose; Palindromic Rheumatism; Parallax Refraction; Partial Reinforcement; Partial Remission; Partial Response; Partial Responder; Particular Respirator; Peer review; Penrose Drain; Perfusion Rate; Peripheral Resistance; Pesticide Regulation; Phenol Red; Photoreaction; Physical Rehabilitation; Polyarthrite Rhumatoïde; Posterior Root; Postural Reflexes; Potency Ratio; Precipitation Radar; Preferences Records; Pregnancy Rate; Pre-retinal; Pressoreceptor; Pressures; Pregnancy; Prevention Research; Preyer Reflex; Proctology; Production Rate; Profile; Progesterone Receptor; Progress Report; Progressive Relaxation; Progressive Resistance; Prolactin; Prolonged Remission; Prospective Reimbursement; Protein; Public Relation; Pulmonary Rehabilitation; Pulmonic Regurgitation; Pulse Repetition; Pyramidal Response; Prednisolone; Pair; Physical Rehabilitation; Pityriasis Rosea; Postmyalgia Rheumatica; Prolin-Rich; Propanolol; Prosthion; Praseodymium; Presbyopia; Prism; Propyl; Parry Romberg Syndrome; Plasma Renin Substrate; Personality Rating Scale; Pharmaceutical

Type of ES	Terms considered as risk
	Reimbursement Section; Phosphoribosyl pyrophosphate Synthetase; Photon Radiosurgery System; Pierre Robin Syndrome; Post-radiation Sarcoma; Post-reperfusion Syndrome; Post-ribosomal Supernatant; Pressure; Prevention Research Synthesis; Procto-recto-sigmodoscopy; Producer Retailer; P-Receptor; Pharmaceutical Representative; Photo-receptor; Primitive Reflex; Progestin Receptor; Proteases ; Panel Reactive Antibody ; Paperwork Reduction Act; Participatory Rural Appraisal; PCR Restriction; Peripheral Renin Activity ; Pharmacy Restructuring Authority ; Phosphoribosylamine; Physician Recognition Award; Plaque Reduction Assay; Plasma Renin Activity; Polymorphism Analysis; Positive Relative Accommodation; Prazosin; Prerenal Azotemia; Probabilistic Risk Analysis; Progesterone Receptor Assay; Progressive Retinal Atrophy; Pathogen Recognizing Receptor; Plasma Refilling Rate; Plasma renin Reactivity; Platelet Retention Rate; Poliovirus Receptor Related; Post-radiation Recurrence; Post-replication Repairs; Post-repolarization Refractoriness; Pre-readiness Review; Prolin Rich Region; Proline Rich Repeat; Promoter Recognition Region; Proportional Reporting Ratio; Protective Response Recommendation; Proton Relaxation Rate; Proximal Regulator Region; Pulmonary Reimplantation Response; Pulse Repetition Rate; Porcine Reproductive Respiratory Syndrome; Proline Rich Sequence; Positive Regulator Region; Psychomotor Retardation Rating Scale.
aPR, aPRS and aPR	Abdomino-perineal Resection; Absolute Production Rate; Absolute Proximal Reabsorption; Accessory Planta Retractor; Acute Phase Reactant, Acute Phase Reaction; Acute Phase Response; Advance Production Release; Amebic Prevalence Rate; Anatomic Porous Replacement; Anterior Pituitary Reaction; Aprotinin; Automatic Pressure Relief; Ampicillin Resistant; Acute Pain Relief Service; Acute Psychiatric Rating Scale; Acute Phase Rabbit Serum; Allelic Polymorphic Region; Automatism Preserved Responsiveness.
**RR type**	
RR, RRs and RRa,	Respiratory Rate; Radiation Research Society; Raman Spectroscopy; Ras Recruitment System; Recovery Of RNA Synthesis; Rep Recognition Sequence; Resonance Raman Scattering; Resonance Rayleigh Scattering; Retro-rectal Space; Richard Rundle Syndrome; Recruiting Response; Resistance Ratio; Respiratory Rate Response Rate; Respiratory Resistance; Respiratory System; Radiation Reaction; Radiation Response; Rational Recovery; Recovery Room; Reflejo-Rotulianos; Relative Response; Renin Release; Respiratory Reserve; Response Rate; Result Reporting; Retinal Reflux; Rheumatoid Rosette; Ribonucleotide Reductase; Riva-Rocci; Radio-iodine Remnant Ablation; Radioligand Receptor Assay; Right Radial Artery; Right Renal Artery; Rural Appraisal; Radio-Receptor Activity; Radio-Receptor Analyses; Radio-Receptor Assay; Radio-Receptor binding Assay; Retro-Rubral Area; Reaction Rate Analyser; Receptor Radioligand Assay; Registered Record Administrator; Registered Restorative Assistant; Renal Renin Activity.
aRR and aRRs	Absolute Risk Reduction; Accelerating Rotarod; Achievable Risk Reduction; Acute Reaction Rejection; Age-Related Resistance; Airway Retention Ratio; Aldosterone Plasma Renin; Aldosterone Renin Ratio; Antigen Retaining Reticulum; Aortic Root Replacement; Arrhythmia Arrestin; Arsenate Reductase.
IRR, IRRa, IRRs	Insulin Receptor Related Receptor; Inter-Rater Reliability; Increased Radio-Resistance; Induced Radio-Resistances; Insulin Related Receptor; Internal Rate Return; International Reference Reagent; Intra-Renal Reflux; Inverted Repeat; Ionising Radiation Regulations; Iron Response Regulators; Inspiratory Rrs; Interleaved Relaxation Recovery Space; Infrared Reflection Absorption; Isolated Rubbed Rat Aorta.
**HR type**	
HR, HRs and HRa	Human Recombinant Afgf; Health Record Analysis; Health Risk Appraisal; Health Risk Assessment; Heart Rate Audiometry; Heart Reactive Antibody; Heidelberg Retina Angiograph; Hereditary Renal Adysplasia; High Right Atrium; Histamine Release Activity; Histamine Release Assay; Hormone Receptor Analysis; Human Resource Administration; Health Resource Administration; Human Ovarian Cancer Cell; Human Reliability Analysis; Human Reliability Assessment; Harvey Ras; Hamilton Anxiety Rating Scale; Health Risk Assessment Instrument; Hamman Rich Syndrome; Hamilton Rating Scale; Hazard Ranking System; Health and Rehabilitative Service; Health Retirement Study; Hepato-Renal Syndrome; High Rate Stimulation; Histidyl-T-Rna Synthetase; Hodgkin Reed Sternberg; Hormone Receptor Site; Humero-Radial Synostosis; Hyper-Radio Sensitivity; Hallux Rigidus; Hamman Rich; Heart Rate; Heating Rate; Hematologic Response; Hemi-Rectococcygeus; Hemorrhagic Retinopathy; Heterosexual Relation; High Resolution; High Risk; Higher Rate; Histamine Receptor; Hormonal Response; Hospital

Type of ES	Terms considered as risk
	Record; Hospital Report; Howship Romberg Syndrome; Human Resource; Hydroxy-ethyl-rutinoside; Hyper-immunes Reaction; Hypophosphatemic Ricket; Hypoxic Responder; Hairless; Host Range; Human Recombinant; Heart Reactive; Homologous Region.
aHR and aHRs	Abacavir Hypersensitivity Reaction; Acute Humoral Rejection; Airway Hyperreactivity; Airway Hyper-Responsiveness; Alcohol-based Hand Rub; Anti-Hyaluronidase Reaction; Aqueous Homogeneous Reactor; Assisted Human Reproduction; Association of Health Record; Atrial Heart Rate; Ah Receptor; Aromatic Hydrocarbon Receptor; Aryl Hydrocarbon Receptor; Army Human Resource; Army Human Resource Strategy.

Additional Table 2 - Examples of "undetectable" ESs, false negative ESs and false positive ESs

Category	PMID	Quotations	Explanation
Undetectable ES[a]	24992544	*"the risk to have pathological FEV1/FVC was about two times higher than in normal-weight patients. In overweight and obese asthmatic patients the probability of allergy was, respectively, 3.5 times (OR, 0.285) and 4.5 times (OR, 0.224) lower compared with normal-weight asthmatic patients".*	Risk was expressed as an increase of risk, without confidence interval.
	12534844	*"Compared with older persons consuming seven or fewer drinks per week, those exceeding the higher threshold of excessive drinking were more likely to have impairments in IADLs (adjusted odds ratio (AOR) = 8.4) and, to a lesser extent, AADLs (AOR = 3.7)".*	No confidence interval was found.
	9972719	*"There was no statistically significant association between overall satisfaction scores and the following factors: (1) length of wait in the clinic (OR, 2.7; p = 0.0747)".*	No confidence interval was found (the p-value was not considered as sufficient)
	15194716	*"[…] speeds (adjusted odds ratios (aOR) were 1.24, 1.52, and 1.66 in built up areas, on rural roads, and on motorways respectively), to report driving while under the influence (aOR = 1.39)".*	No confidence interval was found
	26275417	*"Patients with splice-site or missense mutations had lower mortality than patients with truncating mutations (OR 0.459, 95% CI 0.213 to 0.990, and OR 0.196, 95% CI 0.213 to 0.990, respectively)".*	Wrong confidence interval limits: 0.196 not between 0.213 and 0.990.
	26363843	*"no impact on overall mortality (HR 1.04 CI 0.99-1.02;), but with increased mortality for patients presenting with STEMI (HR 1.14; CI 1.06-1.23;). The propensity analysis was consistent with these findings".*	Wrong confidence interval limits: 1.04 not between 0.99 and 1.02.
	18055733	*"Beliefs that testing would cause unnecessary distress in pregnancy (aOR 0.3, 95% CI 0.1-0.7), or that testing was not worth the expense (aOR 0.1, 95% CI 0.0-0.6) were associated with not testing".*	A confidence limit was set to 0, the ES was not detected by the algorithm.
	26338316	*"However, both groups had similar rates of mortality (OR: 0.91; 95%CI: 0.91-1.51; p=.73), appropriate therapy".*	The lower bound of the confidence interval was equal to the value of ES.
	25695003	*"History of both diagnostic [OR=3.06 (95% CI: 1.32-7.06)] and therapeutic [OR=7.54 (95% CI: 1.5935.76) X-ray radiations were significantly higher".*	Wrong confidence interval limits: a hyphen was probably missing between 1.59 and 35.76; the ES was not detected.
False negative	7932822	*"Among women who had been pregnant at least once, the risk of breast cancer in those who had experienced an induced abortion was 50% higher than among other women (95% CI = 1.2-1.9)".*	Risk was expressed as a proportion of risk.
	20471772	*"higher prevalence of aortic repair (79% vs 6%; P < .001). Multivariate analysis selected no attempt at aortic repair (odds ratio [OR], 90.9; 95% confidence interval [CI], 10.6-1000)".*	A confidence limit was above or equal to 1000, the ES was not detected by the algorithm.
	21695525	*A low-risk MPS is associated with a low risk of cardiac events whereas high-risk MPS carries a 4.8-fold increased risk, 95% CI [3.2 - 7.2] (p < 0.0001).*	No ES was detected because no ES type was found within abstract.
False positive	21697852	*When possible confounders were entered into the multivariate logistic regression model, the independent predictors of frontal lobe dysfunction were eGFR (odds ratio 0.854; 95% confidence interval (CI) 0.743-0.983 per 10 ml min(-1) per 1.73 m(2)) and the number of lacunar infarction (odds ratio 1.460; 95% CI*	1.376 (95% CI 1.301-1.451) was wrongly detected as an Odds Ratio.

Category	PMID	Quotations	Explanation
		1.127-1.892). The mean of the logarithmically transformed Stroop test scores in the eGFR <60 ml min(-1) per 1.73 m(2) group was 1.376 (95% CI 1.301-1.451)	
	26357898	*"The pooled sensitivity, specificity, diagnostic odds ratio, positive likelihood ratio and negative likelihood ratio were 0.86 (95% CI: 0.79-0.91), 0.78 (95% CI: 0.68-0.85), 22 (95% CI: 10-48), 3.8 (95% CI: 2.6-5.7), and 0.18 (95% CI: 0.11-0.28), respectively. The area under the summary receiver operator characteristic curve was 0.89 (95% CI: 0.86-0.92)".*	Sensitivity, specificity, likelihood ratio and area under the summary receiver operator characteristic curve were misidentified as an odds ratio.
	26275060	*"for surgical failure and hypotony [hazard ratio (HR), 2.92, 6.64; 95% confidence interval (CI), 1.22 to 7.03, 1.47 to 30.0; P = 0.018, 0.014, respectively], and vitrectomy after trabeculectomy for surgical failure with or without hypotony criteria (HR, 2.32, 4.06; 95% CI, 1.02 to 5.28, 1.30 to 12.7; P = 0.045, 0.016, respectively)".*	No correct detection of hazard ratio values. Instead, the algorithm extracted HR = 6.64 [1.22, 7.03] and HR = 4.06 [1.02, 5.28] because of the "respectively" (false positives and false negatives).

[a] Detectable ES was defined as 1) an OR/RR/HR type of ES, 2) whose value was strictly positive and 3) bounded by a confidence interval [8].

Additional Table 3 - Mathematical transformations and main outcomes

Additional Table 3a - Mathematical transformations

#Transform	Mathematical description	Ref	Numerical examples	"Standardization" ⇒ Allow combination of "protective" and "risk" values	"Normalization" ⇒ Allow calculations in a linear metric[a]
T#0	No transformation	-	$ES = 2 \Rightarrow ES = 2$	No	No
T#1	$ES' = \begin{cases} ES \; if \; ES \geq 1 \\ \frac{1}{ES} \; if \; ES < 1 \end{cases}$	-	$ES = 2 \Rightarrow ES' = 2$	Yes	No
		[49]	$ES = 0.5 \Rightarrow ES' = 2$		
T#2	$ES'' = \ln(ES)$	[50]	$ES = 2 \Rightarrow ES'' = 0.69$	No	Yes
			$ES = 0.5 \Rightarrow ES'' = -0.69$		
T#3	$ES''' = \lvert\ln(ES)\rvert$	[50]	$ES = 2 \Rightarrow ES''' = 0.69$	Yes	Yes
			$ES = 0.5 \Rightarrow ES''' = 0.69$		

[a] ESs are on a logarithmic scale (two opposite directions: between 0 and 1 for "protective" values", and between 1 and +∞ for "risk" values), their values are put into a canonical form in order to allow calculations of common trends [49].

Additional Table 3b - Main outcomes

#Outcome	Statistical Unit	Description of the outcome	Variable
O#0	Extracted ES	Original value of extracted ES	Quantitative continuous
O#1	Abstract	Minimal ES value per abstract (i.e. within a single abstract, the nearest value to 1)	Quantitative continuous
O#2	Abstract	Maximal ES value per abstract (i.e. within a single abstract, the farthest value from 1)	Quantitative continuous
O#3	Abstract	Mean of all ESs values per abstract (only after log transformation of original ESs) (transform T#3)	Quantitative continuous
O#4	Abstract	Magnitude of the ES with the narrowest 95%CI per abstract (after log transform of lower and upper bounds) (T#2)	Quantitative continuous
O#5	Abstract	Magnitude of the ES with the largest 95%CI per abstract (after log transform of lower and upper bounds) (T#2)	Quantitative continuous
O#6	Abstract	Mean magnitude of all ES 95%CI per abstract (after log transform of original lower and upper bounds) (T#2)	Quantitative continuous
O#7	Abstract	Proportion of statistically significant ES(s) per abstract (defined as an ES whose 95%CI does not include 1).	Percentage
O#8	Abstract	Presence of at least one statistically significant ES	Dichotomous (yes/no)

Additional Table 4 - Summary table of the analysis plan

A0. Preprocessed data
26 759 399 citations with 16 820 871 (63%) abstracts available.
Data presented in A, B, C and D concerned exclusively the period 1990-2015.

**(A) Descriptive analysis of the comprehensive database**	**Results**
1. Number of Pubmed citations (with or without available abstract)	16 620 702
2. Number of abstracts (with or without detected ESs)	13 322 754
3. Number of abstracts with at least one detected ES (% from A2)	283 310 (2.13%)
4. Number of detected ESs	814 120
5. Number of abstracts (n1) and ESs (n2) by type of Confidence Interval (90%, 95%, 99%)<br>Relative evolution by type of Confidence Interval (90%, 95% or 99%)	90%CI : n1 = 644 ; n2 = 1615<br>95%CI : n1 = 282 087 ; n2 = 810 575<br>99%CI : n1= 579 ; n2 = 1930<br>Additional Fig. 2i
6. Number of abstracts (n1), (% from A3), and number of ESs (n2) labeled as "Review"<br>Relative evolution of "Review" abstracts	n1= 34 846 (12.3%); n2= 119 563<br>Additional Fig. 2h

**(B) Descriptive analysis of ESs in abstracts** ("Review", "90%CI" and "99%CI" excluded)	**Results**
1. Number of abstracts (with or without ESs)	11 706 283
2. Number of abstracts with at least one detected ES (% from B1)	247 339 (2.1%)
3. Evolution of the number of abstracts with at least one detected ES	Additional Fig. 2a
4. Number of detected ESs	691 302
5. Number of abstracts with at least one ES of type "OR"	163 145
6. Number of abstracts with only ES(s) of type "OR" (% from B2)	157 141 (63.5%)
7. Number of abstracts with at least one ES of type "RR"	37 847
8. Number of abstracts with only ES(s) of type "RR" (% from B2)	34 413 (13.9%)
9. Number of abstracts with at least one ES of type "HR"	53 569
10. Number of abstracts with only ES(s) of type "HR" (% from B2)	48 616 (19.7%)
11. Number of abstracts with multiple types of ESs (% from B2)	7169 (2.9%)
12. Relative evolution of abstracts by type of ESs	Additional Fig. 2b
13. Number of ES(s) per abstract (median [Q1; Q3] and mean (SD))	2 [1;4] and 2.79 (1.96)
14. Number of ESs >1, ESs<1 or ESs=1	ES>1 ; n = 525 811<br>ES<1 ; n = 162 385<br>ES=1 ; n = 3106<br>Additional Fig. 3
15. Number of abstracts with all ESs >1 (% from B2)	161 013 (64.7%)
16. Number of abstracts with all ESs <1 (% from B2)	38 950 (15.8%)

17.	Number of abstracts with both ESs >1 and ESs <1 (% from B2)<br>Number of abstracts with ES =1 (% from B2)	45 524 (18.8%)<br>2852 (1.1%)
18.	Relative evolution of abstracts with all ESs >1, all ESs <1, and both	Additional Fig. 2c
19.	Number of abstracts with at least one “tiny” statistically significant ES (% from B2)	6745 (2.7%)
20.	Number of abstracts with at least one “large” statistically significant ESs (% from B2)	64 089 (25.9%)
21.	Number of abstracts with at least one identified continent of affiliation (% from B2)	238 954 (96.6%)
22.	Number of abstracts with at least one affiliation from Africa (% from B2)	7136 (3.0%)
23.	Number of abstracts with all detected affiliations from Africa (% from B22)	4737 (66.4%)
24.	Number of abstracts with at least one affiliation from North America (% from B2)	91 867 (38.5%)
25.	Number of abstracts with all detected affiliations from North America (% from B24)	82 463 (89.8%)
26.	Number of abstracts with at least one affiliation from South America (% from B2)	8771 (3.7%)
27.	Number of abstracts with all detected affiliations from South America (% from B26)	7418 (84.6%)
28.	Number of abstracts with at least one affiliation from Asia (% from B2)	48 202 (20.2%)
29.	Number of abstracts with all detected affiliations from Asia (% from B28)	43 650 (90.6%)
30.	Number of abstracts with at least one affiliation from Europe (% from B2)	87 958 (36.8%)
31.	Number of abstracts with all detected affiliations from Europe (% from B30)	80 307 (91.3%)
32.	Number of abstracts with at least one affiliation from Oceania (% from B2)	9692 (4.1%)
33.	Number of abstracts with all detected affiliations from Oceania (% from B32)	7943 (82.0%)
34.	Number of abstracts with cross-continental affiliations (% from B2)	12 436 (5.2%)
35.	Relative proportion of abstracts by continent of affiliation (from B2)<br>Relative evolution of abstracts by continent of affiliation (from B2)	Fig. 1a<br>Additional Fig. 2d
36.	Number of abstracts by field of research (from B2)	Fig. 2, Fig. S4
37.	Number of abstracts with at least an adjusted ES (% from B2)	53 544 (21.7%)
38.	Number of abstracts identified as reporting a multivariate analysis (% from B2)<br>Relative evolution of abstracts identified as reporting a multivariate analysis	136 724 (55.3%)<br>Additional Fig. 2e
39.	Number of abstracts labeled as citation with free full-text available (% from B2)<br>Relative evolution of abstracts published as free full-text	92 040 (37.2%)<br>Additional Fig. 2f
40.	Number of abstracts labeled as citation from Core Clinical Journals (CCJ) (% from B2)<br>Relative evolution of abstracts considered as being published in a CCJ	43 450 (17.6%)<br>Additional Fig. 2g

**(C)**	**Temporal evolution of ESs and their significance**	**Results**	**#O[a]**	**#T[b]**
1.	Median value of ESs<1 in 1990-1995 (from B14)<br>Median values of [lower limits of 95%CI; upper limits of 95%CI] in 1990-1995 (ESs<1)<br>Median value of ESs<1 in 2010-2015 (from B14)<br>Median values of [lower limits of 95%CI; upper limits of 95%CI] in 2010-2015 (ESs<1)<br>Median value of ESs>1 in 1990-1995 (from B14)<br>Median values of [lower limits of 95%CI; upper limits of 95%CI] in 1990-1995 (ESs>1)<br>Median value of ESs>1 in 2010-2015 (from B14)<br>Median values of [lower limits of 95%CI; upper limits of 95%CI] in 2010-2015 (ESs>1)	0.59<br>[0.32; 0.95]<br>0.63<br>[0.42; 0.91]<br>2.50<br>[1.23; 4.96]<br>2.11<br>[1.21; 3.54]	0	0
2.	Evolution of monthly distribution of ESs	Fig. 3a	0	1

3. Evolution of monthly medians of ESs	Fig. 3b	1	3
4. Evolution of monthly medians of ESs	Fig. 3b	2	3
5. Evolution of monthly medians of ESs	Fig. 3b	3	3
6. Evolution of monthly medians of ESs >1 and monthly medians of ESs<1	Additional Fig. 5a, Additional Fig. 5b	3	3
7. Evolution of monthly medians of ESs by type of ESs (from B6, B8, B10)	Additional Fig. 5c	3	3
8. Evolution of monthly median values of 95%CI amplitude	Fig. 3c	4	2
9. Evolution of monthly median values of 95%CI amplitude	Fig. 3c	5	2
10. Evolution of monthly median values of 95%CI amplitude	Fig. 3c	6	2
11. Evolution of monthly proportion of abstracts with at least one statistically significant "large" effect	Additional Fig. 5d	8	0
12. Evolution of monthly proportion of abstracts with at least one statistically significant "tiny" effect	Additional Fig. 5d	8	0
13. Evolution of monthly mean proportions of statistically significant ESs per abstract	Fig. 4b	7	0
14. Evolution of monthly mean proportions of statistically significant ESs (from B14)<br>Evolution of monthly mean proportions of statistically significant ESs (from B15, B16, and B17)	Additional Fig. 7a<br>Additional Fig. 7b	7	0
15. Evolution of monthly mean proportions of statistically significant ESs per abstract, by type of ESs	Additional Fig. 7c	7	0
16. Evolution of proportion of abstracts with at least one statistically significant ES	Fig. 4a	8	0

**(D) Factors associated with ESs and their significance**			
1. Evolution of monthly medians of ESs by field of research	Fig. 2	3	3
2. Evolution of monthly proportion of statistically significant ESs by field of research	Additional Fig. 4	7	0
3. Median values of ESs by continent, 1990-2015	Fig. 3a	3	3
4. Evolution of monthly medians of ESs by continent	Additional Fig. 5e	3	3
5. Mean proportions of statistically significant ESs by continent, 1990-2015	Fig. 1b	7	0
6. Evolution of monthly mean proportions of statistically significant ESs by continent	Additional Fig. 7d	7	0
7. Evolution of monthly medians of ESs, according to presence of a multivariate analysis	Fig. 5a	3	3
8. Evolution of monthly mean proportions of statistically significant ESs according to presence of a multivariate analysis	Fig. 5b	7	0
9. Evolution of monthly medians of ESs, according to the "CCJ" status of the article	Fig. 5e	3	3
10. Evolution of monthly mean proportions of statistically significant ESs according to the "CCJ" status of the article	Fig. 5f	7	0
11. Evolution of monthly medians of ESs according to the open access status of the article	Fig. 5c	3	3
12. Evolution of monthly mean proportions of statistically significant ESs according to the open access status of the article	Fig. 5d	7	0

**(E) Knowledge checking**			
1. Inter-observers' kappa analysis for performance testing of the algorithm	$\kappa > 0.97$; Supplementary Methods, Supplementary File 1	-	-
2. Specificity and sensitivity of the algorithm	Specificity: >99.9%; Sensitivity: >95% Supplementary Methods, Supplementary File 1	-	-
3. Evolution of monthly medians of ESs for abstracts categorized as "Reviews"	Additional Fig. 6a	3	3
4. Evolution of monthly mean proportions of statistically significant ESs for abstracts categorized as "Reviews"	Additional Fig. 6d	7	0
5. Evolution of monthly medians of ESs per type of CI	Additional Fig. 6b	3	3
6. Evolution of the monthly mean proportions of statistically significant of ESs per type of CI	Additional Fig. 6e	7	0

7.	Number of PMC abstracts with at least one detected 95%CI ES<br>Number of PMC abstracts with at least one detected 95%CI ES (excluding “Reviews”)<br>Number of PMC abstracts with at least one detected 95%CI ES and full-text article available in XML format (excluding “Reviews”)	72 687<br>64 829<br>25 868	-	-
8.	Number of detected ESs in PMC Abstracts among E7<br>Number of detected ESs in PMC Results among E7<br>Number of detected ESs in PMC Tables among E7	188 711<br>135 542<br>589 743	-	-
9.	Distribution of detected ESs in PMC articles among E7	38 961 PMC articles with detected ESs in Abstracts only<br>3414 PMC articles with detected ESs in Abstracts and Tables<br>11 209 PMC articles with detected ESs in Abstracts and Results<br>11 245 PMC articles with detected ESs in Abstracts, Results and Tables	-	-
10.	Evolution of monthly medians of ESs for Abstracts, Results and Tables in PMC articles	Additional Fig. 6c	3	3
11.	Evolution of monthly mean proportions of statistically significant of ESs for Abstracts and Results in PMC articles	Additional Fig. 6f	7	0

[a] #O: Outcome (Additional Table 3b)
[b] #T: Transformation (Additional Table 3a)

Additional Table 5 - Geographical analysis

Rank	Country	Mean ES (T#3)	Significant ES (%)	Number of ESs	Number of abstracts
1	**Denmark**	0.58	0.76	18643	5901
2	Jersey	0.63	0.79	125	45
3	Luxembourg	0.65	0.89	170	54
4	**Sweden**	0.66	0.81	23281	7785
5	Iceland	0.66	0.82	732	240
6	**United Kingdom**	0.67	0.79	48782	16882
7	**Canada**	0.68	0.80	33145	11340
8	**United States**	0.69	0.80	236736	82134
9	**Norway**	0.69	0.82	9892	3396
10	Czech Republic	0.70	0.84	1307	519
11	Ireland	0.70	0.85	2426	799
12	Romania	0.70	0.83	643	235
13	Oman	0.70	0.79	312	121
14	Netherlands	0.72	0.77	27833	10052
15	Slovakia	0.72	0.79	416	156
16	Austria	0.72	0.81	3817	1475
17	**New Zealand**	0.73	0.80	3774	1234
18	Guinea-Bissau	0.73	0.67	333	97
19	**Australia**	0.73	0.82	25021	8537
20	Germany	0.73	0.81	21833	8036
21	Cyprus	0.74	0.91	170	61
22	Trinidad and Tobago	0.74	0.87	129	38
23	Estonia	0.74	0.87	533	175
24	Hungary	0.74	0.80	1452	577
25	United Arab Emirates	0.74	0.81	283	105
26	Belgium	0.75	0.82	4606	1677
27	China	0.75	0.91	39185	13634
28	Burkina Faso	0.75	0.87	210	81
29	Ukraine	0.76	0.85	209	71
30	Finland	0.76	0.83	12001	3992
31	Botswana	0.76	0.79	178	66
32	Russia	0.77	0.81	1141	422
33	Singapore	0.78	0.85	3230	1133
34	Qatar	0.78	0.89	302	102
35	Bulgaria	0.79	0.76	181	67
36	Switzerland	0.79	0.80	7907	2709
37	Poland	0.80	0.83	4492	1673
38	Israel	0.80	0.83	6500	2454
39	Georgia	0.81	0.86	492	156
40	Croatia	0.81	0.85	770	271
41	Zambia	0.82	0.84	633	219
42	Taiwan	0.82	0.89	15157	5607

Rank	Country	Mean ES (T#3)	Significant ES (%)	Number of ESs	Number of abstracts
43	Peru	0.82	0.85	929	338
44	Bangladesh	0.83	0.87	989	322
45	Lebanon	0.83	0.83	571	201
46	France	0.83	0.83	23719	8360
47	Mozambique	0.84	0.84	189	63
48	Greece	0.84	0.85	4153	1573
49	Malawi	0.84	0.85	619	207
50	Rwanda	0.84	0.84	152	54
51	Hong Kong	0.85	0.88	4157	1527
52	Lithuania	0.85	0.91	616	198
53	Italy	0.85	0.83	26630	9933
54	Philippines	0.86	0.86	266	85
55	Korea, Republic of	0.87	0.89	18326	7115
56	Kenya	0.89	0.84	1866	608
57	Japan	0.89	0.86	27692	10839
58	South Africa	0.89	0.84	4081	1372
59	Ghana	0.89	0.84	689	239
60	Albania	0.89	0.83	122	34
61	Chile	0.89	0.87	1169	425
62	Colombia	0.90	0.85	1347	431
63	Ecuador	0.90	0.85	261	80
64	Spain	0.90	0.86	24367	8764
65	Slovenia	0.91	0.78	534	225
66	Portugal	0.91	0.86	2405	854
67	Venezuela	0.91	0.83	102	46
68	Mali	0.92	0.80	203	72
69	Tanzania	0.92	0.86	1411	451
70	Uruguay	0.92	0.89	334	123
71	Nepal	0.93	0.86	647	201
72	Kuwait	0.93	0.90	286	108
73	Jamaica	0.93	0.85	307	99
74	Brazil	0.93	0.89	13904	4795
75	Iran	0.93	0.85	4924	1915
76	Zimbabwe	0.94	0.80	585	195
77	Guadeloupe	0.94	0.88	106	36
78	Malaysia	0.94	0.88	2028	655
79	Uganda	0.94	0.83	2187	669
80	Argentina	0.94	0.87	1737	649
81	Senegal	0.95	0.84	257	80
82	Costa Rica	0.95	0.81	165	52
83	Pakistan	0.95	0.86	1629	489
84	Viet Nam	0.96	0.84	605	218
85	Saudi Arabia	0.96	0.84	1579	568
86	India	0.97	0.84	8241	2933
87	Gambia	0.98	0.79	295	107
88	Turkey	0.98	0.87	5431	2312

Rank	Country	Mean ES (T#3)	Significant ES (%)	Number of ESs	Number of abstracts
89	Cuba	0.99	0.89	321	106
90	Cote d'Ivoire	0.99	0.86	209	79
91	Latvia	1.00	0.85	149	52
92	Tunisia	1.01	0.88	675	283
93	Morocco	1.01	0.84	371	123
94	Cambodia	1.02	0.85	153	47
95	Egypt	1.03	0.82	1171	453
96	Cameroon	1.04	0.84	502	147
97	Indonesia	1.05	0.84	630	213
98	Jordan	1.05	0.86	263	101
99	Niger	1.06	0.89	233	86
100	Thailand	1.07	0.87	4035	1412
101	Sri Lanka	1.07	0.87	324	101
102	Serbia	1.07	0.88	1208	373
103	Nigeria	1.08	0.86	2126	722
104	Mexico	1.09	0.88	4299	1612
105	Congo	1.09	0.89	271	79
106	Ethiopia	1.10	0.94	2920	811
107	Sudan	1.13	0.78	251	93
108	French Guiana	1.19	0.93	120	35

Only countries with at least 100 detected ESs were included in this geographical analysis. The countries in bold and underlined are the “Five eyes” countries (Australia, Canada, New Zealand, United Kingdom and United States) and the Scandinavian monarchies (Denmark, Sweden, and Norway). These 8 countries are ranked in the top 20.

Supplementary Figures

Additional Fig. 1 - Overview of the "Knowledge Discovery in Databases" (KDD) approach used in this study

Additional Fig. 1a – The different steps that compose the KDD process (adapted from Fayyad [40])

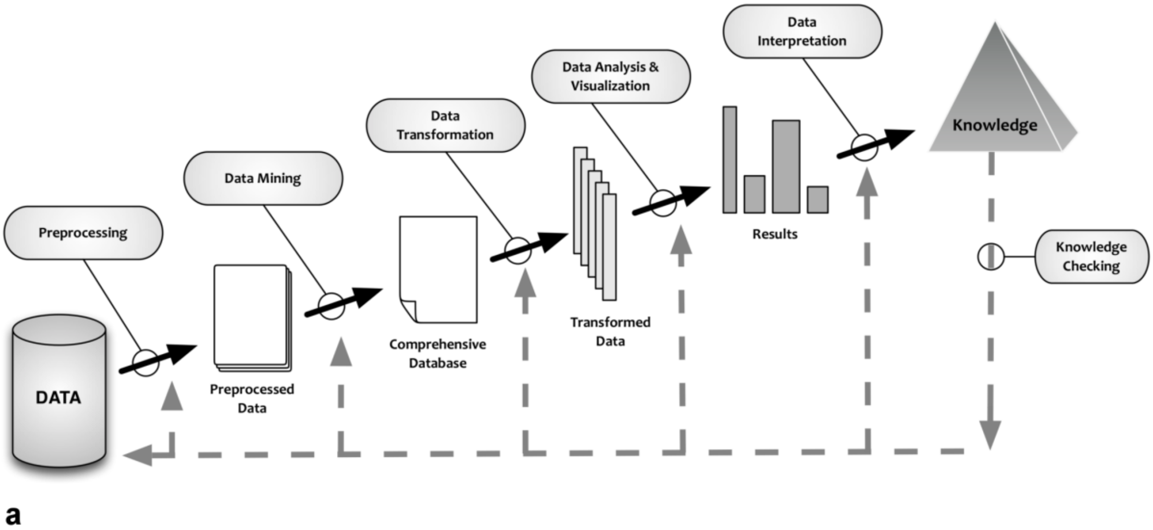

Additional Fig. 1b – Flowchart of the algorithm for PubMed data mining changer

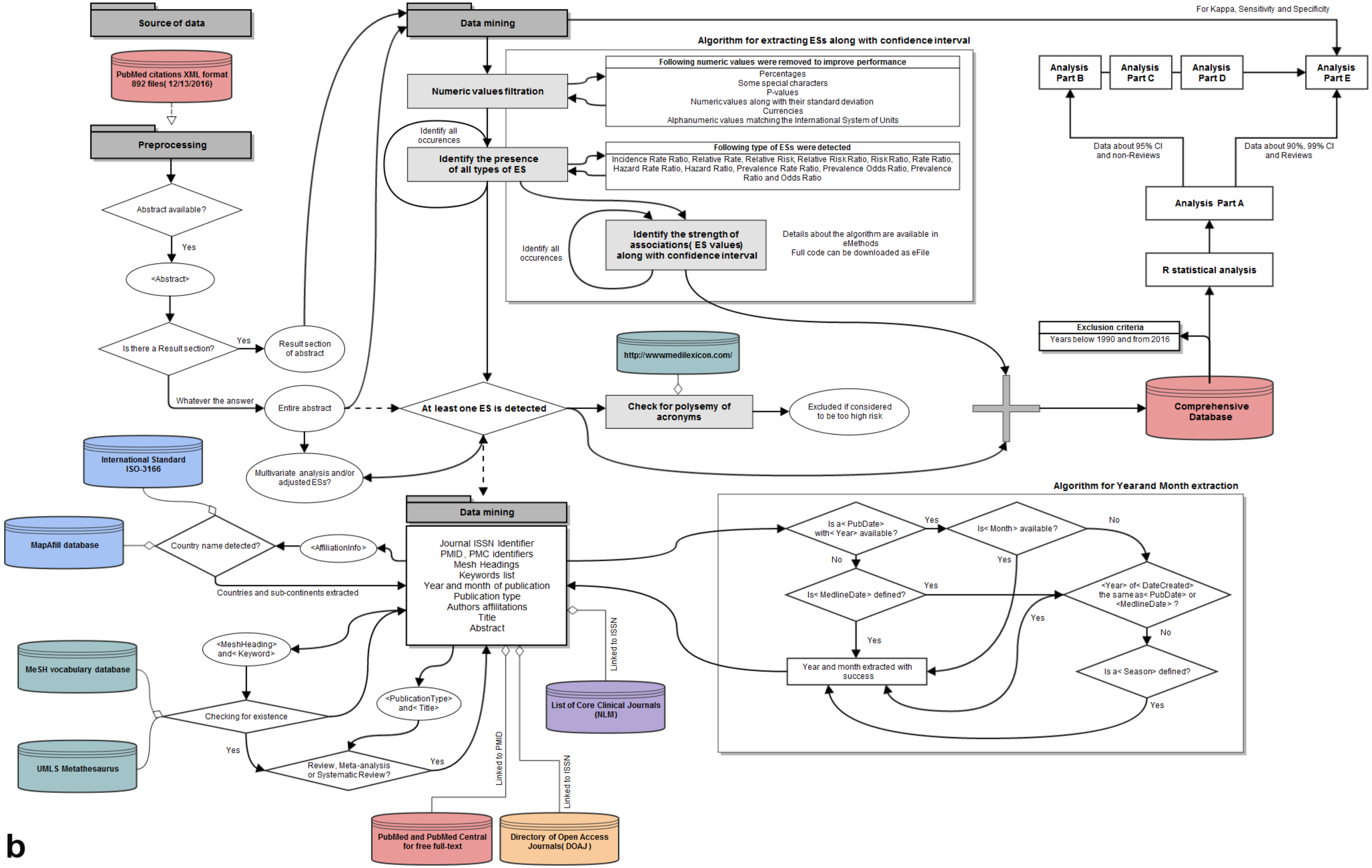

Additional Fig. 1c – Flow diagram of the selection process for abstracts (PubMed data mining)

Capital letters at the left of rectangles correspond to specific parts of the analysis plan detailed in Additional Table 4.

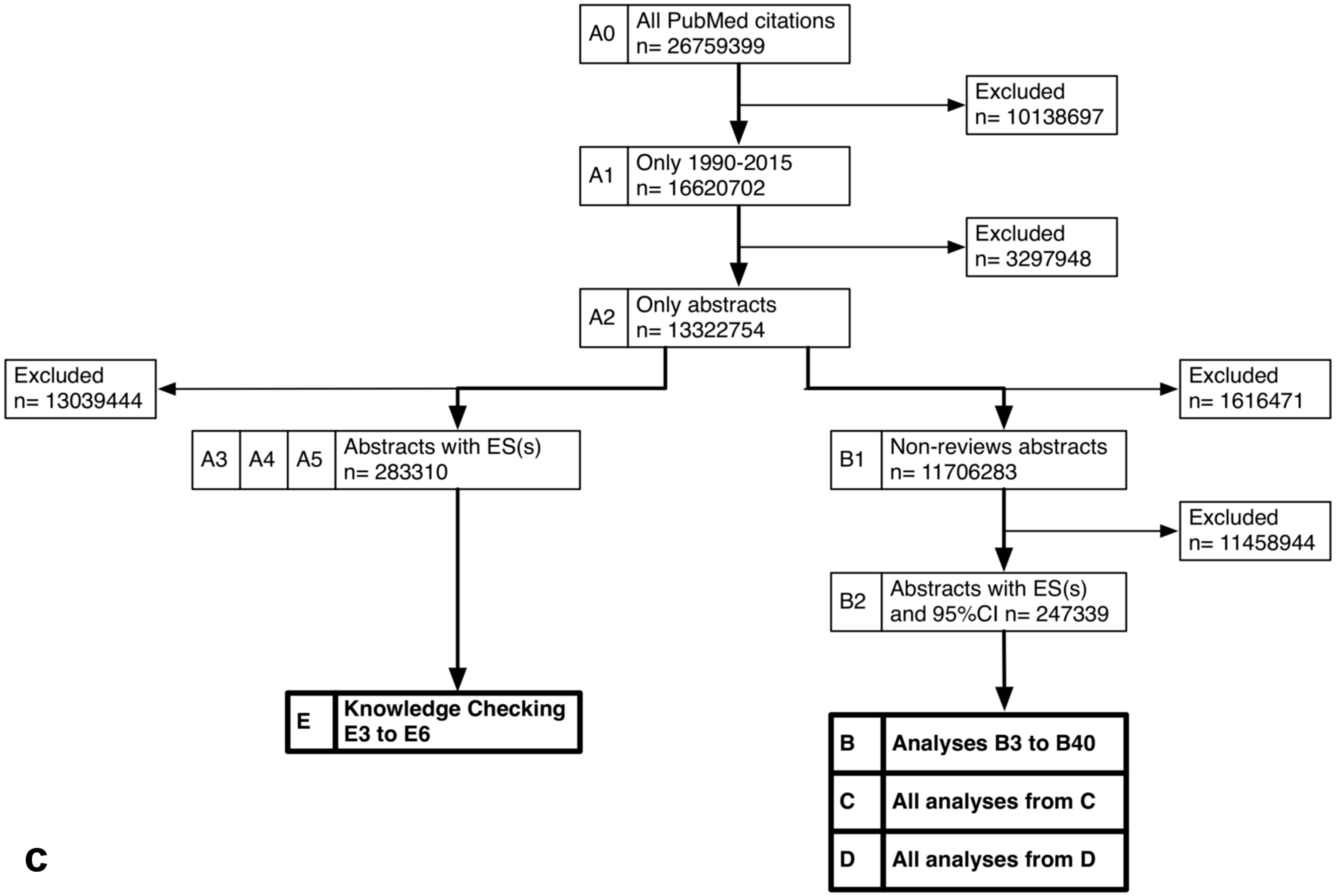

Additional Fig. 2 - Descriptive analysis of the comprehensive database and descriptive analysis of ESs in abstracts

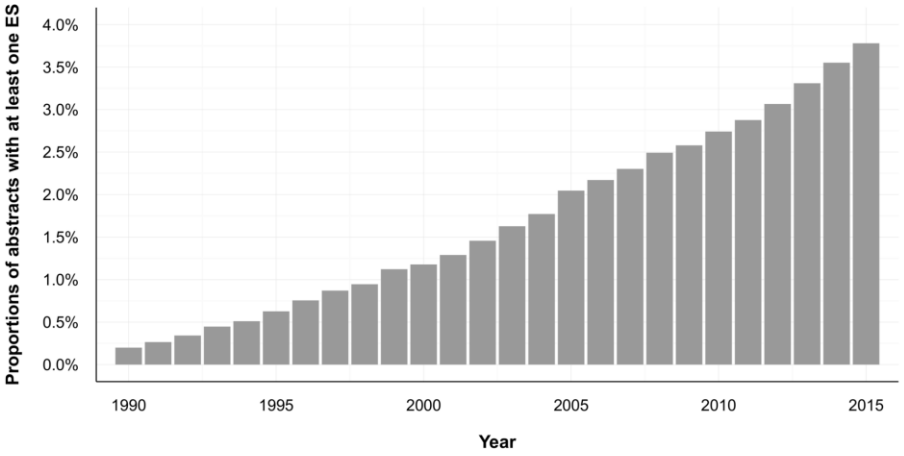

Additional Fig. 2a - Proportion of abstracts containing at least one ES also increased over time

The proportion of abstracts containing at least one ES is expressed relative to the total number of available abstracts (excluding reviews). Number of abstracts: 247339.

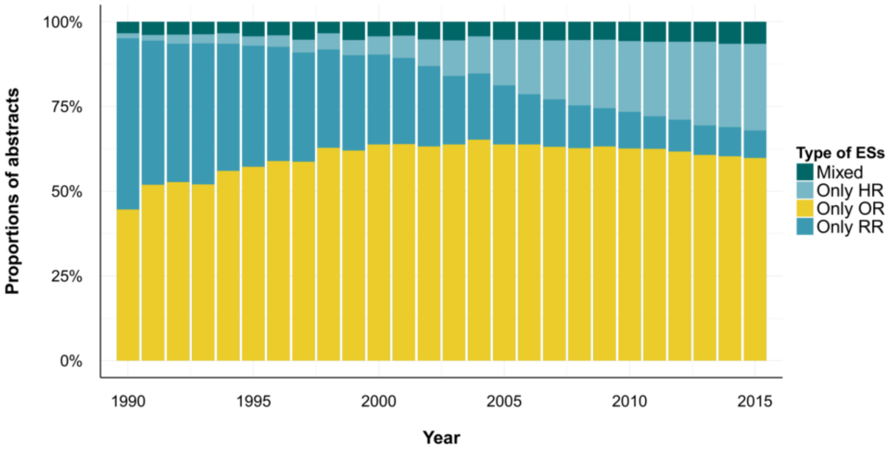

Additional Fig. 2b - A majority of OR and a trend for RR to be substituted by HR

Yearly distribution of the proportion of abstracts with exclusively Odds Ratio (OR), Relative Risk (RR) or Hazard Ratio (HR), or with multiple types of ESs (Mixed). There was trend for RR to be substituted by HR. The proportion of OR remained quite stable over time. Number of abstracts: 247339 (157141, 34413, 48616 and 7169 abstracts with only OR, only RR, only HR, and mixed, respectively).

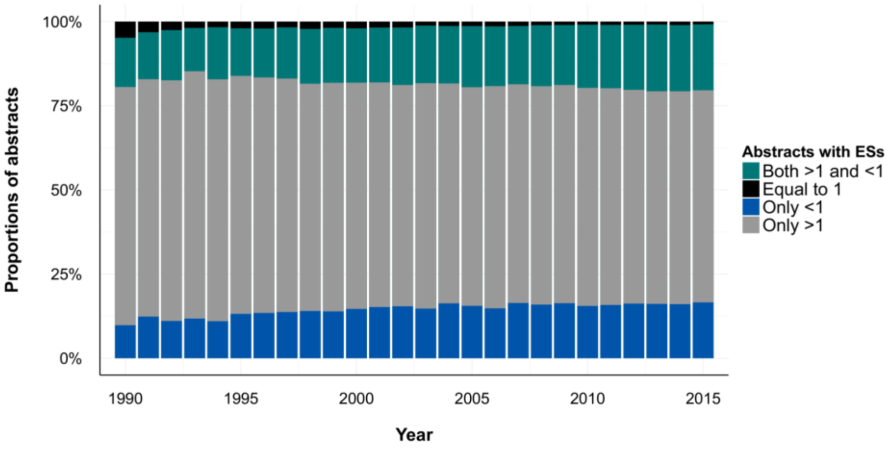

Additional Fig. 2c - A majority of abstracts with ESs >1

Histogram of the yearly distribution of the proportion of abstracts per type of ES values showed that ESs>1 still predominated largely, despite a modest increase of abstracts with all ES<1, and of those with a mix of (ESs>1 and ESs<1). Number of abstracts: 247339 (160013, 38950, 45524, 2852 abstracts with ESs only >1, only <1, both >1 and <1 and with ES equal to 1, respectively).

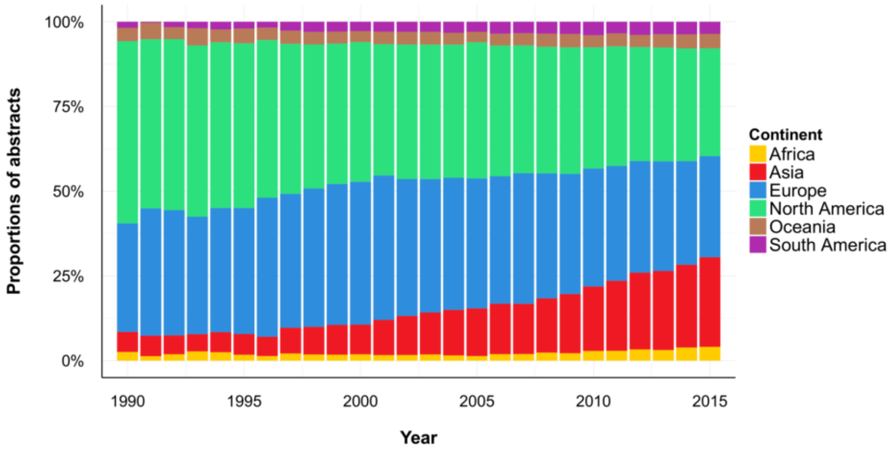

Additional Fig. 2d - Disparities between geographical areas: the weight of Europe and North America, the growth of Asia

Yearly distribution of the proportion of abstracts by continent. Europe and North America were by far the biggest providers of abstracts with ESs, although Asia was growing strongly. Number of abstracts: 238954 (7136, 48202, 87958, 91867, 9692 and 8771, and abstracts with Africa, Asia, Europe, North America, Oceania and South America, respectively). Abstracts with cross-continental affiliations (5.2% of abstracts) were counted in each continent concerned.

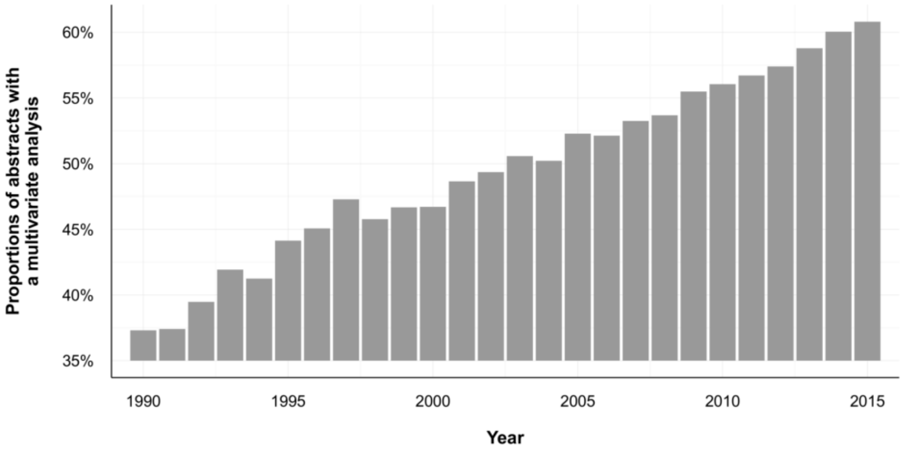

Additional Fig. 2e - The proportion of abstracts with a multivariate analysis increased with time

Yearly proportion of abstracts with at least one detected ES and containing a multivariate analysis is expressed relative to the total number of available abstracts with at least one detected ES (excluding reviews). Number of abstracts: 136724.

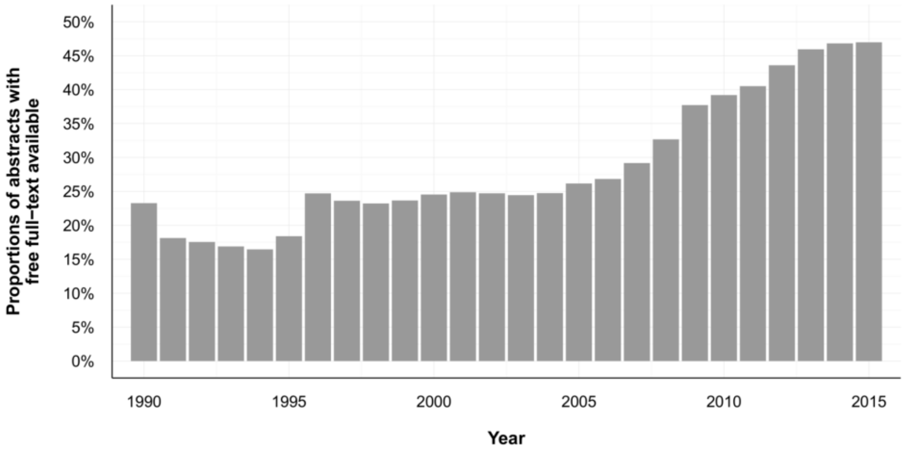

Additional Fig. 2f – The proportion of abstracts with a free full-text available increased with time

Yearly proportion of abstracts with at least one detected ES and with a free full-text available is expressed relative to the total number of available abstracts with at least one detected ES (excluding reviews). Number of abstracts: 92040.

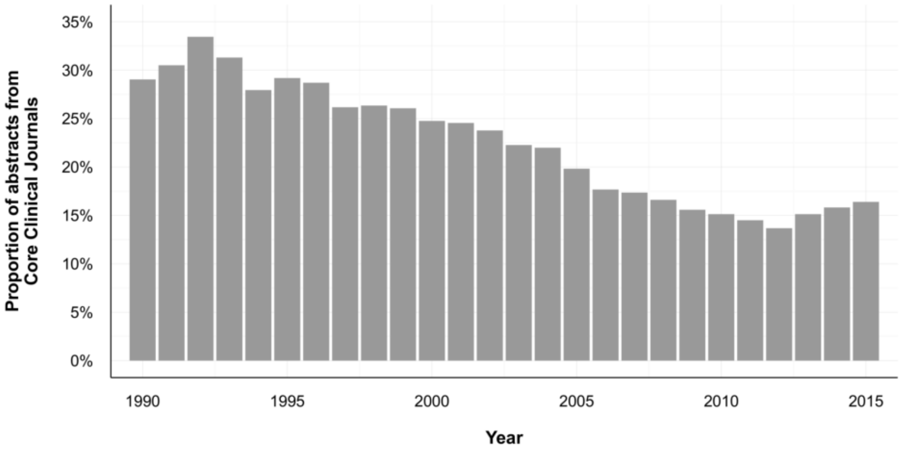

Additional Fig. 2g - The proportion of abstracts from Core Clinical Journals decreased with time

Yearly proportion of abstracts with at least one detected ES and from a Core Clinical Journal is expressed relative to the total number of available abstracts with at least one detected ES (excluding reviews). Number of abstracts: 43450.

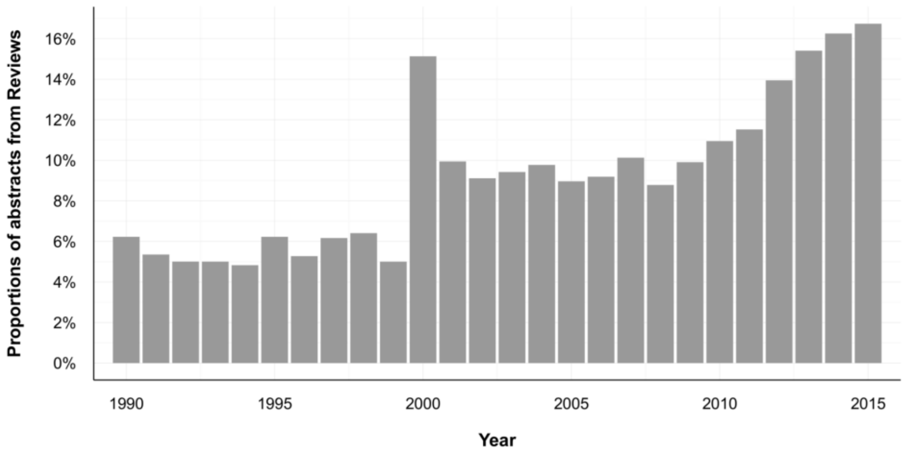

Additional Fig. 2h - Increase of the proportion of abstracts with at least one detected ES from Reviews

The histogram shows an increase of the proportion of abstracts with at least one detected ES, among abstracts labeled as “Reviews”. The peak observed in 2000 is unexplained, but might be attributed to a “millennium” effect. Number of abstracts: 34846.

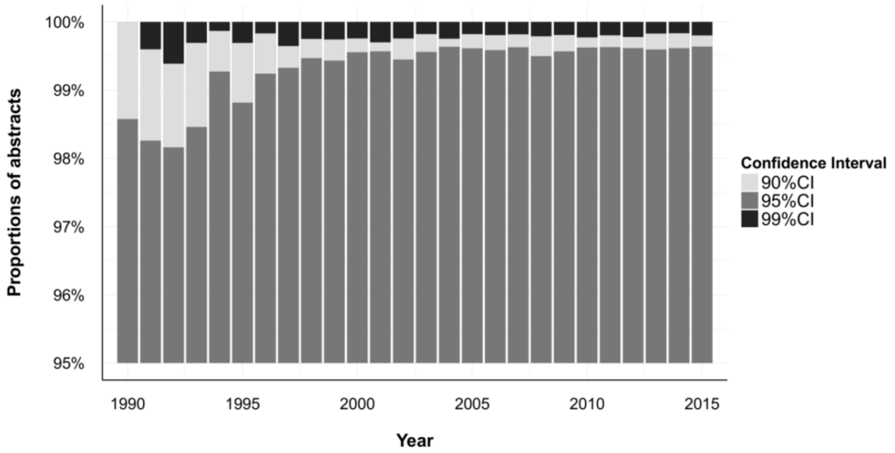

Additional Fig. 2i - 95%CI type represented the overwhelming majority of CIs, with no major evolution over time

The histogram represents, from the comprehensive database, the evolution of the yearly distribution of abstracts per type of confidence interval (90, 95 or 99% CI; if an abstract contained several types of CI, the lowest value of precision level was chosen). Number of abstracts: 283310 (644, 282087, 579 for 90%, 95% and 99%CI, respectively).

Additional Fig. 3 - Histogram distribution of ESs (T#2)

Histogram distribution of ESs (T#2) for risk values (in gray) and protective values (in blue). Dashed lines represent the respective medians (-0.48 and 0.79). There were more ESs>1 than ESs<1. Number of ESs: 691302.

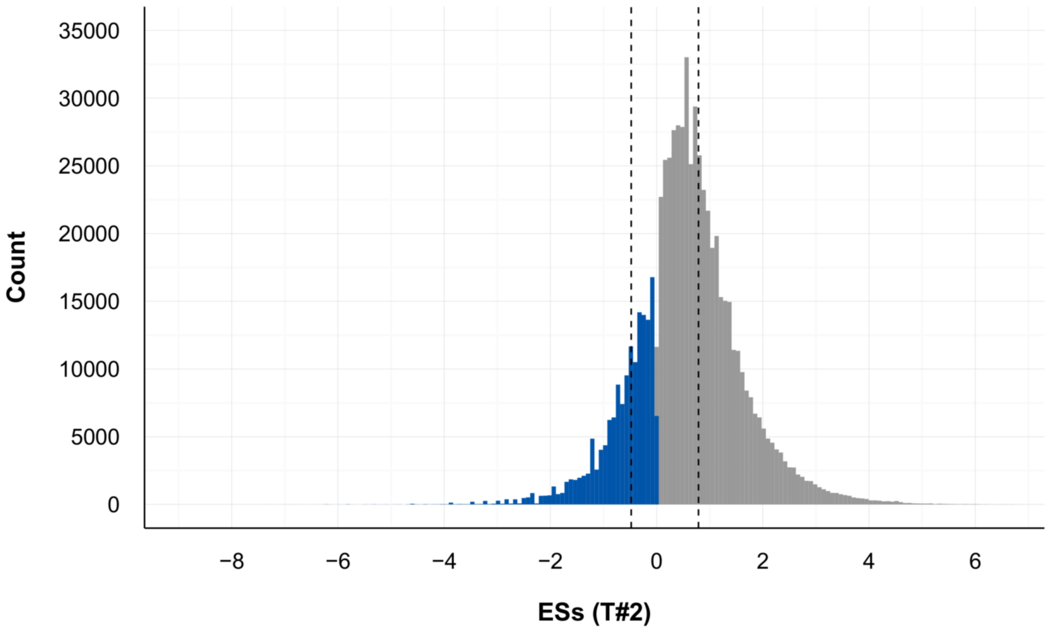

Additional Fig. 4 - Heatmap of the temporal evolution of proportion of statistically significant ESs per abstract: disparities among fields of research

Proportions of statistically significant ESs were considered at the abstract level. Abstracts were linked to specific research fields according to their (MeSH) keywords, so a single abstract could be linked to multiple fields of research (overall ratio= 801839/229581=3.49). Research fields (at the right of the figure) were defined from two branches of the MeSH Tree (US NLM): [C] “Diseases” and [G] “Phenomena and Processes”. Numbers in brackets are the total number of abstracts with at least one detected ES during the 25-year period in a specific research field. Three branches (out of 43) were eliminated because fewer than 1000 abstracts (containing at least one ES) were detected during the period. Trends were calculated at the monthly level, but are represented in the graph at the yearly level, for the sake of readability. The grayscale indicates yearly mean values of proportion of statistically significant ESs: lighter grey corresponds to lower proportions, and darker grey to higher proportions. At the left, research fields were grouped using a hierarchical cluster analysis and are represented as a dendrogram. The color scale indicates the Mann-Kendall τ value[44] of the evolution of monthly mean proportions of statistically significant ESs for each research field. White rectangles mean non-significant trends. Colored rectangles are blue (not red), with variable intensity, meaning a monotonic upward trend of ESs in nearly all research fields. The most marked increase over the years was observed for Neoplasms (τ=0.66, p<.001). Higher proportions of statistically significant ESs are found in fields dealing with infectious diseases (e.g. Microbiological Phenomena, Virus Diseases, Bacterial Infections and Mycoses, see top of the figure).

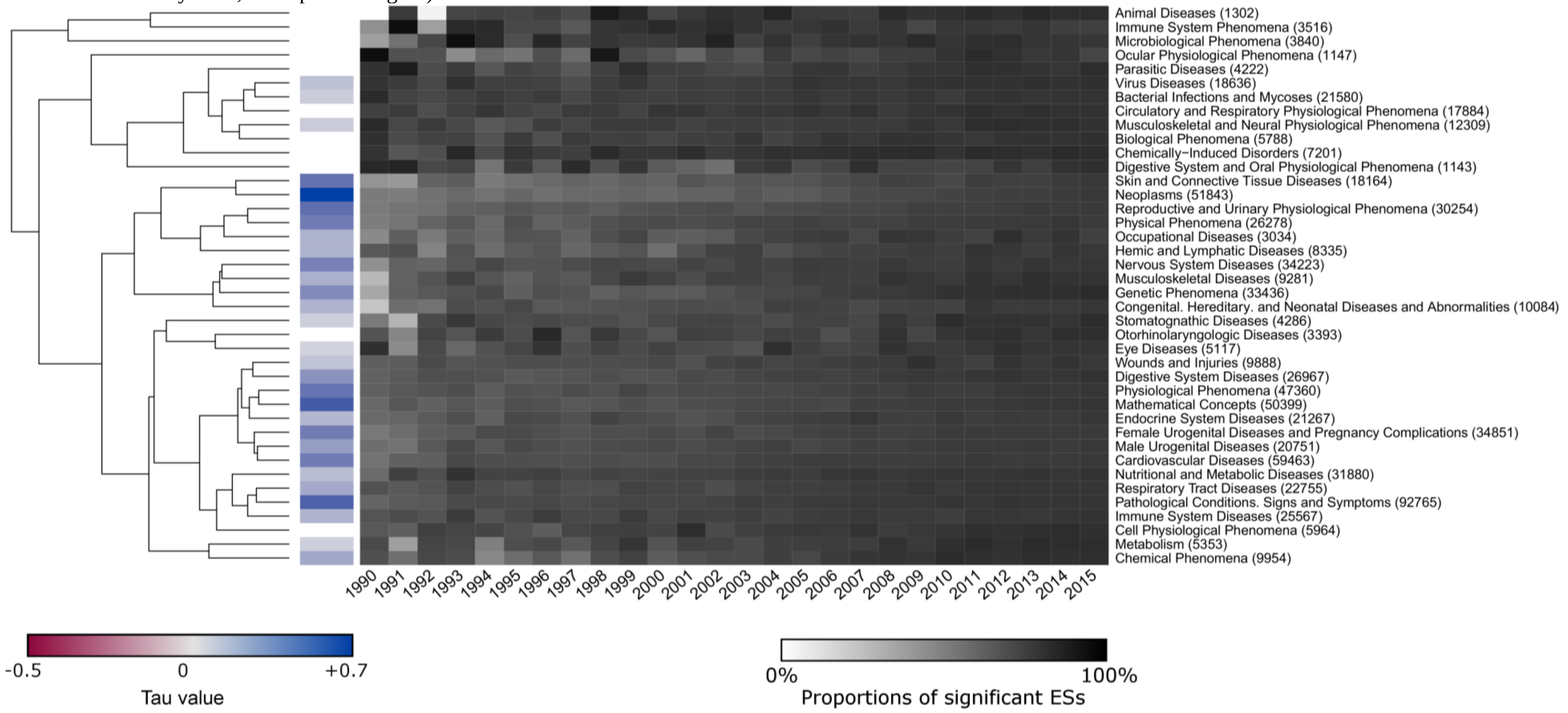

Additional Fig. 5 - ESs decreased over time

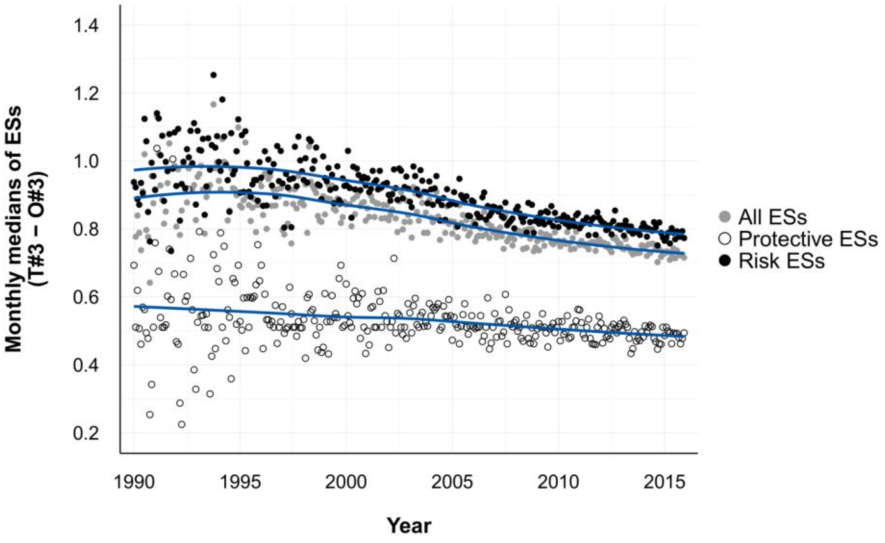

Additional Fig. 5a - Both "protective" and "risk" ESs decreased

Scatter plot of the temporal evolution of monthly medians of ESs (T#3) for all, risk and protective values (gray, black and white-filled, respectively). The decrease of risks was more marked than protective values, with τ values of -0.63 (p<.001), -0.64 (p<.001) and -0.26 (p<.001) for all, risk and protective values respectively. Number of abstracts: 247339.

A "loess" fitted curve was added to scatterplots in order to visualize temporal trends [45].

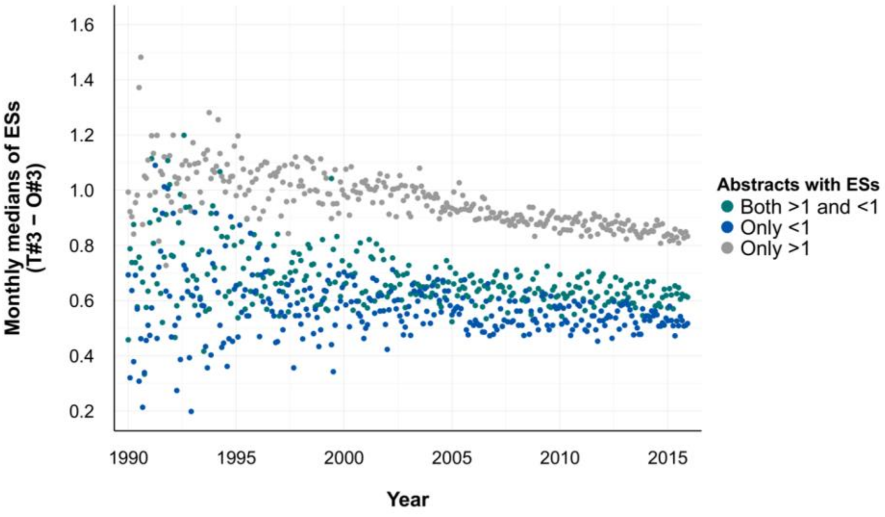

Additional Fig. 5b - ES decreased for both protective and risk values

Monthly trend for medians of ESs (T#3) was decreasing for abstracts with only ESs>1, only ESs<1 or both, with τ values of -0.62 (p<.001), -0.16 (p<.001) and -0.29 (p<.001), respectively. Number of abstracts: 244487 (160013, 38950 and 45524 for abstracts with only ESs>1, only ESs<1 or both, respectively). 2852 abstracts were not represented (abstracts with at least one ES equal to 1).

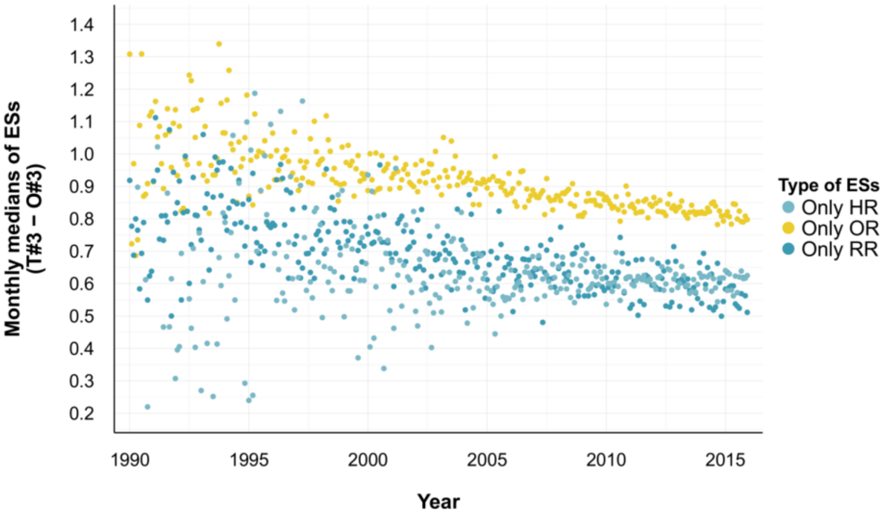

Additional Fig. 5c - HR, OR and RR ESs were decreasing

Scatter plot of the temporal evolution of monthly medians of ESs (T#3) per type of ES. The trend is decreasing for OR, HR and RR, with τ values of -0.59 (p<.001), -0.08 (p<.06) and -0.52 (p<.001), respectively. Six outliers for HR were not shown (value higher than 1.4) but are included in the τ value computations. Number of abstracts: 240170 (157141, 34413 and 48616 for abstracts with only OR, only RR and only HR, respectively). 7169 abstracts were not represented (abstracts with multiple types of ESs).

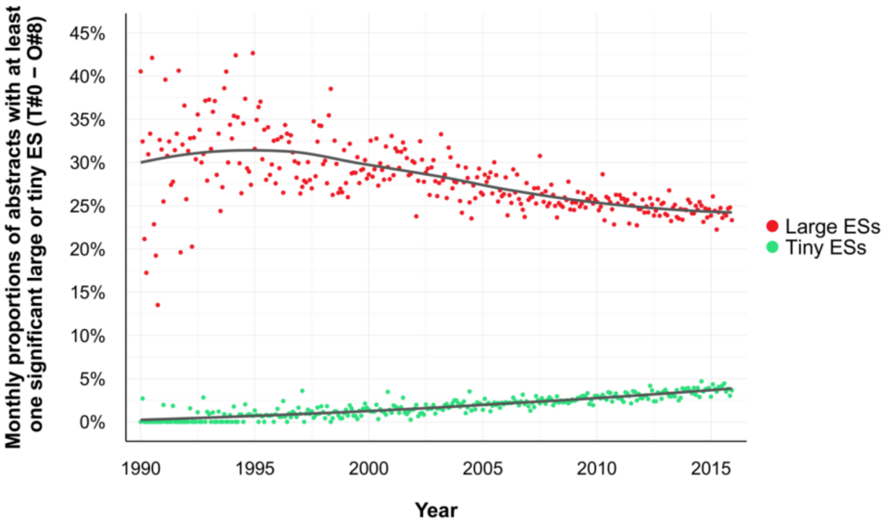

Additional Fig. 5d - The simultaneous increase of statistically significant tiny effects and decrease of statistically significant large effects

Monthly proportion of abstracts with at least one significant large effect (below or equal to 0.2 or greater than 5) or significant tiny effect (between 0.95 and 1.05). τ values were -0.54 (p<.001) and 0.72 (p<.001), respectively. Number of abstracts: 6745 with tiny ESs and 64089 with large ESs.

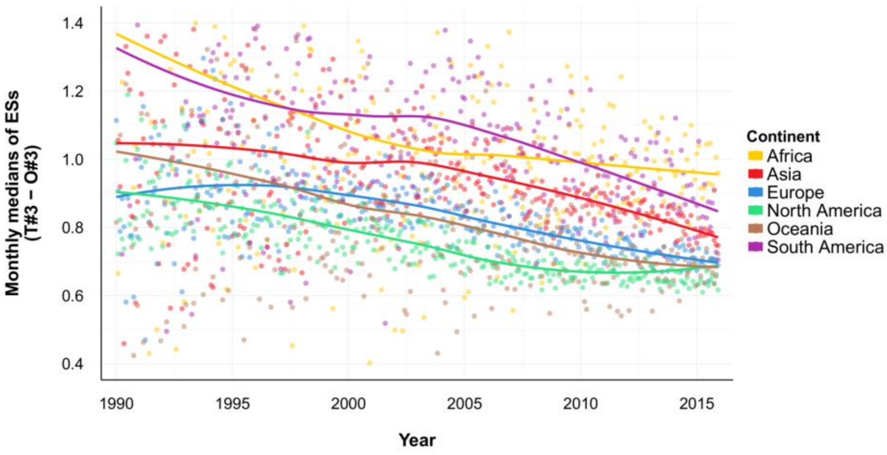

Additional Fig. 5e - ESs decrease in all continents

Monthly medians of ESs values (T#3) by continent. The trend was decreasing for all continents with τ values of -0.14 (p=.014), -0.36 (p<.001), -0.53 (p<.001), -0.63 (p<.001), -0.25 (p<.001) and -0.29 (p<.001), for Africa, Asia, Europe, North America, Oceania and South America, respectively. Values above 1.4 and below 0.4 are not shown but were included for τ value computations. Number of abstracts: 238954 (7136, 48202, 87958, 91867, 9692 and 8771, and abstracts with Africa, Asia, Europe, North America, Oceania and South America, respectively). Abstracts with cross-continental affiliations (5.2% of abstracts) were counted in each continent concerned.

356

Additional Fig. 6 - Knowledge checking

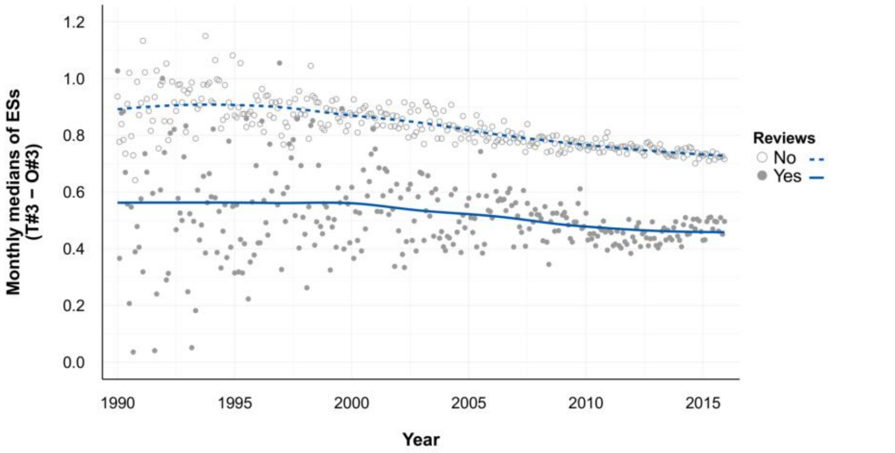

Additional Fig. 6a - ESs from Reviews declined slightly but significantly with time

Scatter plot of temporal evolution of monthly medians of ESs (T#3) found within abstracts of reviews (reviews, systematic reviews or meta-analyses, in gray and solid blue line) or non-reviews (in white and blue dashed line). τ values were -0.16 ($p<.001$) and -0.63 ($p<.001$), respectively. Three outliers are not shown (higher than 1.2) but were included for τ value computations. Number of abstracts: 34846 of reviews and 247339 for non-reviews.

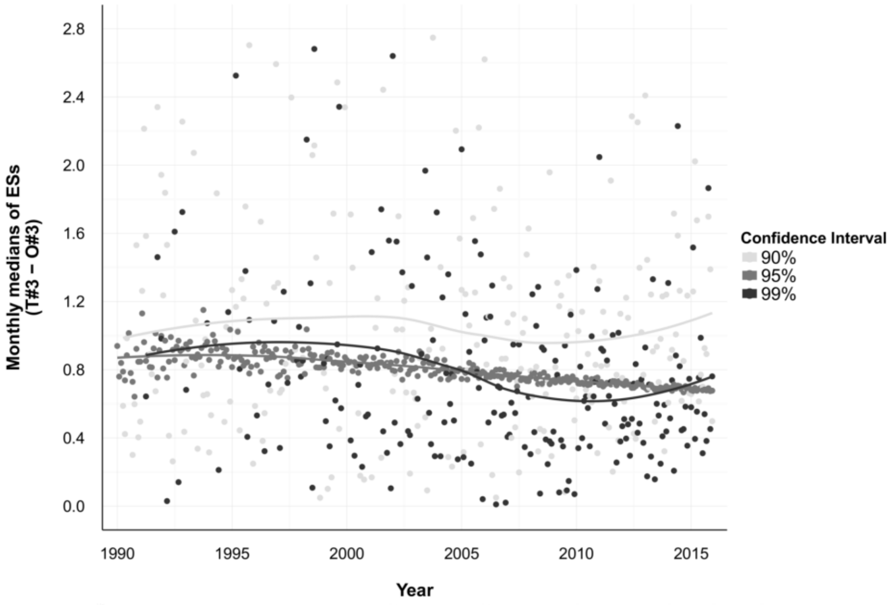

Additional Fig. 6b - ESs decrease was only found for ESs with 95% confidence intervals

Scatter plot of temporal evolution of monthly medians of ESs (T#3) found for abstracts with 90%, 95 or 99% CI (Number of abstracts: 644, 282087 and 579, respectively). No specific trend was found for abstracts with 90% (τ value 0.004, p=.92) or 99% CI (τ value -0.12, p=.01), compared to abstracts with 95%CI (τ value -0.67, $p<.001$). Four outliers are not shown (higher than 2.8) but were included for τ value computations.

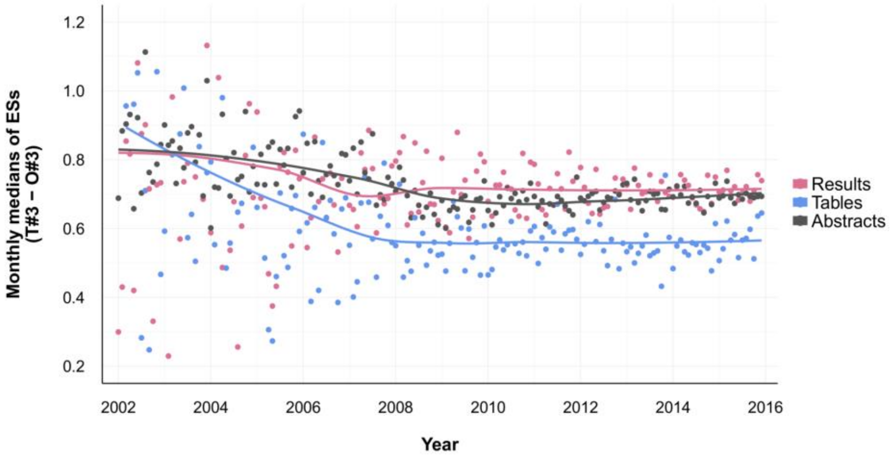

Additional Fig. 6c - Knowledge checking: decrease of ESs was also observed among PMC full-texts

Scatter plot of temporal evolution of monthly medians of ESs values (T#3) for PMC abstracts, results and tables with τ values of -0.37 ($p<.001$), -0.04 (p=.41) and -0.21 ($p<.001$), respectively. Fourteen outliers are not shown (higher than 1.2) but were included for τ value computations. Number of PMC abstracts: 64829 and 25868 with full-text article available in xml format.

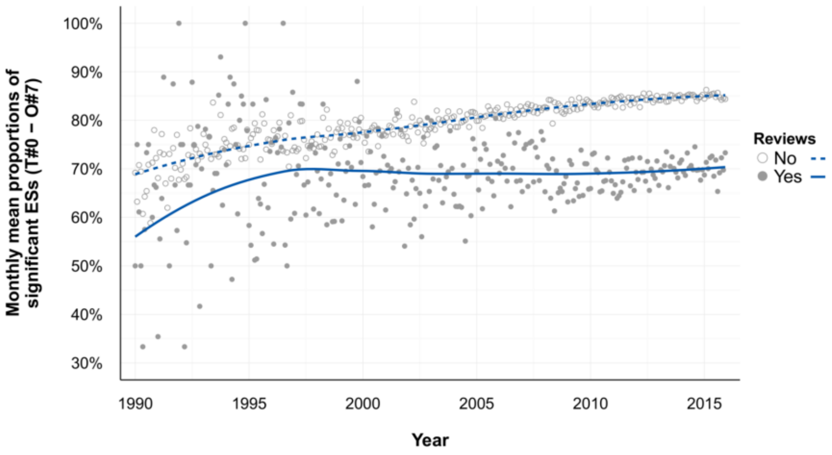

Additional Fig. 6d - The proportion of statistically significant ESs in Reviews remained stable over time

Monthly proportion of statistically significant ESs within abstracts of reviews (reviews, systematic reviews or meta-analyses, in gray and solid blue line) or non-reviews (in white and blue dashed line). τ values were 0.05 (p=.20) and 0.77 (p<.001), respectively. Four outliers are not shown (lower than 30%) but were included for τ value computations. Number of abstracts: 34846 of reviews and 247339 for non-reviews.

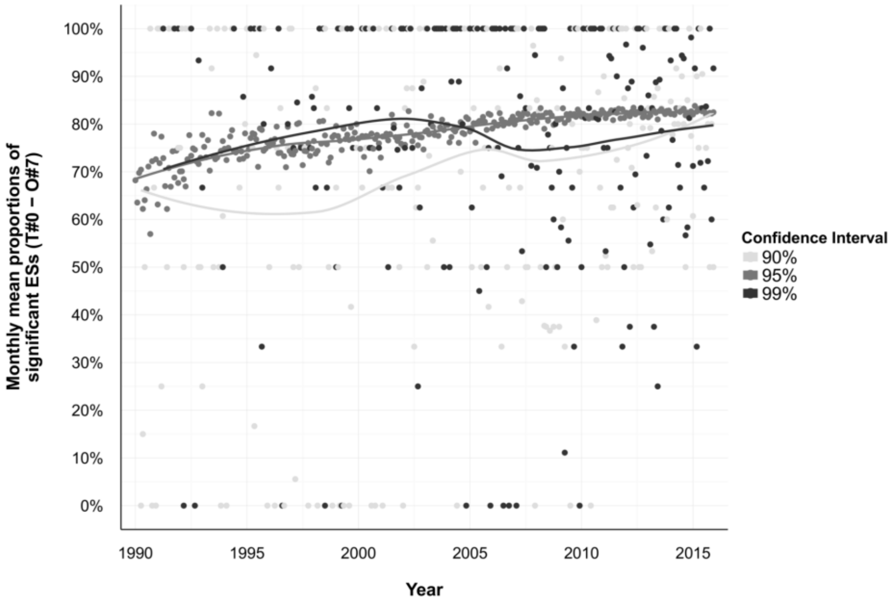

Additional Fig. 6e - No trend was found for 90% and 99% confidence intervals

Monthly proportion of statistically significant ESs found within abstracts with 90%, 95 or 99% CI. No specific trend was obvious for 90% and 99% CI (τ values of 0.07 (p=.14) and -0.06 (p=.22), respectively) compared to 95% (0.70, p<.001). Number of abstracts: 644, 282087 and 579 for 90%, 95% and 99%CI, respectively.

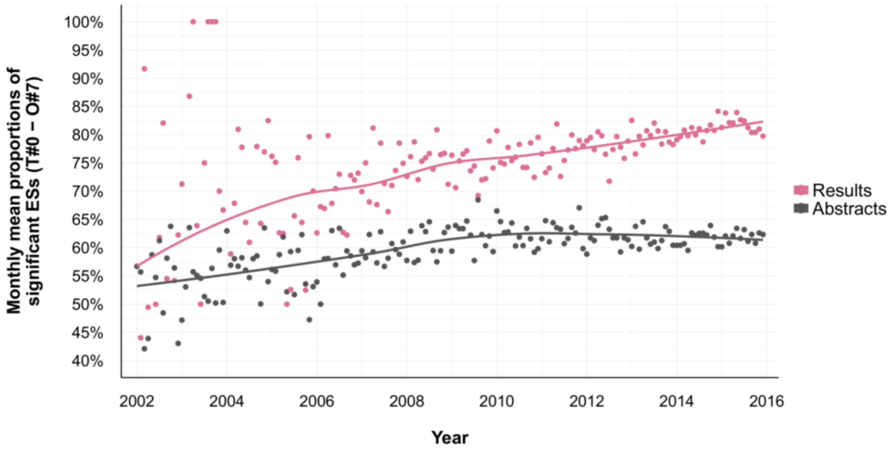

Additional Fig. 6f - The proportion of statistically significant ESs increased over time for values in both Abstracts and Results of PMC full-texts

Monthly proportion of statistically significant ESs for PMC abstracts and results. τ values were 0.41 (p<.001) and 0.50 (p<.001). The Abstracts reported more statistically significant values than the Results did, with a similar increasing trend than the Results. Six outliers are not shown (lower than 40%) but were included for τ value computations. Number of PMC abstracts: 64829 and 25868 with full-text article available in xml format.

358

Additional Fig. 7 - Proportion of statistically significant ESs increased over time

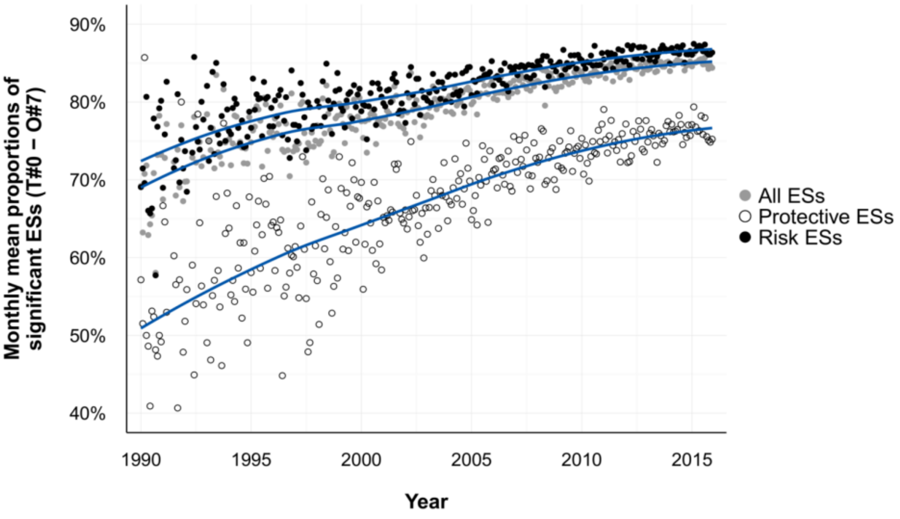

Additional Fig. 7a - Proportion of statistically significant ESs increased for both "protective" and "risk" ESs

Scatter plot of temporal evolution of monthly proportion of statistically significant ESs, for all, risk and protective values (gray, black and white-filled, respectively). τ values were 0.77 ($p<.001$), 0.72 ($p<.001$) and 0.67 ($p<.001$), respectively. Six outliers are not shown (lower than 40%) but were included for τ value computations. Number of abstracts: 247339.

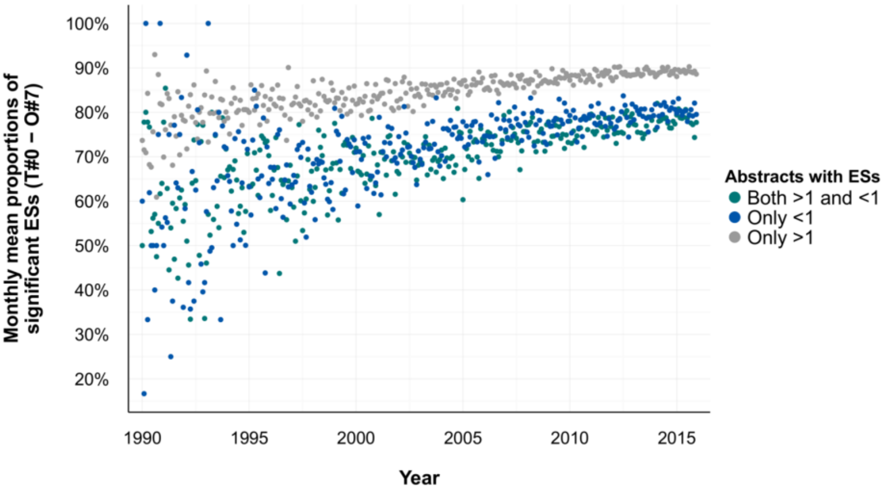

Additional Fig. 7b - Proportion of statistically significant ES increased for both "protective" and "risk" abstracts

Monthly proportion of statistically significant ESs increased for abstracts with only ESs>1, only ESs<1 or both, with τ values of 0.66 ($p<.001$), 0.55 ($p<.001$) and 0.55 ($p<.001$), respectively. Number of abstracts: 244487 (160013, 38950 and 45524 for abstracts with only ESs>1, only ESs<1 or both, respectively). 2852 abstracts were not represented (abstracts with at least one ES equal to 1).

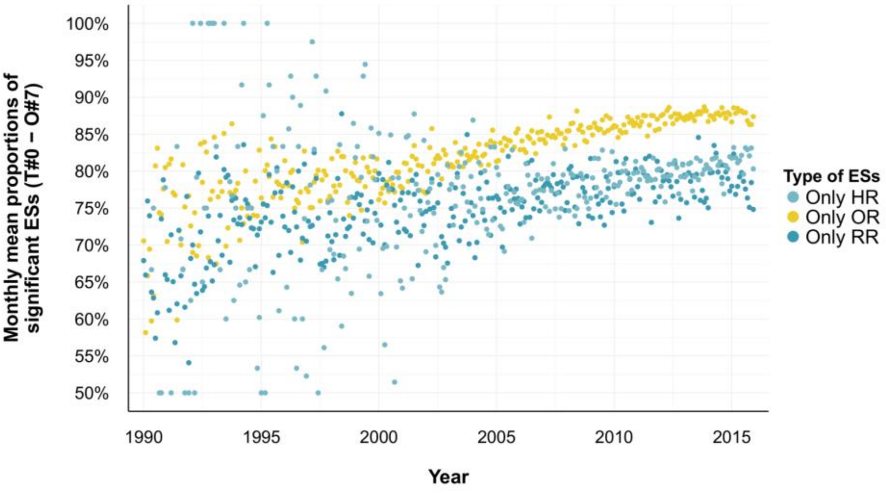

Additional Fig. 7c - Proportion of statistically significant ESs increased whatever the type of ES (HR, OR and RR)

Scatter plot of temporal evolution of monthly proportion of statistically significant ESs per type of ES. The trend is increasing for OR, HR and RR with τ values of 0.72 ($p<.001$), 0.27 ($p<.001$) and 0.42 ($p<.001$), respectively. Thirteen outliers for HR and one for RR are not shown (below 50%) but were included for τ value computations. Number of abstracts: 240170 (157141, 34413 and 48616 for abstracts with only OR, only RR and only HR, respectively). 7169 abstracts were not represented (abstracts with multiple types of ESs).

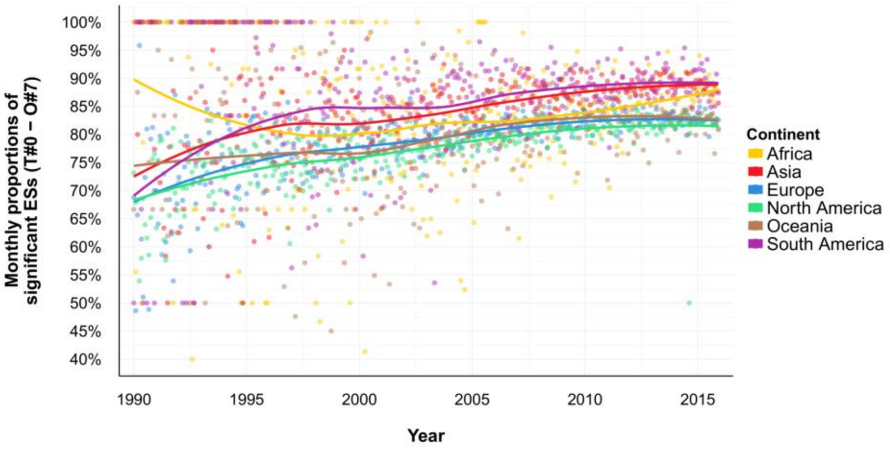

Additional Fig. 7d - Proportion of statistically significant ESs increased for nearly all the geographical areas

Scatter plot of temporal evolution of monthly proportion of statistically significant ESs per geographical area. The trend was increasing for nearly all continents with τ values of -0.01 (p=.73), 0.30 (p<.001), 0.51 (p<.001), 0.62 (p<.001), 0.10 (p=.01) and 0.09 (p=.03), for Africa, Asia, Europe, North America, Oceania and South America, respectively. Number of abstracts: 238954 (7136, 48202, 87958, 91867, 9692 and 8771, and abstracts with Africa, Asia, Europe, North America, Oceania and South America, respectively). Abstracts with cross-continental affiliations (5.2% of abstracts) were counted in each continent concerned.

360

Supplementary File 1

Performance testing: kappa, sensitivity and specificity

Output PDF file from Perl script, containing the summary of values obtained for kappa, sensitivity and specificity. The screened PMIDs are provided with special emphasis on false negative, false positive and discordant results.

Supplementary References

46. Torvik VI. MapAffil: A Bibliographic Tool for Mapping Author Affiliation Strings to Cities and Their Geocodes Worldwide. -Lib Mag. Mag. Digit. Libr. Forum. 2015;21.

47. Schuyler PL, Hole WT, Tuttle MS, Sherertz DD. The UMLS Metathesaurus: representing different views of biomedical concepts. Bull. Med. Libr. Assoc. 1993;81:217–22.

48. Liljekvist MS, Andresen K, Pommergaard H-C, Rosenberg J. For 481 biomedical open access journals, articles are not searchable in the Directory of Open Access Journals nor in conventional biomedical databases. PeerJ. 2015;3:e972.

49. Menard S. Logistic Regression: From Introductory to Advanced Concepts and Applications. SAGE; 2010.

50. Cooper H. The Handbook of Research Synthesis. 1st ed. Russell Sage Foundation Publications; 1994.
