# Supplementary material for: The intriguing evolution of effect sizes in biomedical research over time: smaller but more often statistically significant
Source: Gigascience. 2017 Dec 6;7(1):gix121. doi: 10.1093/gigascience/gix121 (PMC5765564; doi:10.1093/gigascience/gix121)
Supplement: Response_to_Reviewer_Comments_Original_Submission.pdf [file gix121_response_to_reviewer_comments_original_submission.pdf]

Author's Response To Reviewer Comments

Dear Editorial Team of GigaScience,
We would like to thank the reviewers for the time they spent on their careful examination of our manuscript and their very constructive and insightful comments and suggestions.
Our responses to the reviewers are noted point by point in the Personal Cover entitled "Response to reviewers".
