# Supplementary material for: The intriguing evolution of effect sizes in biomedical research over time: smaller but more often statistically significant
Source: Gigascience. 2017 Dec 6;7(1):gix121. doi: 10.1093/gigascience/gix121 (PMC5765564; doi:10.1093/gigascience/gix121)
Supplement: Response_to_Reviewer_Comments_Revision_1.pdf [file gix121_response_to_reviewer_comments_revision_1.pdf]

Author's Response To Reviewer Comments

Dear Editorial Team of GigaScience,
We would like to thank the reviewers for the time they spent on their careful examination of our manuscript and their very constructive and insightful comments and suggestions. Our response to the reviewer is noted in the response to reviewers file. The change is printed in red, both in the corresponding file and in the manuscript. We hope that this modification made will be satisfactory. We have also organized the supporting data with the help of the GigaScience Database team, and proposed a simplified version of the title.
