# Supplementary material for: The intriguing evolution of effect sizes in biomedical research over time: smaller but more often statistically significant
Source: Gigascience. 2017 Dec 6;7(1):gix121. doi: 10.1093/gigascience/gix121 (PMC5765564; doi:10.1093/gigascience/gix121)
Supplement: Reviewer_1_Report_(Original_Submission) -- Shinichi Nakagawa — 20 Aug 2017 Reviewed [file gix121_reviewer_1_report_(original_submission).pdf]

Reviewer Report

Title: The intriguing evolution of effect sizes in biomedical research over time: smaller but more often statistically significant

Version: Original Submission **Date:** 20 Aug 2017

Reviewer name: Shinichi Nakagawa

Reviewer Comments to Author:

This interesting meta-research uses text-mining techniques to study how the magnitude of effect sizes and p values changed over time since 1990 (up to 2015). Interestingly, they found that the average effect size value declined over time while the percentage of significant results increased. Their data is impressive and the method is described in details. The paper is very well written. However, I have two major concerns.

One --- The authors mixed risk ratios (RRs), odds ratios (ORs) and hazard ratios (HRs). This shortcoming was not really clear till towards the end of Discussion. This shortcoming should be informed to the reader much earlier. In Page 7 (Line 36 onwards), it says "we could not perform data mining on all types of ESs with sufficient accuracy to guarantee the best ...." I do not really understand this sentence. The best way to go is that the authors analyse RRs, ORs and HRs separately and show the results are consistent over these 3 metrics. One just cannot really mix these metrics as they can be quite different in magnitudes.

Two --- Their main results can have alternative explanations and they need to be explored and mentioned. ESs can become smaller assuming that more recent studies use larger sample sizes and there existed publication bias. It is well-known with small sample sizes, we are more likely to get over-estimated effect sizes. Can the authors examine a time trend in sample sizes? Also, statistical significance is a function of sample sizes so if the sample size is increasing over time, finding more significant results may not be as surprising as the authors made it out to be.

Methods

Are the methods appropriate to the aims of the study, are they well described, and are necessary controls included? No

Conclusions

Are the conclusions adequately supported by the data shown? No

Reporting Standards

Does the manuscript adhere to the journal’s guidelines on minimum standards of reporting? Yes

Statistics

Are you able to assess all statistics in the manuscript, including the appropriateness of statistical tests used? Yes, and I have assessed the statistics in my report.

Quality of Written English

Please indicate the quality of language in the manuscript: Acceptable

Declaration of Competing Interests

Please complete a declaration of competing interests, considering the following questions:

- Have you in the past five years received reimbursements, fees, funding, or salary from an organisation that may in any way gain or lose financially from the publication of this manuscript, either now or in the future?
- Do you hold any stocks or shares in an organisation that may in any way gain or lose financially from the publication of this manuscript, either now or in the future?
- Do you hold or are you currently applying for any patents relating to the content of the manuscript?
- Have you received reimbursements, fees, funding, or salary from an organization that holds or has applied for patents relating to the content of the manuscript?
- Do you have any other financial competing interests?
- Do you have any non-financial competing interests in relation to this paper?

If you can answer no to all of the above, write 'I declare that I have no competing interests' below. If your reply is yes to any, please give details below.

None

I agree to the open peer review policy of the journal. I understand that my name will be included on my report to the authors and, if the manuscript is accepted for publication, my named report including any attachments I upload will be posted on the website along with the authors' responses. I agree for my report to be made available under an Open Access Creative Commons CC-BY license (http://creativecommons.org/licenses/by/4.0/). I understand that any comments which I do not wish to be included in my named report can be included as confidential comments to the editors, which will not be published.

I agree to the open peer review policy of the journal.

To further support our reviewers, we have joined with Publons, where you can gain additional credit to further highlight your hard work (see: https://publons.com/journal/530/gigascience). On publication of

this paper, your review will be automatically added to Publons, you can then choose whether or not to claim your Publons credit. I understand this statement.

Yes
