# Supplementary material for: The intriguing evolution of effect sizes in biomedical research over time: smaller but more often statistically significant
Source: Gigascience. 2017 Dec 6;7(1):gix121. doi: 10.1093/gigascience/gix121 (PMC5765564; doi:10.1093/gigascience/gix121)
Supplement: Reviewer_1_Report_(Revision_1) -- Shinichi Nakagawa — 03 Nov 2017 Reviewed [file gix121_reviewer_1_report_(revision_1).pdf]

Reviewer Report

Title: The intriguing evolution of effect sizes in biomedical research over time: smaller but more often statistically significant

Version: Revision 1 **Date:** 03 Nov 2017

Reviewer name: Shinichi Nakagawa

Reviewer Comments to Author:

I have read their comprehensive point-by-point replies for my comments as well as the other reviewer's comments. I think the authors did a wonderful job of it. I find it interesting that the authors did find sample sizes are increasing, which I think is a good thing for robustness in general.

Given that earlier studies actually are based smaller sample sizes (more sampling errors) and this MS uses the absolute values of effect sizes, I can actually say this trend of a decline in effect size may be overestimated. To see why please see this paper.

Morrissey, M. B. 2016. Meta-analysis of magnitudes, differences and variation in evolutionary parameters. J Evol Biol 29:1882-1904.

We have actually written a piece which will explain this in a more intuitive way.

Nakagawa, S. & Lagisz, M. (2016) Visualising unbiased and biased unweighted meta-analysis. Journal of Evolutionary Biology. 29: 1914-1916

I think the authors probably should mention analyses of absolute values of effect sizes need to be carefully done.

Methods

Are the methods appropriate to the aims of the study, are they well described, and are necessary controls included? Yes

Conclusions

Are the conclusions adequately supported by the data shown? Yes

Reporting Standards

Does the manuscript adhere to the journal's guidelines on minimum standards of reporting? Yes

Statistics

Are you able to assess all statistics in the manuscript, including the appropriateness of statistical tests used? Yes, and I have assessed the statistics in my report.

Quality of Written English

Please indicate the quality of language in the manuscript: Acceptable

Declaration of Competing Interests

Please complete a declaration of competing interests, considering the following questions:

- Have you in the past five years received reimbursements, fees, funding, or salary from an organisation that may in any way gain or lose financially from the publication of this manuscript, either now or in the future?
- Do you hold any stocks or shares in an organisation that may in any way gain or lose financially from the publication of this manuscript, either now or in the future?
- Do you hold or are you currently applying for any patents relating to the content of the manuscript?
- Have you received reimbursements, fees, funding, or salary from an organization that holds or has applied for patents relating to the content of the manuscript?
- Do you have any other financial competing interests?
- Do you have any non-financial competing interests in relation to this paper?

If you can answer no to all of the above, write 'I declare that I have no competing interests' below. If your reply is yes to any, please give details below.

None

I agree to the open peer review policy of the journal. I understand that my name will be included on my report to the authors and, if the manuscript is accepted for publication, my named report including any attachments I upload will be posted on the website along with the authors' responses. I agree for my report to be made available under an Open Access Creative Commons CC-BY license (http://creativecommons.org/licenses/by/4.0/). I understand that any comments which I do not wish to be included in my named report can be included as confidential comments to the editors, which will not be published.

I agree to the open peer review policy of the journal

To further support our reviewers, we have joined with Publons, where you can gain additional credit to further highlight your hard work (see: https://publons.com/journal/530/gigascience). On publication of this paper, your review will be automatically added to Publons, you can then choose whether or not to claim your Publons credit. I understand this statement.

Yes
