# Supplementary material for: The intriguing evolution of effect sizes in biomedical research over time: smaller but more often statistically significant
Source: Gigascience. 2017 Dec 6;7(1):gix121. doi: 10.1093/gigascience/gix121 (PMC5765564; doi:10.1093/gigascience/gix121)
Supplement: Reviewer_2_Report_(Original_Submission) -- BjÖrn Brembs — 13 Sep 2017 Reviewed [file gix121_reviewer_2_report_(original_submission).pdf]

Reviewer Report

Title: The intriguing evolution of effect sizes in biomedical research over time: smaller but more often statistically significant

Version: Original Submission **Date:** 13 Sep 2017

Reviewer name: Björn Brembs

Reviewer Comments to Author:

This is an interesting piece of work looking at the evolution of effect sizes over time. Such data is highly relevant, important and insightful.

The formatting of this manuscript was not very conducive to peer-review, with the figures attached at the end, separated from the legends:
http://bjoern.brembs.net/2015/02/publishers-stop-torturing-your-reviewers/
This makes it very tedious to review and I have not looked at the figures. Therefore, I only have one main conceptual issue and some minor comments on the text. If the authors or the editor are interested in my opinion on the data/figures, please provide the manuscript in an easy-to-review format.

Major:
The authors should make a better effort at distinguishing reporting from actual values. Two examples:
- For the papers where only abstracts were evaluated, we don't know if the choice of which values to put in the abstract changed in the period observed. Hence, all these values are likely an author-selected subset of the total values (and the total values in the paper likely again a subset of the values ever obtained by the researchers, cf. file-drawer effect/publication bias)
- Statistical significance was not evaluated as reported in the source papers, but defined for this work as "if the CI did not encompass 1." Thus, statistical significance may or may not fit what the original papers reported, depending on the significance criteria of the source authors.
One can tell from some of the wordings that the authors are aware of this distinction, but a more explicit effort has to be made in order to make sure the reader is never in doubt as to whether the authors are referring to effects due to changing reporting practices or due to actual differences in the effect sizes or significance. Importantly, whenever the two cannot be distinguished, other data may be used to try and disambiguate. For instance one explanation for actually decreasing effect sizes and increasing significance may be an increase in statistical power. However, there is some evidence that power has not increased over the last decades, suggesting that these trends may be due to reporting changes.
The authors may not share this interpretation - it is just an example of how to better treat this crucial distinction that plagues any such study.

Minor:
"KDD process" not explained at first mention (only in the Methods section).

Methods ought to be before results (journal instructions?), for a better understanding of how the results were obtained and what to make of them.

Björn Brembs

Methods

Are the methods appropriate to the aims of the study, are they well described, and are necessary controls included? Yes

Conclusions

Are the conclusions adequately supported by the data shown? No

Reporting Standards

Does the manuscript adhere to the journal's guidelines on minimum standards of reporting? Yes

Statistics

Are you able to assess all statistics in the manuscript, including the appropriateness of statistical tests used? Yes, and I have assessed the statistics in my report.

Quality of Written English

Please indicate the quality of language in the manuscript: Acceptable

Declaration of Competing Interests

Please complete a declaration of competing interests, considering the following questions:

- Have you in the past five years received reimbursements, fees, funding, or salary from an organisation that may in any way gain or lose financially from the publication of this manuscript, either now or in the future?
- Do you hold any stocks or shares in an organisation that may in any way gain or lose financially from the publication of this manuscript, either now or in the future?
- Do you hold or are you currently applying for any patents relating to the content of the manuscript?
- Have you received reimbursements, fees, funding, or salary from an organization that holds or has applied for patents relating to the content of the manuscript?
- Do you have any other financial competing interests?

- Do you have any non-financial competing interests in relation to this paper?

If you can answer no to all of the above, write 'I declare that I have no competing interests' below. If your reply is yes to any, please give details below.

I declare that I have no competing interests

I agree to the open peer review policy of the journal. I understand that my name will be included on my report to the authors and, if the manuscript is accepted for publication, my named report including any attachments I upload will be posted on the website along with the authors' responses. I agree for my report to be made available under an Open Access Creative Commons CC-BY license (http://creativecommons.org/licenses/by/4.0/). I understand that any comments which I do not wish to be included in my named report can be included as confidential comments to the editors, which will not be published.

I agree to the open peer review policy of the journal

To further support our reviewers, we have joined with Publons, where you can gain additional credit to further highlight your hard work (see: https://publons.com/journal/530/gigascience). On publication of this paper, your review will be automatically added to Publons, you can then choose whether or not to claim your Publons credit. I understand this statement.

Yes
